# Amination–degradation of super engineering plastics for the construction of surface emissive resin materials

**DOI:** 10.1038/s42004-026-02051-1

**Published:** 2026-04-30

**Authors:** Yasunori Minami, Shunsuke Tsuyuki, Ryota Watanabe, Nobuyasu Itoh, Masaru Yoshida

**Affiliations:** 1 Integrated Research Center for Circular Technology, National Institute of Advanced Industrial Science and Technology (AIST), Tsukuba Central 5, Tsukuba, Ibaraki Japan; 2 Graduate School of Pure and Applied Science Department, University of Tsukuba, Tsukuba, Ibaraki Japan; 3 PRESTO, Japan Science and Technology Agency (JST), Tsukuba, Ibaraki Japan; 4 Institute for Chemical Process Technology, National Institute of Advanced Industrial Science and Technology (AIST), Tsukuba Central 5, Tsukuba, Ibaraki Japan; 5 Research Institute for Sustainable Chemistry, National Institute of Advanced Industrial Science and Technology (AIST), Tsukuba Central 5, Tsukuba, Ibaraki Japan; 6 National Metrology Institute of Japan, National Institute of Advanced Industrial Science and Technology (AIST), Tsukuba Central 3, Tsukuba, Ibaraki Japan; 7 Catalytic Chemistry Research Institute, National Institute of Advanced Industrial Science and Technology (AIST), Tsukuba Central 5, Tsukuba, Ibaraki Japan

**Keywords:** Polymer synthesis, Sustainability, Fluorescent labelling

## Abstract

The advent of effective use of carbon resources has demanded for the future society. This importance is evident from the recent evolution of the “R” framework such as “10 R” including “Repurpose”. In this context, super engineering plastics such as polyetheretherketone (PEEK) are considered as promising plastic materials because they can be used as monomaterials, i.e., without additives, by virtue of their high stability, which also renders them suitable to be converted to functionalized recyclable plastics. Herein, we report a degradation methodology for PEEK and polysulfone (PSU) resins using aza-aromatic compounds such as phenothiazine. This amination–degradation reaction cleaves the polymer main chains to form donor–acceptor–donor-type molecules, which allow functionalizing only the resin surface to produce luminescent PEEK powder and plates. These surface-functionalized PEEK materials maintain high thermal stability and are applicable as photoredox catalysts, demonstrating the “Repurpose” of PEEK into value-added products.

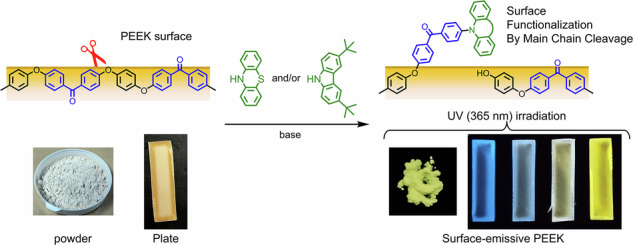

## Abstract (web summary)

Super engineering plastics like PEEK are extraordinarily stable, which has hindered efforts to convert them into recyclable materials. This work presents a degradation strategy for PEEK and PSU resins using the aza-aromatic compounds phenothiazine, yielding luminescent and thermally robust products that serve as effective photoredox catalysts.

## Introduction

To realize an effective use of carbon resources, the implementation of the recent evolved “10 R” framework including “Repurpose”: recreation into something new, based on 3 R (“reduce, reuse, and recycle”) of existing carbonaceous materials such as plastics is essential. In this context, advances in chemical transformation processes enhance the potential for “Repurpose” and pave the way toward the development of innovative methods for the valorization of petroleum products^1–6^.

**Fig. 1:**
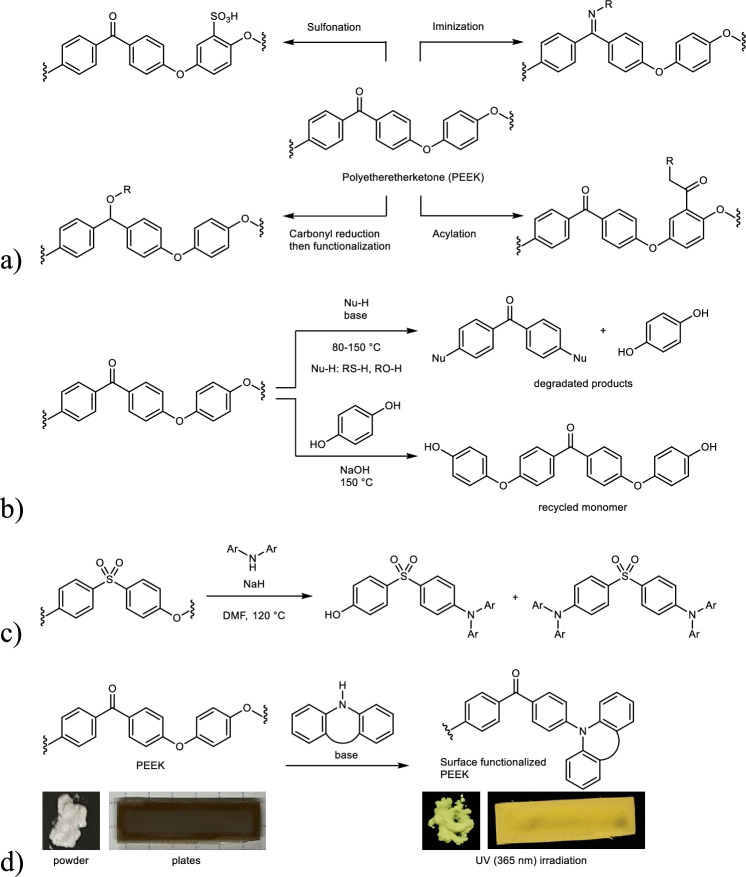
Transformation of polyetheretherketone (PEEK). **a** Functionalization of PEEK via sulfonation, iminization, carbonyl reduction, and acylation. **b** Previous work: Degradation and depolymerization of super engineering plastics using various nucleophiles. **c** Preparation of luminescent compounds via the reaction of polyethersulfone (PESU) with electron-rich amines. **d** This work: Degradation of PEEK using azacycles for surface functionalization to produce donor–acceptor–donor-type light–emissive PEEK materials. Nu–H nucleophile, RS–H organic thiol, RO–H alcohol, aryl alcohol, or alkali hydroxide, Ar aryl, UV ultraviolet.

Super engineering plastics such as polyetheretherketone (PEEK) are interesting materials for the investigation of plastic degradation methods owing to their high stability. Particularly, PEEK exhibits not only thermal stability but also insolubility in organic solvents, chemical resistance, high melting point, and mechanical strength without requiring additives. Accordingly, PEEK is used in equipment requiring high stability, e.g., medical devices, automobiles, and aircraft. This outstanding stability stems from the robust benzene ring–based molecular structure linked by stable carbon–oxygen bonds in the main chains. To fully exploit this property, various surface functionalization methods for PEEK have been developed^7–9^, such as sulfonation, sulfonic acid group (SO_3_H) into the hydroquinone ring (Fig. 1a)^10–12^, plasma treatment to generate hydroxy groups on the PEEK surface^13^, iminization^14,15^ and reduction of the carbonyl group followed by substitution^16,17^, Friedel–Crafts acylation to introduce carbonaceous groups^18–20^, and photoinduced graft polymerization using electron-deficient alkenes^21,22^. These elegant methodologies are considered effective in contributing to “Repurpose”. Notably, these methods are based on the functionalization of the main chain. From the viewpoint, the development of facile functionalization methods while preserving the main-chain structure are expected to attribute to the advancement of “Repurpose”.

Recently, we reported the degradation of super engineering plastics such as PEEK using nucleophiles, bases, and polar amide solvents to cleave the stable main-chain without damaging its molecular structure to form monomeric products (Fig. 1b)^23–27^. Especially, depolymerization underwent with hydroquinone to form bisphenol-type recycled monomer composed of original PEEK framework^27^. This methodology can be envisaged as a resin functionalization method that preserves the molecular structure of the main-chain. Although this method causes a decrease in molecular weight, the durability of highly stable super engineering plastics could be maintained. Since our reports, several studies on the effective degradation of super engineering plastics were reported^28–31^. Especially, Dufaud, Raynaud, and Monteil et al. reported the fabrication of luminescent compounds via the reaction of polyethersulfone (PESU) with electron-rich amines (Fig. 1c)^31^, whereas simple aminolysis of polysulfone (PSU) and PESU results in low yields of low-weight molecules^32^. These results can be considered as a proof of concept of our idea. Herein, we report transformation methods of PEEK aimed at surface functionalization based on the cleavage of main chains. First, we verified the degradation of PSU, PEES, and PEEK using azacyclic compounds and bases to provide donor–acceptor–donor-type molecules. This examination aimed to understand the conditions necessary for proceeding moderately and precisely. Then, we applied this degradation to functionalize the PEEK resin surface gently to form luminescent PEEK powder and plates having D-A moiety bond to the rest of the polymer chain (Fig. 1d). The prepared luminescent PEEK materials maintained high thermal stability and were applied not only to fine powders but also to pellets and sheet materials.

## Results and Discussion

### Screening the degradation conditions

Toward approaches and verification of degradable surface functionalization, first, we examined the normal degradation of PSU pellets (**1**) consisting of bisphenol S (4,4’-sulfonyldiphenol, BPS) and bisphenol A (4,4’-(propane-2,2-diyl)diphenol; BPA, **4**) units in the alternating chain, 3,6-di-*tert*-butyl carbazole (**2a**) (2.5 equiv. relative to the BPS and BPA units), and potassium *tert*-butoxide (KO*t*Bu) (2.4 equiv.) in dehydrated 1,3-dimethyl-2-imidazolidinone (DMI, 0.4 mL) to form photo emissive chemicals. They were placed in a glass vial with a PTFE septum cap under an argon atmosphere at ambient pressure and stirred at 120 °C for 30 min, followed by treatment with HCl. As a result, the degradation products 9,9’-(sulfonylbis(4,1-phenylene))bis(3,6-di-*tert*-butyl-9H-carbazole) (**3a**) as a donor–acceptor–donor-type fluorescent organic material^33^ and **4** were formed in 39% and 33% yields, respectively, as determined by ^1^H NMR spectroscopic analysis (Table 1, Entry 1). Optimization of the reaction time resulted in the quantitative formation of **3a** and **4** within 5 h (Table 1, Entries 2–4; Supplementary Figs. S1 and S2). Reducing the amount of **2a** and KO*t*Bu, the concentration, or the temperature decreased the yields of **3a** and **4** (Table 1, Entries 5–7). Various solvents, such as *N*-methyl-2-pyrrolidone (NMP), *N*,*N*-dimethylacetamide (DMAc), 1,4-dioxane, and toluene, were examined (Table 1, Entries 8–11), finding that amide solvents were effective. In addition, a screening of inorganic bases such as NaOH, KOH, K_3_PO_4_, and NaO*t*Bu (Table 1, Entries 12-15) revealed that strong bases were applicable, with NaO*t*Bu enabling a smooth degradation. Finally, we selected the conditions described in Table 1, Entry 4 (2.5 equiv. of **2a** and KO*t*Bu in DMI solvent) for the degradation.

**Table 1:** Optimization of the reaction of PSU pellets **1** with carbazole **2a**^a^


Entry	2a (equiv.)	Base (equiv.)	Solvent (mL)	Temp.	Time	3a (%)	4 (%)
1	2.5	KO*t*Bu (2.4)	DMI (0.4)	120 °C	0.5 h	39	33
2	2.5	KO*t*Bu (2.4)	DMI (0.4)	120 °C	1 h	68	60
3	2.5	KO*t*Bu (2.4)	DMI (0.4)	120 °C	2 h	75	62
4	2.5	KO*t*Bu (2.4)	DMI (0.4)	120 °C	5 h	> 99 (90)	> 99 (90)
5	2	KO*t*Bu (2.1)	DMI (0.4)	120 °C	5 h	86	82
6	2	KO*t*Bu (3)	DMI (2.0)	120 °C	16 h	47	70
7	2.5	KO*t*Bu (2.4)	DMI (0.4)	100 °C	5 h	66	58
8	2.5	KO*t*Bu (2.4)	NMP (0.4)	120 °C	5 h	77	90
9	2.5	KO*t*Bu (2.4)	DMAc (0.4)	120 °C	5 h	95	94
10	2.5	KO*t*Bu (2.4)	1,4-dioxane (0.4)	120 °C	5 h	n.d.	n.d.
11	2.5	KO*t*Bu (2.4)	toluene (0.4)	120 °C	5 h	n.d.	n.d.
12	2.5	KOH (2.4)	DMI (0.4)	120 °C	5 h	58	60
13	2.5	K_3_PO_4_ (2.4)	DMI (0.4)	120 °C	5 h	13	6
14	2.5	NaOH (2.4)	DMI (0.4)	120 °C	5 h	55	56
15	2.5	NaO*t*Bu (2.4)	DMI (0.4)	120 °C	5 h	94	> 99

^a^Unless otherwise noted, a mixture of **1** (pellets, 0.2 mmol relative to the molecular weight of the monomer), **2a** (0.5 mmol), base (0.48 mmol), and solvent (0.4 mL) was stirred. The resultant mixture was quenched using aq. HCl. Yields were determined by ^1^H NMR spectroscopy. *t*Bu *tert*-butyl.

### Substrate scope for the formation of D-A-D type molecules

**Fig. 2:**
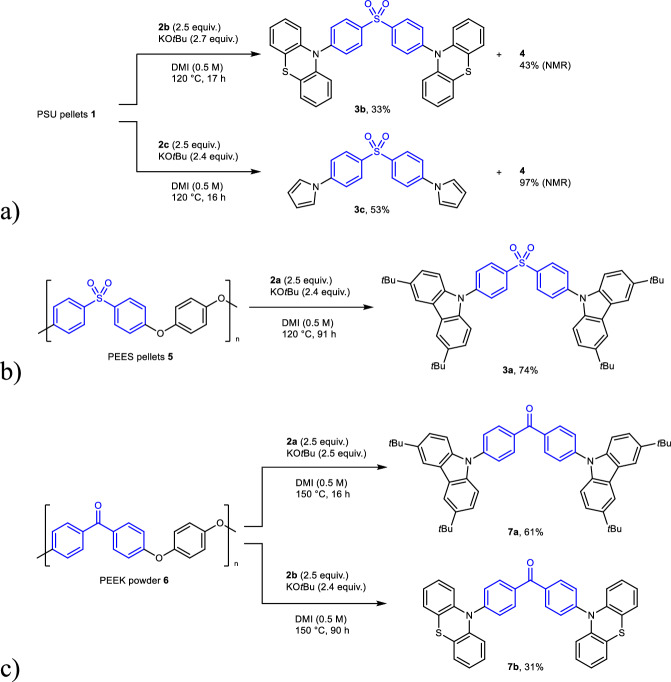
Scope of the degradation of super engineering plastics to form donor–acceptor–donor-type molecules. **a** Degradation of PSU pellets **1** with **2b** or **2c**. **b** Degradation of PEES pellets **5** with **2a**. **c** Degradation of PEEK powder **6** with **2a** or **2b**.

Next, we evaluated the degradation of PSU pellets **1** in terms of amine compounds. Phenothiazine (PTZ, **2b**) showed lower reactivity toward the degradation, maybe due to steric bulkiness, but the corresponding product **3b**^34–36^ and **4** were obtained in 33% and 43% yields, respectively (Fig. 2a). When pyrrole (**2c**) was used, the degradation proceeded to form **3c** and **4**. The poor solubility of product **3c** hindered its isolation; therefore, a moderate isolated yield of **3c** was obtained via recrystallization. Conversely, aromatic amines resulted in a decrease in the yield of deaminated products (Supplementary Fig. S3). Poly(1,4-phenylene ether ether sulfone) (PEES) pellets **5** underwent the degradation of **2a** to form **3a** in 74% yield (Fig. 2b). Meanwhile, the reaction of PEEK powder **6** with **2a** and **2b** led to the corresponding products **7a** and **7b** (Fig. 2c). According to previous studies, the degradation reactivity of PEEK was lower than that of PSU and PEES because of its insolubility in solvents and the weak electron-withdrawing ability of the carbonyl group. Finally, increasing the reaction temperature to 150 °C afforded the fluorescent donor–acceptor–donor-type products **7a**^37,38^ and **7b**^35,39^ in comparable yields to those obtained with PSU and PEES. Of note, hydroquinone was detected by ^1^H NMR spectroscopic analyses after the degradation of **5** and **6**.

### Reactivity of ether cleavage

**Fig. 3:**
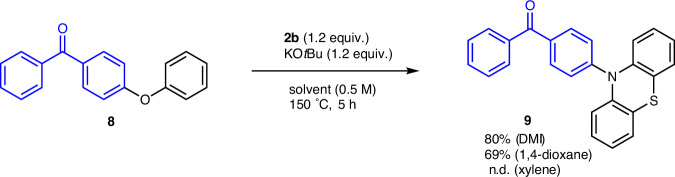
Solvent effect on the reactivity toward C–O bond cleavage. Reaction of 4-phenoxybenzophenone with **2b** (1 equiv.) mediated by KO*t*Bu in various solvents at 150 °C for 5 h.

To gain insight into the inherent reactivity toward C–O bond cleavage, we examined the reaction of 4-phenoxy-benzophenone (**8**) with 1 equiv. of **2b** and KO*t*Bu in various solvents at 150 °C for 5 h (Fig. 3). The reactions in DMI solvent produced a homogeneous deep red solution, from which **9** was obtained in 80% yield. The smooth reactivity of small substrate **8** confirmed that the steric bulkiness of **2b** decreased the reactivity in the case of the polymers. In the case using DMAc solvent, complex mixture was generated, suggesting that DMAc solvent is not suitable for the degradation of PEEK with azacycles. In fact, the reaction of PEEK powder **6** with **2b** and KO*t*Bu in DMAc hardly proceeded unlike the case of PSU **1** and generated complex mixtures (Supplementary Fig. S35). The reaction in 1,4-dioxane and xylene afforded yellow suspensions and lower yields of **9** (69% and negligible yields, respectively), suggesting that the poor solubility of the reactants and the base affected negatively the reactivity. This decrease in reactivity was probably increased in the case of resins (Table 1, Entries 10 and 11). Conversely, suitable amide solvents with high polarity could solvate the reactants to enhance the reactivity, thereby promoting the degradation.

### Synthesis of photoemissive PEEK powder

**Fig. 4:**
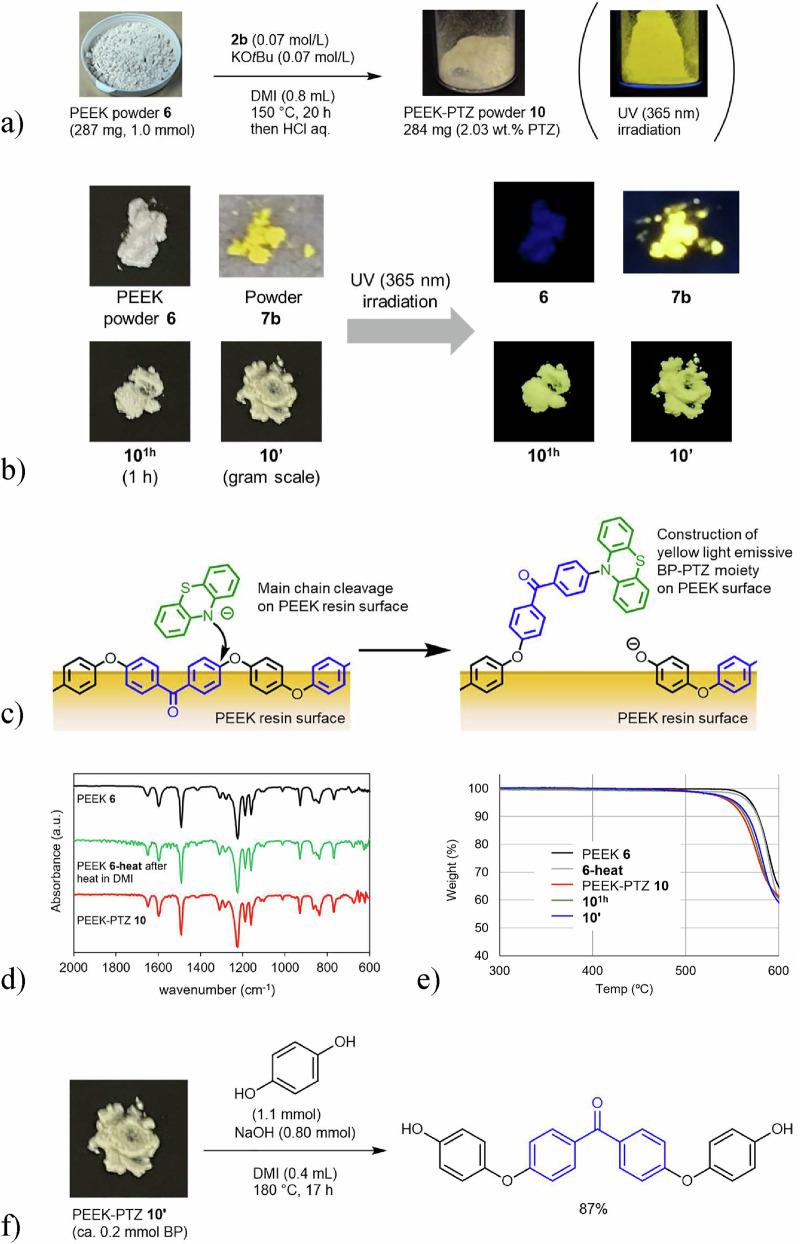
Degradative functionalization of the PEEK powder surface with PTZ. **a** Reaction and pictures of PEEK powder **6** to produce PEEK-PTZ powder **10**. **b** Observation of PEEK powders **6,** **7b,** **10**^*1h*^, and **10’** under 365 nm UV irradiation. **c** Surface degradation pathway to form the surface BP-PTZ moiety. **d** ATR-FTIR spectra of PEEK **6** (black), PEEK **6-heat** treated in DMI at 150 °C for 24 h (green), and PEEK-PTZ powder **10** (red). Area ratios of intensities of the peaks between 1310 cm^-1^ derived from crystalline moiety and 1280 cm^-1^ derived from crystalline and amorphous moieties, **6**: 0.75, **6-heat**: 1.86, **10**: 1.86. **e** TGA curves of PEEK **6** (black), **6-heat,** **10** (red), **10**^1*h*^ (green), and **10’** (blue) measured at 10  °C min^−1^ in nitrogen atmosphere. **f** Depolymerization of **10’** with hydroquinone and NaOH in DMI at 180 °C for 17 h to provide bis(4-(4-hydroxyphenoxy)phenyl)methanone, which was analyzed by ^1^H NMR spectroscopy.

As mentioned above, PEEK is insoluble in almost all solvents even at high temperature; therefore, the degradation process starts on the PEEK surface^23^. If the main chains could be cleaved while maintaining insolubility and stability without excessively degrading the PEEK resin on the surface, it would be possible to functionalize the PEEK surface. Therefore, we focused on the low reactivity of **2b** toward the degradation of PEEK to obtain photoemissive PEEK materials. Based on the above verification of the degradation reactivity, the reaction of PEEK powder **6** (287 mg, 1.0 mmol relative to the molecular weight of the monomer) was conducted under the conditions at lower concentration to suppress the reactivity, 0.07 mol/L (0.06 equiv.) of **2b**, and KO*t*Bu in DMI (0.8 mL) at 150 °C for 20 h. After treating with HCl, functionalized PEEK-PTZ powder **10** (266 mg) was successfully obtained, which exhibited yellow fluorescence under 365 nm UV irradiation (Fig. 4a). This emissive color was clearly different from that of the parent PEEK powder **6**, which emitted pale blue fluorescence (Fig. 4b). The fact that yellow luminescence was observed from **7b** demonstrates that the yellow emission from **10** is attributed to the constructed *N*-benzophenone-PTZ (BP-PTZ) moiety as the D-A unit bond to the rest of the PEEK chain on the surface by the main-chain cleavage using **2b** (Fig. 4c). ATR-FTIR spectroscopic results showed that almost no differences were observed between the IR spectra of PEEK-PTZ **10** and PEEK powder **6**, with the signals derived from PTZ being hardly observed (Fig. 4d and Supplementary Fig. S9). This may be due to the small amount of PTZ introduced and indicates that this functionalization does not change the PEEK structure. The intensity of the characteristic peak of crystalline PEEK at 1310 cm^−1^^40^ increased compared with that of neat PEEK powder **6** and was similar to that of PEEK powder **6-heat** after treatment in DMI at 150 °C for 24 h (Supplementary Table S4)^41–43^. A thermogravimetric analysis (TGA) showed that the thermal durability of **10** slightly decreased compared with that of **6** and **6-heat**, maybe due to a decrease in molecular weight but maintains the robustness as the super engineering plastics (Fig. 4e and Supplementary Fig. S10). After heating at 320 °C (a higher temperature than the melting point of **7b**, 307 °C)^35^ for 3 h, the powder emitted yellow light under 365 nm UV irradiation (Supplementary Fig. S11). To confirm the reproducibility, we performed the reaction several times (Supplementary Table S1), yielding the luminescent PEEK-PTZ powder in all cases. Short reaction times such as 1 h also provided yellow emissive powder **10**^1h^ with the same thermal stability as **10**, suggesting that the transformation was complete after 1 h and the reactivity depended on the concentration of reactants. To evaluate the scalability of the functionalization, we performed a gram-scale reaction of PEEK powder **6** (2.80 g) with **2b** under the same conditions described in Fig. 4a, which afforded 98 weight % (wt.%) PEEK-PTZ powder **10’** (Fig. 4b, see section 4-1 in Supplementary Methods). Yellow emissive powder **10**^**DMAc**^ was also obtained using DMAc solvent, indicating that PEEK-PTZ units were at least generated on the surface, which was supported by an evolved gas analysis (EGA) with time-of-flight mass spectrometry (EGA-TOFMS) analysis (Supplementary Table S1 and Figs. S8, S12). The measurements of the particle-size distribution revealed that the powder size tends to decrease slightly as a result of the degradative functionalization whereas scanning electron microscopy (SEM) showed no visible differences between **6** and **10’** (Supplementary Figs. S14 and S15). Based on the analysis that PEEK-PTZ powder was almost composed of PEEK, the depolymerization of **10’** with hydroquinone with NaOH in DMI at 180 °C for 17 h was examined to give the monomer, bis(4-(4-hydroxyphenoxy)phenyl)methanone, in high yield (87%) with small amounts of phenothiazine-derived products (Fig. 4f and supplementary Fig. S16)^27^. In addition, the degradation using 2-ethylhexanethiol and NaOH at 100 °C also provided the dithiofunctionalized benzophenone and hydroquinone in 81% and 80% yields, respectively (Supplementary Fig. S17)^23,25^.

**Fig. 5:**
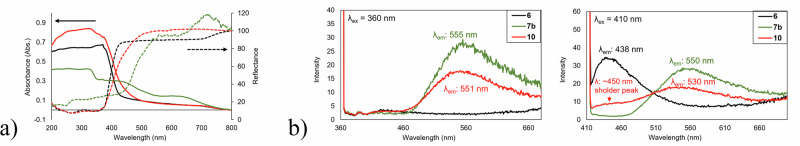
Absorption, reflection, and photoluminescence spectra. **a** Absorption (bold) and reflection (dash) spectra of **6** (black), **7b** (green), and **10** (red). **b** Photoluminescence spectra of **6** (black), **7b** (green), and **10** (red) upon irradiation with 360 and 410 nm light.

To investigate the yellow emission from PEEK-PTZ powder **10**, we analyzed its photophysical properties together with those of PEEK powder **6** and **7b** using a calibrated integrating sphere system. The absorption and reflection spectra of **10** showed that the longest absorption wavelength was shifted to the longer wavelength region (ca. 500 nm) compared with that of PEEK **6** (ca. 440 nm) (Fig. 5a and Supplementary Fig. S18), which is presumably attributed to the effect of the terminal BP-PTZ moiety. The photoluminescence (PL) spectrum of **10** exhibited an emission maximum at around 550 nm, which was almost the same as that of **7b**, upon irradiation with 360 nm light (Fig. 5b and Supplementary Fig. S19). Conversely, no PL was observed for PEEK powder **6**. Meanwhile, when irradiated with 410 nm light, the PL spectrum of **10** exhibited an emission maximum at 530 nm and a weak shoulder at around 455 nm originating from the BP-PTZ moiety and the PEEK chain, respectively. The overall quantum fluorescence efficiency (*Φ*_F_) values of **10** under irradiation with 360 and 410 nm light were 4% and 6%, respectively (Supplementary Table S4), which were comparable to those of **7b** under the same analytical conditions. These results demonstrate that **10** combines the characteristics of the original PEEK and the introduced BP-PTZ moiety. PEEK polymers with photoluminescent properties were previously reported; however, they were prepared using luminescent monomers^44,45^. In contrast, the present method enables the synthesis of luminescent PEEK by directly functionalizing the PEEK resin itself.

**Fig. 6:**
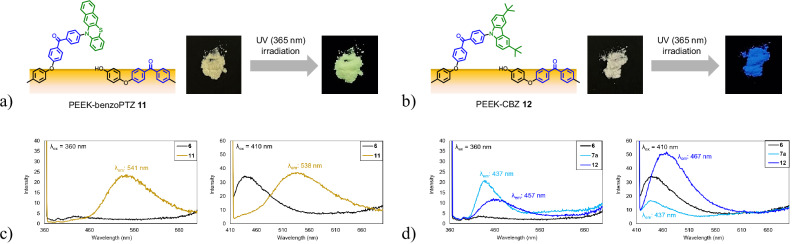
Photophysical properties of functionalized PEEK powder. **a** PEEK-benzoPTZ **11** derived from 12*H*-benzo[*b*]phenothiazine. **b** PEEK-CBZ **12** derived from 3,6-di-*tert*-butyl carbazole. Photoluminescence spectra of **c 11** (yellow) and **d 7a** (pale blue) and **12** (blue) compared with that of **6** (black) obtained upon irradiation with 360 and 410 nm light.

Other nucleophiles were applicable to this functionalization. Using 12*H*-benzo[*b*]phenothiazine (**2 d**) under the same conditions as those described in Fig. 4a provided PEEK-benzoPTZ powder **11**, which emitted yellow light under UV light irradiation at 365 nm (Fig. 6a and Supplementary Page S15 and Fig. S18 and S20). The PL spectra of **11** under 360 and 410 nm irradiation mainly showed the characteristics of the BP-benzoPTZ moiety, and the *Φ*_F_ values were determined to be 3% and 6%, respectively. In addition, the reaction between PEEK powder **6** and **2a** using cesium carbonate as the base afforded blue light–emissive PEEK-CBZ powder **12** (Fig. 6b, Supplementary Page S16 and Figs. S18 and S21). Considering that **7a** emits blue light under 365 nm irradiation indicates that the photophysical property of **12** is attributed to the surface BP-CBZ moiety. The PL spectra supported this phenomenon. These results demonstrate that various nucleophiles and bases can be applied to degradative functionalization to provide the corresponding PEEK materials.

### Application of photoemissive PEEK powder as photoredox catalyst

**Fig. 7:**
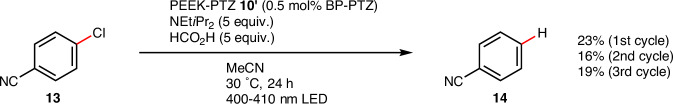
Reusability of PEEK-PTZ powder 10’ in three runs of dehalogenation of 13. Reaction condition: **13**, PEEK-PTZ (0.5 mol% PTZ unit), ethyldiisopropylamine (5 equiv.), formic acid (5 equiv.), and acetonitrile (1.0 mL) at 30 °C for 24 h under UV irradiation using LED light purchased from Aldrich (catalog No. ALDRP2, 400–410 nm) at a distance of 0.1 cm. After the reaction, **10’** was recovered by the filtration followed by washing with acetone. Et ethyl, *i*Pr isopropyl.

Having confirmed the emissive PEEK-PTZ units on the surface, we investigated its applicability as a photoredox catalyst according to the previous polymer-supported photoredox catalysts including *N*-aryl-phenothiazine^46–54^. As a reaction model, we selected the dehalogenation of haloarenes using photoredox organocatalyst *N*-Ph-PTZ reported by Hawker and Alaniz^52,53^. Based on the preliminary experiments using 4-bromobenzonitrile (Supplementary Table S2 and Fig. S22), we carefully set up the reaction conditions employing 4-chlorobenzonitrile (**13**), a photocatalyst, ethyldiisopropylamine, and formic acid in acetonitrile at 30 °C for 72 h under 400–410 nm irradiation from LED light (Table 2 and supplementary Fig. S23). Under these conditions, the dehydrogenation of **13** hardly proceeded in the absence of catalysts (Table 2, Entries 1 and 2), whereas benzonitrile (**14**) was observed when using 5 mol% of the standard catalyst 10-phenylphenothiazine (Table 2, Entry 3). Meanwhile, using about 5 mg of **10** containing ca. 0.5 mol% of BP-PTZ afforded **14** in 90% yield (Average of four independent experiments. See supplementary Table S3) by bringing the LED source and solution close together (Table 2, Entry 4). The yield of **14** decreased to 67% under 1 mml scale of **13** probably due to an increase in the volume of the solution (Table 2, Entry 6 and supplementally Fig. S24). In fact, this yield was similar to that obtained using *N*-Ph-PTZ under same conditions (Table 2, Entry 5). The PEEK-PTZ powder showed reusability for this catalytic dehalogenation reaction (Fig. 7). These results at least suggested that the degradative surface functionalization of stable super engineering plastics like PEEK can be utilized as a method for supporting catalysts.

**Table 2:** Catalytic dehalogenation of 4-chlorobenzonitrile


Entry	13 (mmol)	catalyst	14 (%)
1^a^	0.1	—	2
2^a^	0.1	PEEK powder **6** (4.9 mg)	3
3^a^	0.1	10-Phenylphenothiazine (5 mol%)	89
4^a^	0.1	PEEK-PTZ **10** (4.7 mg, 0.5 mol% BP-PTZ)	90^b^
5^c^	1.0	10-Phenylphenothiazine (0.5 mol%)	67
6^c^	1.0	PEEK-PTZ **10’** (50.9 mg, 0.5 mol% BP-PTZ)	67

^a^A mixture of **13** (0.1 mmol), catalyst, NEt*i*Pr_2_ (0.5 mmol), HCO_2_H (0.5 mmol), and acetonitrile (1.0 mL) was stirred at 30 °C under UV irradiation using LED light purchased from Aldrich (catalog No. ALDRP2, 400–410 nm) at a distance of 0.1 cm as shown picture instead of Entry 3 (1.5–2.0 cm). Yields were determined by GC and ^1^H NMR spectroscopy. ^b^Average of four independent experiments (87%, 89%, 91%, and 92%). ^c^A mixture of **13** (1.0 mmol), catalyst, NEt*i*Pr_2_ (5 mmol), HCO_2_H (5 mmol), and acetonitrile (10.0 mL) was stirred at 30 °C under UV irradiation using LED light purchased from Aldrich (catalog No. ALDRP2, 400–410 nm) at a distance of 0.1 cm.

### Degradative functionalization of PEEK plates

**Fig. 8:**
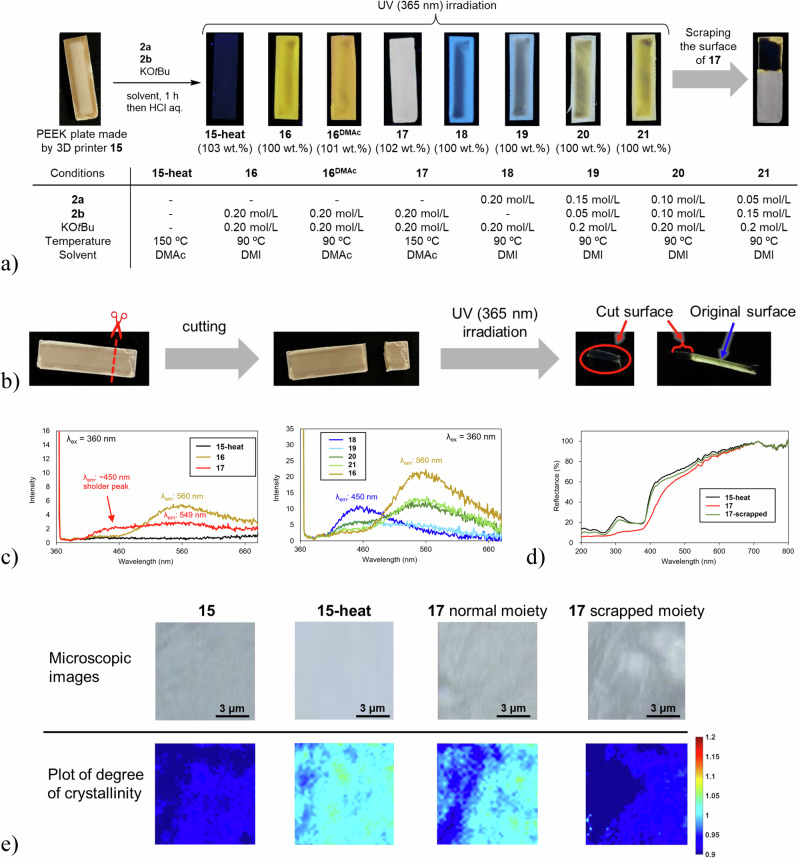
Degradative surface functionalization of PEEK plates. **a** Treatment of PEEK plate **15** fabricated using a 3D printer with various amounts of **2a,** **2b**, and KO*t*Bu in DMI or DMAc solvent to provide yellow, white, and blue light–emissive PEEK plates **16**–**21**. For comparison, PEEK plate **15** was heated in DMAc at 150 °C for 1 h to provide plate **15-heat**. Obtained plates were irradiated with 365 nm light. Scrapped surface of **17** did not emit, and the generated edge part emitted a yellow light. **b** The plate **16** was cut and the generated cut surface did not emit. **c** Photoluminescence spectra of **15-heat** (black), **16** (yellow), **17** (red), **18** (blue), **19** (pale blue), **20** (green), and **21** (pale green) upon irradiation with 360 nm light. **d** Reflection spectra of **15-heat** (black), **17** (red), and the scrapped part of **17** (green). **e** Microscopic images and plots of degrees of crystallinity of **15,** **15-heat**, and **17** (blue and red colors represent the lowest and highest degree of crystallinity, respectively, as determined by the area ratio of the peaks at 1310 and 1280 cm^−1^).

The degradative surface functionalization was applicable to PEEK pellets and plates larger in volume than powder, which require gentle conditions to preserve the surface structure. Based on preliminary experiments using purchased PEEK pellets and plates (Supplementary Figs. S25–32), pure PEEK plate **15** fabricated using a 3D printer^55,56^ was used in the surface reaction with 0.20 mol/L of **2b** and KO*t*Bu in DMI or DMAc at 90 °C for 1 h. As a result, PEEK-PTZ plates **16** and **16**^**DMAc**^ were obtained without changing the shape, which emitted yellow light upon irradiation with 365 nm light (Fig. 8a and Supplementary Fig. S34). Although PEEK powder **6** hardly underwent the degradation with **2b** at 90 °C for 20 h (Supplementary Fig. S33), a few amount of PEEK-PTZ units were probably constructed on the surface. In fact, only a small amount of **2b** reacted with PEEK plate (Supplementary Figs. S25c, S34b). On the other hand, treatment in DMAc at 150 °C for 1 h provided white light–emissive plate **17**, which was maybe derived from the co-generation of blue-light emissive byproduct units on the surface (Supplementary Fig. S35). In fact, the yellow emission of **16**^**DMAc**^ was not changed after further heating in DMAc at 150 °C for 1 h. PL spectra of **16** showed only a signal around 560 nm similar to **7b** and **10** under 360 nm light (Fig. 8c left and Supplementary Fig. S40a), whereas a shoulder peak at around 450 nm probably derived from the byproducts was detected from **17** (Supplementary Fig. S40b). After the surface of **17** was scrapped, the white light disappeared except at the edges, which emitted yellow light under 365 nm light irradiation. Similarly, the cut surface of **16** did not emit (Fig. 8b and supplementary Fig. S25c, S31, S34b). These observations confirmed that the degradative functionalization proceeded on its surface and the interior was not involved. In this relation, reflection spectrum of **15-heat**, which was obtained upon heating of **15** at 150 °C for 1 h in DMAc, was similar to that of the scrapped surface of **17**, and was different from the original surface of **17** (Fig. 8b and Supplementary Fig. S39). This method could be performed even at room temperature and using other organic solvents such as xylene and di(ethoxyethyl)ether, but water was not effective because the reactants were not soluble (Supplementary Figs. S36, S37, S38). These results suggest that surface functionalization of PEEK resin proceeds when a small amount of reactant is dissolved, even though the used organic solvent is unsuitable for the degradation.

This functionalization using 0.2 mol/L of **2a** in DMI at 90 °C for 1 h provided blue light–emissive plate **18** (Fig. 8a and Supplementary Page S44), whose PL spectrum under 360 nm photoirradiation showed a maximum emission wavelength at 450 nm from the *N*-BP-CBZ group (Fig. 8c right and Supplementary Figs. S40c). When a mixture of **2a** and **2b** was used, the emissive color from the produced **19,** **20**, and **21** changed from blue to yellow in accordance with the mixing ratio, showing that the dual functionalization proceeded. The PL spectrum supported this change. As the usage ratio of **2a** decreased, the signal around 450 nm decreased and the signal around 560 nm increased.

Plates **15,** **15-heat**, and **17** were analyzed via FTIR microscopy (Fig. 8e and Supplementary Fig. S42 and Table S4). The microscopic images show that the process did not damage the surface, which were further supported by the SEM images (Supplementary Fig. S43). The crystallinity of the plates was roughly determined by comparing the intensities of the peaks at 1310 and 1280 cm^−1^ in the IR spectra. The crystallinity of the original surface of **17** was higher than that of **15** and similar to that of **15-heat**. The scrapped part of **17** showed the same crystallinity as **15**. ATR-FTIR spectroscopic analysis also showed the same trend (Supplementary Fig. S41). These results also indicate that the low crystallinity is conducive to the degradative surface functionalization because it favors the penetration of the reactants into the resin^29^.

## Conclusions

We developed a main chain cleaving degradation methodology for polymers, which can be applied to the production of not only monomeric organics such as D-A-D type molecules but also functionalized polymers by constructing surface moieties. The later protocol is suitable for robust polymers such as PEEK that are insoluble and remain stable even after a slight reduction in molecular weight. Using electron-rich azacycles for the functionalization of PEEK powder, pellets, and plates, light–emissive PEEK materials were obtained. Furthermore, the prepared surface-functionalized PEEK powder underwent the depolymerization to provide the corresponding monomer. In the case of PEEK plates, the emission color could be controlled by a kind of functional group or by changing the reaction temperature. In addition, an *N*-aryl-phenothiazine moiety introduced on the PEEK surface could be used as a photoredox catalyst. The present degradation–functionalization method can be expected to provide a platform for the “Repurpose” of resin materials.

## Methods

### General procedure for the degradation of PSU pellets 1 using 3,6-di-*tert*-butylcarbazole (2a) with KO*t*Bu

To a mixture of PSU pellets **1** (87.2 mg, 0.197 mmol relative to the molecular weight of the monomer) and KO*t*Bu (53.9 mg, 0.48 mmol) were added DMI (0.4 mL) and **2a** (142 mg, 0.51 mmol) in a 3.0 mL vial under argon atmosphere. The resultant mixture was stirred at 120 °C for 5 h and then cooled to room temperature. HCl aq. (1 M, 3 mL) and chloroform (0.8 mL) were added to quench the reaction. The organic component was extracted and concentrated in vacuo to give a crude product, which was purified via thin-layer chromatography (hexane–CHCl_3_, 3:1) to afford **3a** (138 mg, 0.179 mmol) and **4** (40.5 mg, 0.177 mmol) in 90% yield each.

### Degradative surface functionalization of PEEK powder 6 using 10*H*-phenothiazine (2b) and KO*t*Bu

To a mixture of PEEK powder **6** (2.80 g, 9.69 mmol of PEEK relative to the molecular weight of the monomer), **2b** (106 mg, 0.53 mmol), and KO*t*Bu (54.5 mg, 0.49 mmol) was added DMI (8.0 mL) in a 20 mL vial under argon atmosphere. The resultant mixture was stirred at 150 °C for 20 h. After cooling to room temperature, HCl aq. (2 M, 2.0 mL) was added to quench the reaction. The resulting yellow solid was separated from the colorless solution via filtration and washed with water, methanol, and acetone. The obtained yellow solid was dried in vacuo to obtain yellow powder **10’** (2.75 g, 98 wt.% based on used PEEK powder).

### Degradative surface functionalization of PEEK plates using 2b and KO*t*Bu in DMI solvent

PEEK plate **15** with dimensions of ca. 10 mm × 30 mm × 2 mm (593 mg), which was fabricated using a 3D printer, was placed in a 12 mL vial, and **2b** (239 mg, 1.20 mmol), KO*t*Bu (135 mg, 1.20 mmol), DMI (6.0 mL), and a magnetic stirring bar were added under argon atmosphere. The mixture was stirred at 90 °C for 1 h and then cooled to room temperature. The red solution was removed by decantation, and the plate was treated with water and HCl aq. (2 M, 1.0 mL) and then washed with water, methanol, and acetone. The obtained plate was dried at 115 °C for 1 h to obtain PEEK-PTZ plate **16** (594 mg, 100 wt.% based on the used PEEK plate).

## Supplementary information

Transparent Peer Review file

Supplementary Information

Description of Additional Supplementary Files

Supplementary Data 1

Supplementary Data 2

## Data Availability

The data obtained in this study are available within this article and its supplementary information and are also from the corresponding authors upon reasonable request. Original ^1^H and ^13^C spectra of the compounds obtained in this manuscript are available in Supplementary Data 1. Source data for FTIR, TGA curve, and absorption, reflection, and photoluminescence spectra in the manuscript are available in Supplementary Data 2.
