# Supplementary material for: Amination–degradation of super engineering plastics for the construction of surface emissive resin materials
Source: Commun Chem. 2026 Apr 30;9:233. doi: 10.1038/s42004-026-02051-1 (PMC13338230; doi:10.1038/s42004-026-02051-1)
Supplement: Supplementary file 2 — Supplementary Information [file 42004_2026_2051_MOESM2_ESM.pdf]

Supplementary Information

Amination–Degradation of Super Engineering Plastics for the Construction of Surface Emissive Resin Materials

Yasunori Minami,[1,2,3,4] Shunsuke Tsuyuki,[4] Ryota Watanabe,[5] Nobuyasu Itoh,[6] Masaru Yoshida[7]

[1] Integrated Research Center for Circular Technology, National Institute of Advanced Industrial Science and Technology (AIST), Tsukuba Central 5, 1-1-1 Higashi, Tsukuba, Ibaraki 305-8565, Japan.

[2] Graduate School of Pure and Applied Science Department, University of Tsukuba, 1-1-1 Tennoudai, Tsukuba, Ibaraki 305-8577, Japan.

[3] PRESTO, Japan Science and Technology Agency (JST), 1-1-1 Higashi, Tsukuba, Ibaraki 305-8565, Japan

[4] Institute for Chemical Process Technology, National Institute of Advanced Industrial Science and Technology (AIST), Tsukuba Central 5, 1-1-1 Higashi, Tsukuba, Ibaraki 305-8565, Japan.

[5] Research Institute for Sustainable Chemistry, National Institute of Advanced Industrial Science and Technology (AIST), Tsukuba Central 5, 1-1-1 Higashi, Tsukuba, Ibaraki 305-8565, Japan.

[6] National Metrology Institute of Japan, National Institute of Advanced Industrial Science and Technology (AIST), Tsukuba Central 3, 1-1-1 Umezono, Tsukuba, Ibaraki 305-8563, Japan.

[7] Catalytic Chemistry Research Institute, National Institute of Advanced Industrial Science and Technology (AIST), Tsukuba Central 5, 1-1-1 Higashi, Tsukuba, Ibaraki 305-8565, Japan.

email: yasu-minami@aist.go.jp

Table of Contents

Supplementary Methods

1. General information S3
2. Chemicals S4
3. Amination-degradation to form diaminated products S5
   3-1. Experimental procedures for the degradation to form diaminated products S5
   3-2. Spectrum data on the products S9
      Fig. S1 | NMR spectra of used PSU S11
      Fig. S2 | The reaction mixture between PSU and **2a** S12
      Fig. S3 | Reaction of PSU pellets with 4-decylamiline S12
4. Degradative surface functionalization of PEEK powder S13
   4-1. Experimental procedures (**Fig. S4-S7**) S13
      Table S1 | Summary of the degradation of PEEK surface (**Fig. S8**) S17
   4-2. Analyses of used PEEK and surface-functionalized PEEK powder S18
      Fig. S9 | ATR-FTIR spectroscopic analyses S18
      Fig. S10 | Thermogravimetric analysis and differential thermal analysis S19
      Fig. S11 | Heat test of PEEK-PTZ powder **10** at 320 °C for 3 h S20
      Fig. S12 | Thermogravimetric/high resolution mass spectrometric analysis of $\mathbf{10^{DMAc}}$ S21
      Fig. S13 | EGA-TOFMS Analyses of **12** S21
      Fig. S14 | Results of particle size distribution S22-23
      Fig. S15 | SEM images of PEEK powder **6** and PEEK-PTZ powder **10'** S24
      Fig. S16-S17 | Degradation of **10'** S25-26
      Fig. S18 | Absorption and reflectance spectra S27
      Fig. S19-S21 | Photoluminescence spectra of **6**, **10**, **11**, and **12** S28-29
5. Catalytic reduction of 4-halobenzonitrile to benzonitrile S30
   5-1. Procedure (**Fig. S22**) S30
      Table S2 | Catalytic hydrogenolysis of 4-bromobenzonitrile (**13'**) S31
      Table S3 | Catalytic hydrogenolysis of 4-chlorobenzonitrile (**13**) (**Fig. S23**) S32
      Fig. S24 | Dehalogenation of 1.0 mmol scale of **13** using catalyst **10'** S33
6. Preliminary surface functionalization of purchased PEEK pellets (**Fig. S25**) S34
7. Preliminary surface functionalization of purchased PEEK plates (**Fig. S26-S32**) S35
8. Degradative surface functionalization of PEEK plates made of 3D printer S40
   7-1. Procedures (**Fig. S33-S37**) S40
   7-2. Examination of solvents and temperature (**Fig. S38**) S45
   7-3. Analyses of functionalized PEEK lumps (**Fig. S39-S43**) S46

Table S4 | Emission maxima (λ_{em}) and absolute internal quantum efficiencies (Φ_f) S50

Supplementary references S51

Supplementary Methods

1. General.

All manipulations of oxygen- and moisture-sensitive materials were conducted in a dry box under an argon atmosphere. Analytical TLC was performed on Merck Kieselgel 60 F254 (0.25 mm) plates. Visualization was accomplished with UV light (254 nm). ^{1}H and ^{13}C{^{1}H} NMR spectra in $CDCl_3$ solution were recorded with Bruker AVANCE III HD 600 spectrometer. The ^{1}H NMR (600 MHz) and ^{13}C{^{1}H} NMR (151 MHz) chemical shifts were reported in δ (ppm). ^{1}H NMR and ^{13}C{^{1}H} NMR spectra were referenced to the residual solvent signals or tetramethylsilane. ^{1}H NMR data are reported as follows: chemical shift, multiplicity (s = singlet, d = doublet, t = triplet, q = quartet, quint = quintet, sext = sextet, sept = septet, br = broad, m = multiplet), coupling constants (Hz), and integration. Melting points were measured by a MPA100 Optimelt Automated Melting Point System. The IR spectra were measured by Bruker ALPHA II equipped with eco-ATR. TG analysis was performed by DTG-60 under nitrogen atmosphere. High-resolution mass spectra (HRMS) were measured on Bruker compact mass spectrometer under positive electrospray ionization (ESI^+) or negative electrospray ionization (ESI^-) conditions. Particle size distribution was measured by MICROTRACBELL MT3000II. SEM was measured by JEOL JCM-7000. High-temperature GPC analysis was measured on Tosoh HLC-8321GPC/HT with TSKgel GMH_{HR}-H (S) HT2 column. Absorption and reflection spectra at a powder form were measured on SHIMADZU UV-2600i equipped with ISR-2600Plus. Fluorescence spectra and absolute quantum yields at solid state were recorded with SHIMADZU RF-6000 equipped with an integrating sphere unit. 3D-printer F160-PEEK made of CreatBot was used for the preparation of PEEK plates. Microscopic FT-IR analysis was performed on Bruker Hyperion3000 equipped with FPA. Microscopic Raman spectroscopic analysis was performed with Horiba LabRAM HR Evolution.

The evolved gas analysis (EGA) with time-of-flight mass spectrometry (EGA-TOFMS) system comprised a thermogravimeter (STA 2500 Regulus; NETZSCH, Germany), gas chromatograph (7890 B; Agilent Technologies), and time-of-flight mass spectrometer (JMST2000, JEOL, Japan) equipped with an in-line EGA accessory (NETZSCH, Germany). Approximately 1 mg of the sample was used for EGA-TOFMS measurements. The sample was placed in an aluminum pan and heated from 300 to 550 °C at a heating rate of 10 °C min^{-1} under a helium atmosphere. A portion of the gas flow (70 mL/min) was continuously introduced into the mass spectrometer through a deactivated fused silica column (10 m × 0.32 mm i.d., Agilent Technologies, USA), which was heated to 280 °C to prevent the condensation of less volatile products in the capillary. Mass spectral measurements were performed using **FI** in the mass range of *m/z* 40–800 with a recording interval of 1 s. The mass spectrometer was tuned using octamethylcyclotetrasiloxane, and the peak resolution was adjusted to approximately 20,000 for *m/z* 281.05114.

2. Chemicals.

All reactions were carried out under inert atmosphere such as nitrogen and argon. Unless otherwise noted, commercially available reagents were used without further purification.

Polysulfone (PSU) (pellet (Transparent), average M_w ~35,000 by LS, average M_n ~16,000 by MO as the catalog spec, Cat. No. 428302), poly(1,4-phenylene ether ether sulfone) (PEES) (Pellet, Cat. No. 440965), polyetheretherketone (PEEK) (powder: mean particle size 80micron, Cat. No. GF75065755. Pellet: average M_w ~20800, average M_n ~10300, Cat. No. 456640.), and potassium *tert*-butoxide (Cat. No. 156671) were purchased from Sigma–Aldrich Japan. Sodium hydroxide (Cat. No. 194-18865), potassium hydroxide (Cat. No. 165-21825), dehydrated *N*,*N*-dimethylacetamide (Cat. No. 042-32353), and xylene (Cat. No. 240-00865) were purchased from FUJIFILM Wako Chemicals. 1,3-Dimethyl-2-imidazolidinone (Cat. No. 11208-00) was purchased from Kanto Chemicals. 3,6-Di-*tert*-butylcarbazole (Cat. No. D3952), pyrrole (Cat. No. P0574), 10*H*-phenothiazine (Cat. No. P0106), 12*H*-benzo[b]phenothiazine (Cat. No. B6562), and 4-phenoxybenzophenone (Cat. No. P1328) was purchased from TCI. PEEK filament was purchased from kexcelled (Cat. No. K10-1.75-NAT-1KG). PEEK plate was purchased from Asone (Cat. 2-9239-01).

3. Amination-degradation to form diaminated products

3-1. Experimental procedures for the degradation to form diaminated products

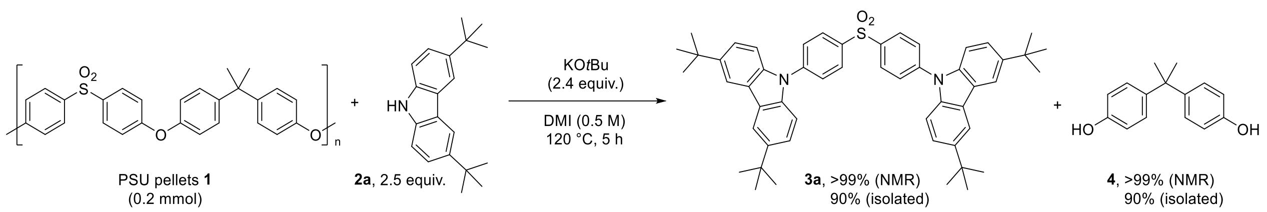

Degradation of PSU pellets 1 by 3,6-di-*tert*-butylcarbazole (2a) with potassium *tert*-butoxide.
To a mixture of PSU pellets **1** (87.2 mg, 0.197 mmol relative to the molecular weight of the monomer) and potassium *tert*-butoxide (53.9 mg, 0.48 mmol) were added 1,3-dimethyl-2-imidazolidinone (DMI, 0.4 mL) and 3,6-di-*tert*-butyl-carbazole (**2a**) (142 mg, 0.51 mmol) in a 3.0 mL vial under argon atmosphere. The resultant mixture was stirred at 120 °C for 5 h and then cooled to room temperature. HCl aq. (1 M, 3 mL) and chloroform (0.8 mL) were added to quench the reaction. The organic component was extracted and concentrated in vacuo to give a crude product, which was analyzed by ^{1}H NMR spectroscopy to determine the yield of the products. The crude product was purified via thin-layer chromatography (hexane–$CHCl_3$, 3:1) to afford **3a** (138 mg, 0.179 mmol) and **4** (40.5 mg, 0.177 mmol) in 90% yield each.

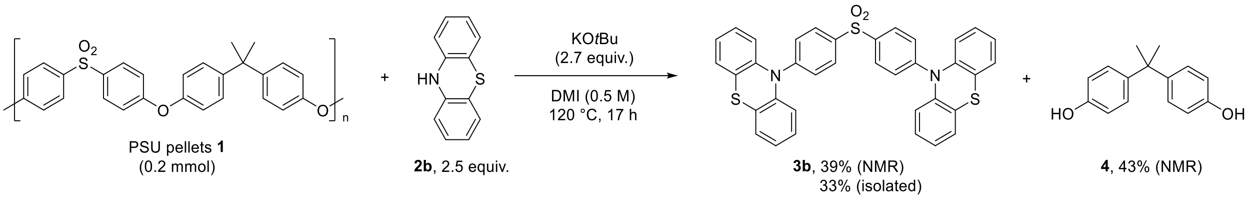

Degradation of PSU pellets 1 by 10*H*-phenothiazine (2b) with potassium *tert*-butoxide.
To a mixture of PSU **1** pellets (87.8 mg, 0.198 mmol relative to the molecular weight of the monomer) and potassium *tert*-butoxide (60.8 mg, 0.54 mmol) were added 1,3-dimethyl-2-imidazolidinone (DMI, 0.4 mL) and 10*H*-phenothiazine (**2b**) (100 mg, 0.50 mmol) and in a 3.0 mL vial under argon atmosphere. The resultant mixture was stirred at 120 °C for 17 h and then cooled to room temperature. HCl aq. (1 M, 3 mL) and chloroform (0.8 mL) were added to quench the reaction. The organic component was extracted and concentrated *in vacuo* to give crude product which was analyzed by ^{1}H NMR spectroscopy to determine the yield of the products. The crude product was purified by recrystallization using hot ethanol to afford **3b** (40 mg, 0.0653 mmol) in 33% yield.

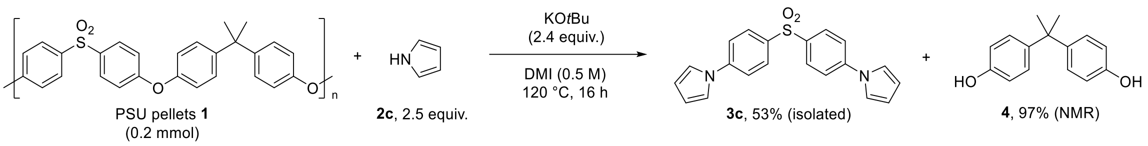

Degradation of PSU pellets 1 by pyrrole (2c) with potassium *tert*-butoxide.

To a mixture of PSU **1** pellets (87.3 mg, 0.197 mmol relative to the molecular weight of the monomer) and potassium *tert*-butoxide (54.7 mg, 0.49 mmol) were added 1,3-dimethyl-2-imidazolidinone (DMI, 0.4 mL) and pyrrole (**2c**) (33 mg, 0.49 mmol) and in a 3.0 mL vial under argon atmosphere. The resultant mixture was stirred at 120 °C for 16 h and then cooled to room temperature. HCl aq. (1 M, 3 mL) was added to quench the reaction. The organic component including precipitates was extracted with chloroform and ethyl acetate. The precipitate was filtered out and dried under vacuum to obtain **3c** (36.4 mg, 0.10 mmol) in 53% yield. The filtrate was concentrated and then analyzed by ^{1}H NMR spectroscopy to determine the yield of **4** (97%).

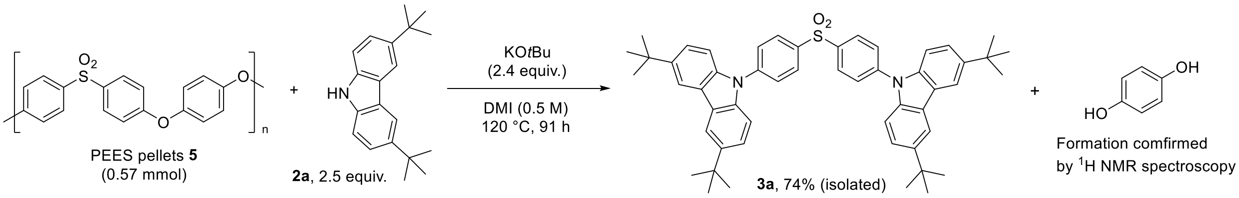

Degradation of PEES pellets 5 by 3,6-di-*tert*-butylcarbazole (2a) with potassium *tert*-butoxide.

To a mixture of PEES **5** pellets (183 mg, 0.565 mmol relative to the molecular weight of the monomer) and potassium *tert*-butoxide (5153 mg, 1.36 mmol) were added 1,3-dimethyl-2-imidazolidinone (DMI, 1.1 mL) and 3,6-di-*tert*-butyl-carbazole (**2a**) (395 mg, 1.41 mmol) and in a 3.0 mL vial under argon atmosphere. The resultant mixture was stirred at 120 °C for 91 h and then cooled to room temperature. Water (2 mL) was added to the mixture and filtered to get a solid which was recovered with CH_2Cl_2 and ethanol and dried to obtain a crude yellow powder. The crude product was purified by recrystallization using hot ethanol to afford **3a** (322 mg, 0.417 mmol) in 74% yield. Furthermore, after treating the filtrate with aq. HCl, extracted organic layer using ethyl acetate was analyzed by ^{1}H NMR spectroscopy to detect hydroquinone.

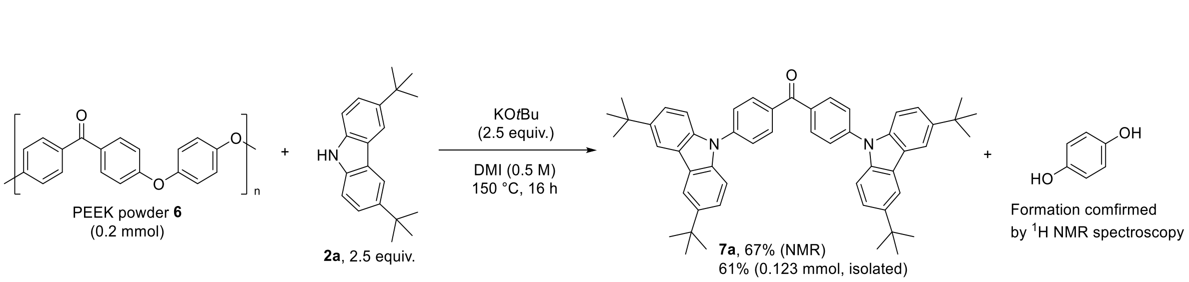

Degradation of PEEK powder 6 by 3,6-di-*tert*-butylcarbazole (2a) with potassium *tert*-butoxide.

To a mixture of PEEK powder **6** (57.7 mg, 0.200 mmol relative to the molecular weight of the

monomer) and potassium *tert*-butoxide (54.0 mg, 0.48 mmol) were added 1,3-dimethyl-2-imidazolidinone (DMI, 0.4 mL) and 3,6-di-*tert*-butyl-carbazole (**2a**) (140 mg, 0.50 mmol) and in a 3.0 mL vial under argon atmosphere. The resultant mixture was stirred at 150 °C for 16 h and then cooled to room temperature. Water (1.5 mL) was added to the mixture and filtered to get a solid which was dried to obtain a crude yellow powder. This crude solid was analyzed by ^{1}H NMR spectroscopy to determine the yield of **7a**. The crude product was purified by silica-gel chromatography using hexane/EtOAc (4:1) as eluents to afford **7a** (90.5 mg, 0.123 mmol) in 61% yield. Furthermore, after treating the filtrate with aq. HCl, extracted organic layer using ethyl acetate was analyzed by ^{1}H NMR spectroscopy to detect hydroquinone.

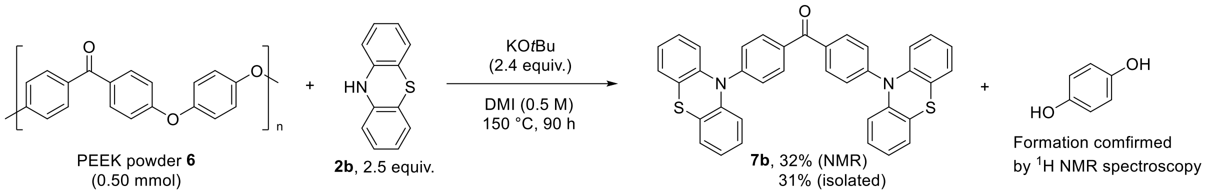

Degradation of PEEK powder 6 by 10*H*-phenothiazine (2b) with potassium *tert*-butoxide.

To a mixture of PEEK powder **6** (145 mg, 0.502 mmol relative to the molecular weight of the monomer) and potassium *tert*-butoxide (134 mg, 1.2 mmol) were added 1,3-dimethyl-2-imidazolidinone (DMI, 1.0 mL) and 10*H*-phenothiazine (**2b**) (250 mg, 1.25 mmol) and in a 3.0 mL vial under argon atmosphere. The resultant mixture was stirred at 150 °C for 90 h and then cooled to room temperature. Water (2 mL) was added to the mixture and filtered to get a solid which was dried to obtain crude powder. This crude solid was analyzed by ^{1}H NMR spectroscopy to determine the yield of **7b**. The crude product was purified by recrystallization using hot ethanol to afford **7b** (89.2 mg, 0.155 mmol) in 31% yield. Furthermore, after treating the filtrate with aq. HCl, extracted organic layer using ethyl acetate was analyzed by ^{1}H NMR spectroscopy to detect hydroquinone.

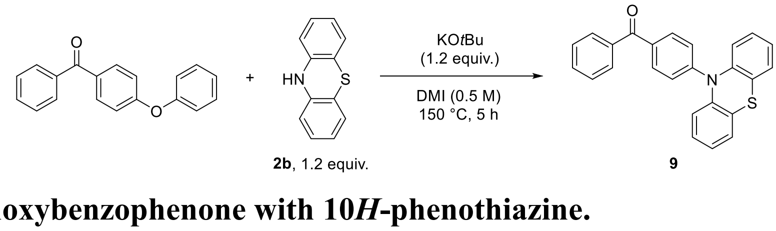

Reaction of 4-phenoxybenzophenone with 10*H*-phenothiazine.

To a mixture of 4-phenoxybenzophenone (**8**) (55.1 mg, 0.201 mmol) and potassium *tert*-butoxide (27.6 mg, 0.246 mmol) were added 1,3-dimethyl-2-imidazolidinone (DMI, 0.4 mL) and 10*H*-phenothiazine (**2b**) (48.3 mg, 0.242 mmol) and in a 3.0 mL vial under argon atmosphere. The resultant mixture was stirred at 150 °C for 5 h and then cooled to room temperature. 2 M HCl aq. (0.5 mL) and water were added to the mixture and reaction products were extracted with $CDCl_3$ which was analyzed by ^{1}H NMR spectroscopy to determine the yield of **9** (80%). After

the evacuation in vacuo, the crude product was purified by preparative thin-layer chromatography using CH_2Cl_2 and hexane/CH_2Cl_2 (1:2) as eluents to afford **9** (42.2 mg, 0.111 mmol) in 55% isolated yield.

3-2. Spectrum data on the products.

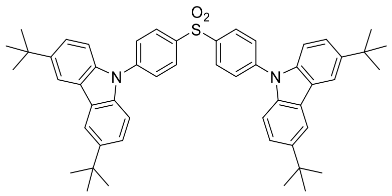

9,9'-(Sulfonylbis(4,1-phenylene))bis(3,6-di-*tert*-butyl-9*H*-carbazole) (3a).[S1] Known chemical (CAS registry number: 1396165-20-0). Colorless solid. ^{1}H NMR (600 MHz, $CDCl_3$) δ 1.46 (s, 36H, *t*Bu), 7.44 (d, *J* = 8.7 Hz, 4H, aromatic), 7.48 (dd, *J* = 1.9, 8.7 Hz, 4H, aromatic), 7.81 (AA'BB', 4H, aromatic), 8.13 (d, *J* = 1.6 Hz, 4H, aromatic), 8.24 (AA'BB', 4H, aromatic). ^{13}C NMR (151 MHz, $CDCl_3$) δ 32.0, 34.8, 109.2, 116.5, 124.0, 124.1, 126.6, 129.6, 138.3, 138.8, 143.2, 144.1.

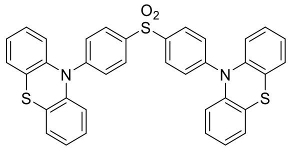

10,10'-(Sulfonylbis(4,1-phenylene))bis(10*H*-phenothiazine) (3b).[S2-S5] Known chemical (CAS registry number: 1477511-61-7). Pale green solid. ^{1}H NMR (600 MHz, $CDCl_3$) δ 7.10 (AA'BB', 4H, aromatic), 7.14-7.17 (m, 8H, aromatic), 7.24-7.27 (m, 4H, aromatic), 7.37-7.39 (m, 4H, aromatic), 7.75 (AA'BB', 4H, aromatic). ^{13}C NMR (151 MHz, $CDCl_3$) δ 117.7, 125.2, 125.9, 127.3, 128.7, 129.2, 132.1, 134.7, 141.2, 148.9.

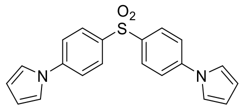

1,1'-(Sulfonylbis(4,1-phenylene))bis(1*H*-pyrrole) (3c). Pale brown solid. This product did not dissolve at temperatures below 300 °C, but turned black at 264 °C. ^{1}H NMR (600 MHz, $CDCl_3$) δ 6.38 (t, *J* = 2.2 Hz, 4H, aromatic), 7.11 (t, *J* = 2.2 Hz, 4H, aromatic), 7.50 (AA'BB', 4H, aromatic), 8.00 (AA'BB', 4H, aromatic). ^{13}C NMR (151 MHz, $CDCl_3$) δ 112.1, 119.0, 120.1, 129.5, 137.9, 144.2. IR (neat) 1595, 1509, 1475, 1423, 1331, 1308, 1288, 1152, 1126, 1105, 1084, 1064, 919, 840, 759, 737, 695, 681, 671, 621 cm^{-1}. HRMS calcd for $C_{20}H_{16}N_2O_2SNa$ (M + Na) 371.0825, found 371.0820.

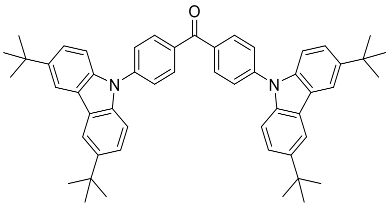

Bis(4-(3,6-di-*tert*-butyl-9*H*-carbazol-9-yl)phenyl)methanone (7a).[S1,S6-S7] Known chemical (CAS registry number: 1112364-38-1). Colorless solid. ^{1}H NMR (600 MHz, $CDCl_3$) δ 1.48 (s, 36H, *t*Bu), 7.50 (m, 8H, aromatic), 7.78 (AA'BB', 4H, aromatic), 8.13 (d, *J* = 1.6 Hz, 4H, aromatic), 8.14-8.16 (m, 4H, aromatic). ^{13}C NMR (151 MHz, $CDCl_3$) δ 32.0, 34.8, 109.3, 116.5, 123.91, 123.95, 125.9, 131.8, 135.4, 138.6, 142.4, 143.7, 194.6.

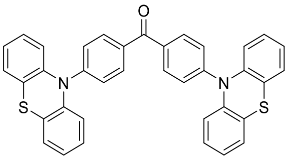

Bis(4-(10*H*-phenothiazin-10-yl)phenyl)methanone (7b).[S4,S8] Known chemical (CAS registry number: 1374412-39-1). Yellow solid. ^{1}H NMR (600 MHz, $CDCl_3$) δ 6.93 (dd, *J* = 1.1, 8.1 Hz, 4H, aromatic), 7.06 (dt, *J* = 1.3, 7.5 Hz, 4H, aromatic), 7.14 (ddd, *J* = 1.5, 6.4, 7.7 Hz, 4H, aromatic), 7.26 (AA'BB', 4H, aromatic), 7.29 (dd, *J* = 1.4, 7.7 Hz, 4H, aromatic), 7.85 (AA'BB', 4H, aromatic). ^{13}C NMR (151 MHz, $CDCl_3$) δ 121.4, 122.3, 124.8, 127.2, 128.1, 128.4, 132.2, 133.3, 142.3, 147.3, 194.2.

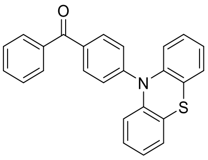

(4-(10*H*-Phenothiazin-10-yl)phenyl)(phenyl)methanone (9).[S4,S8] Known chemical (CAS registry number: 256340-35-9). ^{1}H NMR (600 MHz, $CDCl_3$) δ 7.00 (dbr, *J* = 8.0 Hz, 2H, aromatic), 7.08 (tbr, *J* = 7.0 Hz, 2H, aromatic), 7.17 (tbr, *J* = 7.4 Hz, 2H, aromatic), 7.26 (d, *J* = 8.7 Hz, 2H, aromatic), 7.31 (dd, *J* = 1.1, 7.7 Hz, 2H, aromatic), 7.48 (t, *J* = 7.7 Hz, 2H, aromatic), 7.57 (t, *J* = 7.4 Hz, 1H, aromatic), 7.82 (dd, *J* = 1.2, 7.2 Hz, 2H, aromatic), 7.84 (d, *J* = 8.6 Hz, 2H, aromatic). ^{13}C NMR (151 MHz, $CDCl_3$) δ 120.5, 122.9, 125.0, 127.2, 128.2, 128.3, 129.2, 129.8, 132.1, 132.4, 132.6, 138.0, 142.1, 147.8, 195.4.

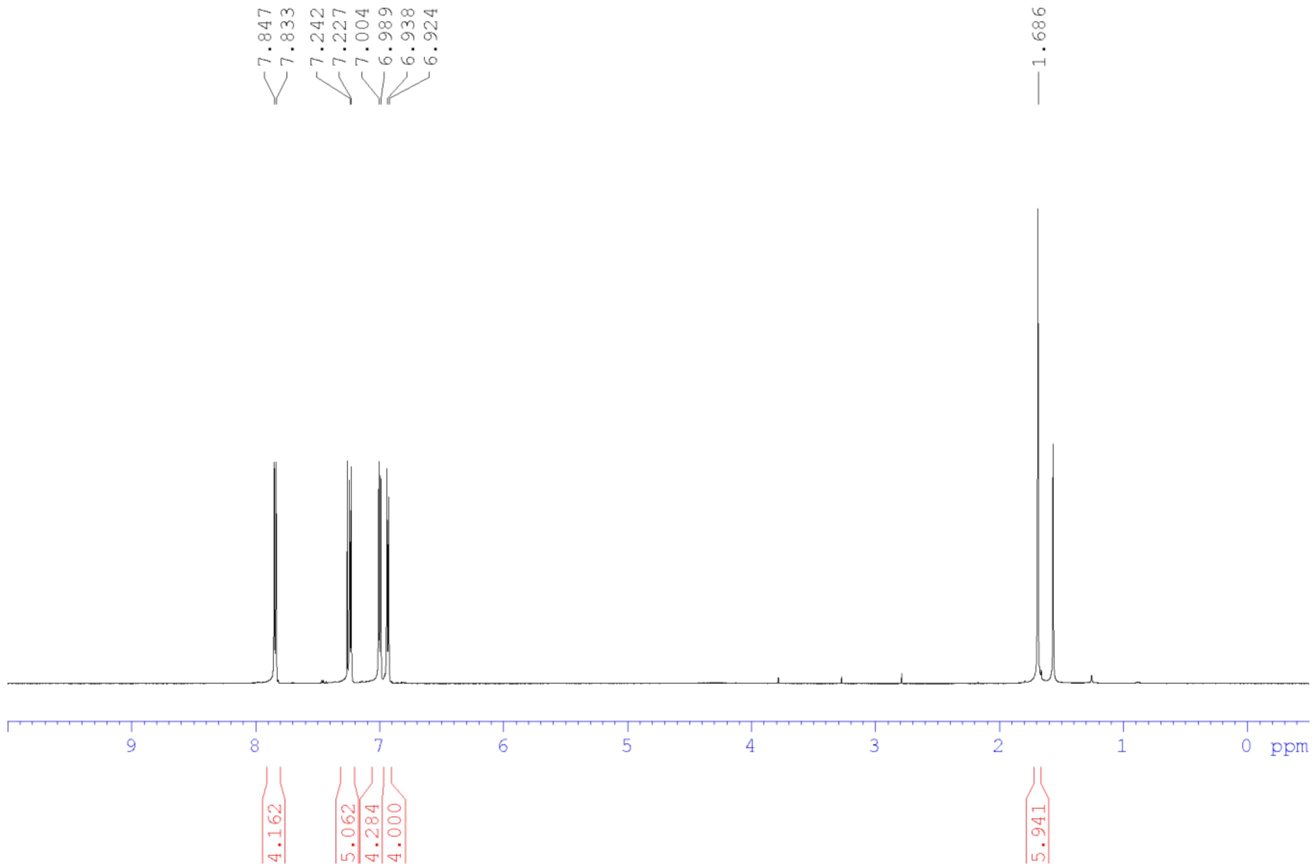

Fig. S1 | ^{1}H NMR spectroscopic analysis (600 MHz in $CDCl_3$) of PSU.

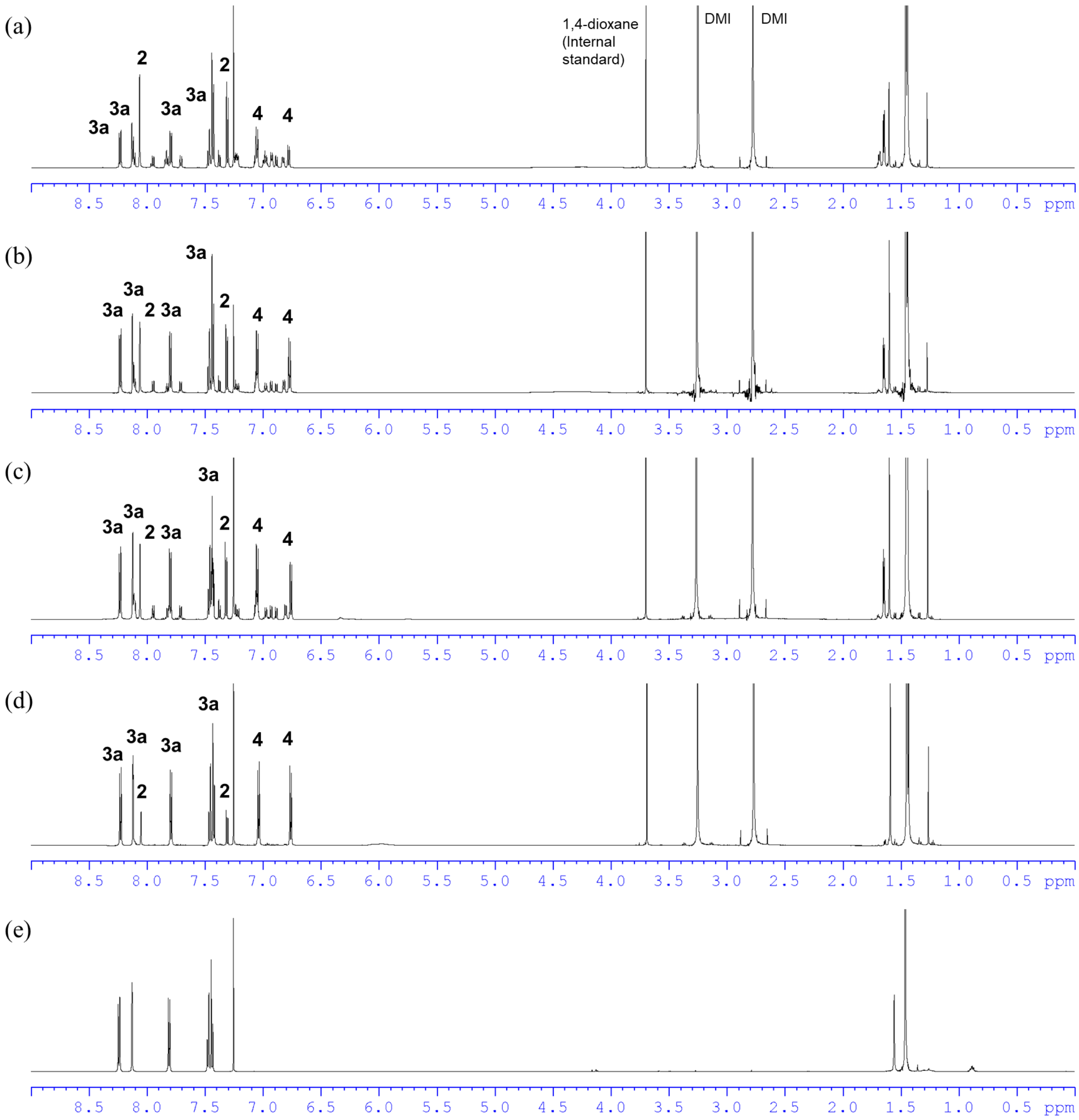

Fig. S2 | ^{1}H NMR spectroscopic analyses (600 MHz in $CDCl_3$) of the crude mixture of the reaction at (a) 0.5 h (Table 1, Entry 1), (b) 1 h (Table 1, Entry 2), (b) 2 h (Table 1, Entry 3), and (d) 5 h (Table 1, Entry 4) and (e) **3a**.

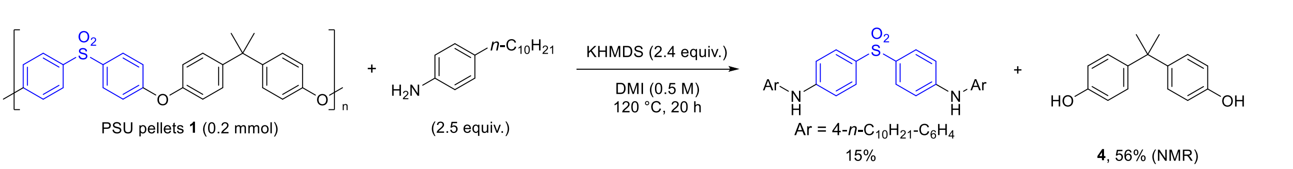

Fig. S3 | Reaction of PSU pellets **1** with 4-decylaniline to form 4,4'-sulfonylbis(*N*-(4-decylphenyl)-aniline)[S9] and bisphenol A.

4. Degradative surface functionalization of PEEK powder

4-1. Experimental procedure for the degradative surface functionalization of PEEK powder

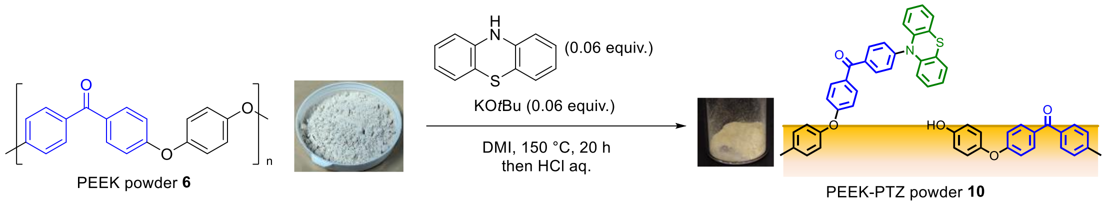

Degradative surface functionalization of PEEK powder 6 using 10*H*-phenothiazine (2b) and potassium *tert*-butoxide. To a mixture of PEEK powder **6** (287 mg, 1.0 mmol of PEEK relative to the molecular weight of the monomer), 10*H*-phenothiazine (**2b**) (10.9 mg, 0.055 mmol), and potassium *tert*-butoxide (6.3 mg, 0.056 mmol) was added 1,3-dimethyl-2-imidazolidinone (DMI, 0.8 mL) in a 3.0 mL vial under argon atmosphere. The resultant mixture was stirred at 150 °C for 20 h. After cooling to room temperature, HCl aq. (2 M, 1.0 mL) was added to quench the reaction. The resulting yellow solid was separated from the colorless solution via filtration and washed with water, methanol, and acetone. The obtained yellow solid was dried in vacuo to get yellow PEEK-PTZ powder **10** (266 mg, 93 wt.% based on used PEEK powder). The separated solution was analyzed by ^{1}H NMR spectroscopy, showing that phenothiazine was recovered (43% conversion).

Combustion analysis of this obtained powder showed average 0.14% nitrogen (two times average) and the existence of 0.010 mmol of the PTZ unit per 100 mg (0.027 mmol per 266 mg). This amount was comparable with the conversion analyzed by ^{1}H NMR spectroscopy.

The ^{1}H NMR spectroscopic analysis of a $CDCl_3$ suspension containing PEEK-PTZ **10** did not detect any observable PTZ-derived products such as **7b** (Fig. S4), showing that PEEK-PTZ did not have low-weight photo emissive compounds on the surface.

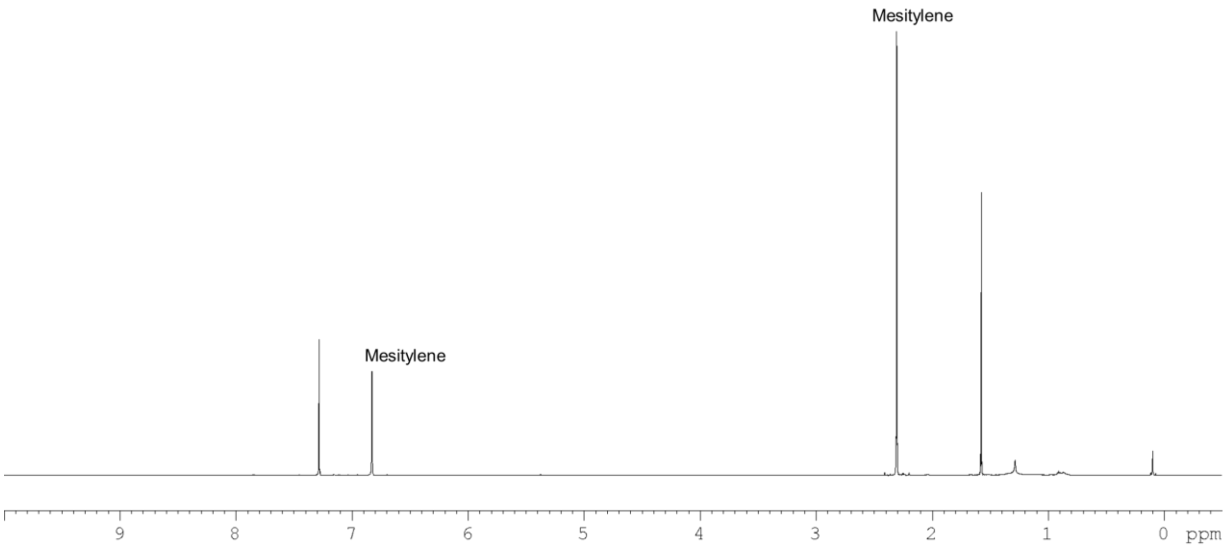

Fig. S4 | ^{1}H NMR spectroscopic analyses (600 MHz) of $CDCl_3$ containing PEEK-PTZ powder **10** with mesitylene as an internal standard.

Gram scale degradative surface functionalization of PEEK powder 6 using 10*H*-phenothiazine (2b) and potassium *tert*-butoxide. To a mixture of PEEK powder **6** (2.80 g, 9.69 mmol of PEEK relative to the molecular weight of the monomer), 10*H*-phenothiazine (**2b**) (106 mg, 0.53 mmol), and potassium *tert*-butoxide (54.5 mg, 0.49 mmol) was added 1,3-dimethyl-2-imidazolidinone (DMI, 8.0 mL) in a 20 mL vial under argon atmosphere. The resultant mixture was stirred at 150 °C for 20 h. After cooling to room temperature, HCl aq. (2 M, 2.0 mL) was added to quench the reaction. The resulting yellow solid was separated from the colorless solution via filtration and washed with water, methanol, and acetone. The obtained yellow solid was dried in vacuo to provide yellow PEEK-PTZ powder **10'** (2.75 g, 98 wt.% based on used PEEK powder). The separated solution was analyzed by ^{1}H NMR spectroscopy, showing that phenothiazine was recovered (46% conversion).

Combustion analysis of this obtained powder showed average 0.16% nitrogen and the existence of 0.011 mmol of the PTZ unit per 100 mg (0.31 mmol per 2.75 g). This introduction amount was comparable with the conversion analyzed by ^{1}H NMR spectroscopy.

The ^{1}H NMR spectroscopic analysis of a $CDCl_3$ suspension containing PEEK-PTZ **10'** did not detect any observable PTZ-derived products such as **7b** (Fig. S5), showing that PEEK-PTZ did not have low-weight photo emissive compounds on the surface.

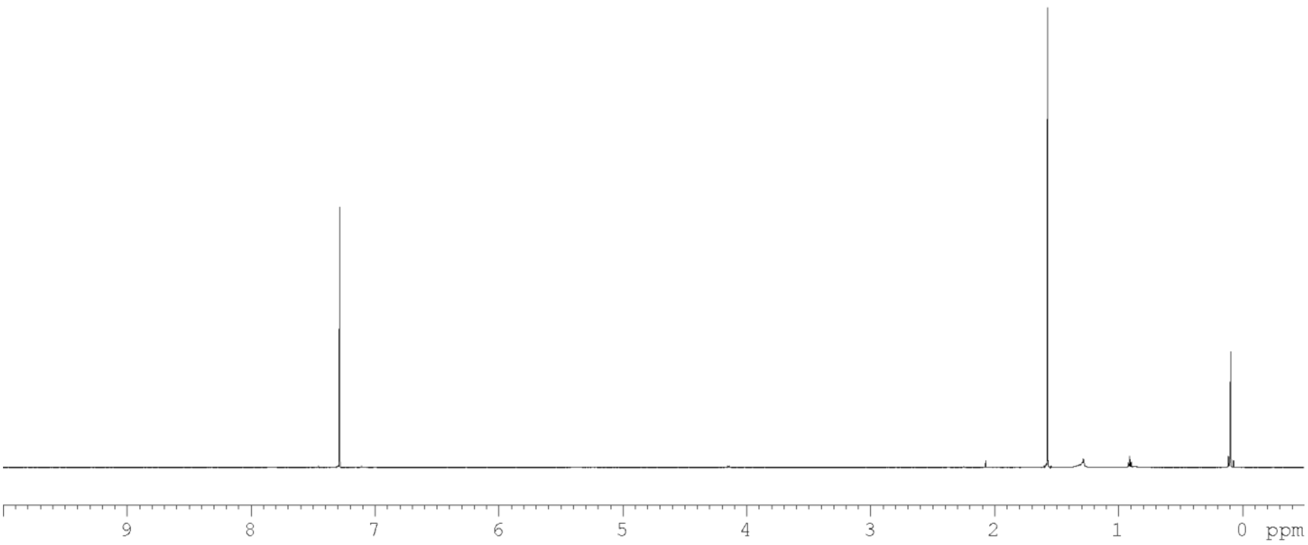

Fig. S5 | ^{1}H NMR spectroscopic analyses (600 MHz) of $CDCl_3$ containing PEEK-PTZ powder **10'**.

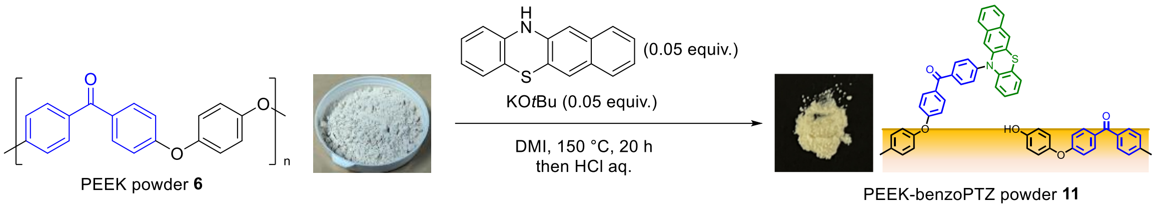

Degradative surface functionalization of PEEK powder by 12-phenyl-12*H*-benzo[b]phenothiazine (2d) and potassium *tert*-butoxide. To a mixture of PEEK powder **6** (2.88 g, 10.0 mmol of PEEK relative to the molecular weight of the monomer), 12*H*-benzo[*b*]phenothiazine (**2d**) (126 mg, 0.503 mmol), and potassium *tert*-butoxide (56.1 mg, 0.500 mmol) was added 1,3-dimethyl-2-imidazolidinone (DMI, 8.0 mL) in a 20 mL vial under argon atmosphere. The resultant mixture was stirred at 150 °C for 20 h. After cooling to room temperature, HCl aq. (2 M, 2.0 mL) was added to quench the reaction. The resulting yellow solid was separated from the colorless solution via filtration and washed with water, methanol, and acetone. The obtained yellow solid was dried in vacuo to provide yellow PEEK-benzoPTZ powder **11** (2.87 g, 99 wt.% based on used PEEK powder).

Combustion analysis of this obtained powder showed average 0.16% nitrogen and the existence of 0.011 mmol of the benzoPTZ unit per 100 mg (0.33 mmol per 2.87 g).

The ^{1}H NMR spectroscopic analysis of a DMSO-d_6 suspension containing PEEK-benzoPTZ **11** did not detect any observable benzoPTZ-derived products (Fig. S6), showing that PEEK-benzoPTZ did not have low-weight photo emissive compounds on the surface.

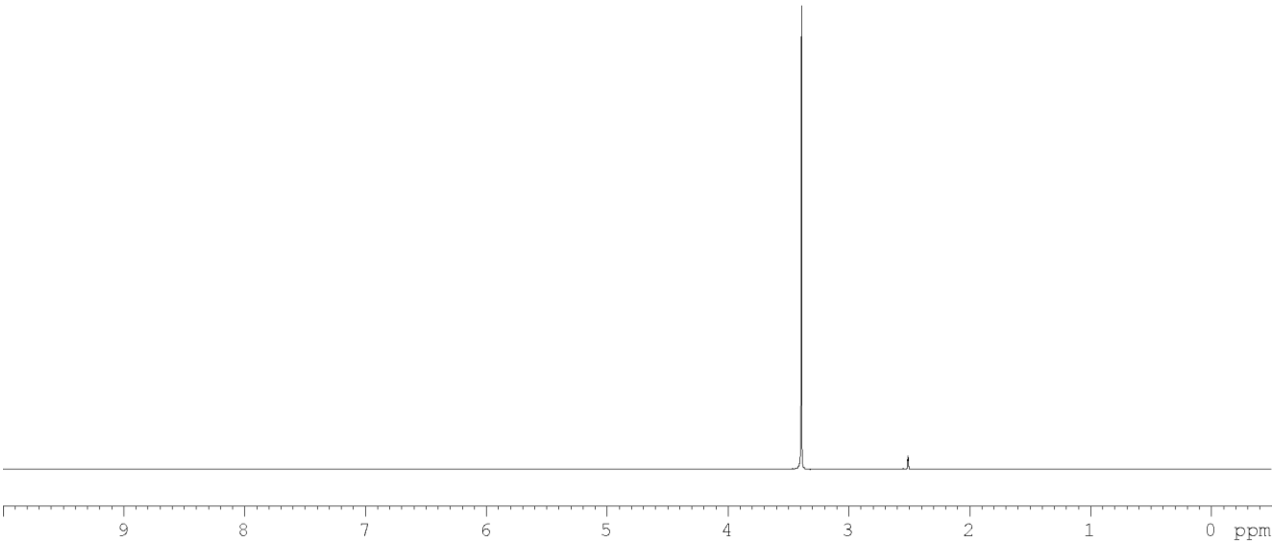

Fig. S6 | ^{1}H NMR spectroscopic analyses (600 MHz) of DMSO-d_6 containing PEEK-benzoPTZ powder **11**.

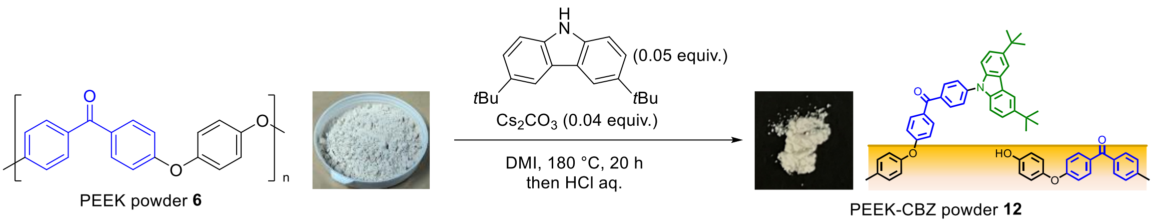

Degradative surface functionalization of PEEK powder by 3,6-di-*tert*-butylcarbazole (2a) and cesium carbonate. To a mixture of PEEK powder **6** (291 mg, 1.01 mmol of PEEK relative to the molecular weight of the monomer), 3,6-di-*tert*-butylcarbazole (**2a**) (13.1 mg, 0.047 mmol), and cesium carbonate (11.8 mg, 0.036 mmol) was added 1,3-dimethyl-2-imidazolidinone (DMI, 0.8 mL) in a 3.0 mL vial under argon atmosphere. The resultant mixture was stirred at 180 °C for 20 h. After cooling to room temperature, HCl aq. (2 M, 1.0 mL) was added to quench the reaction. The resulting yellow solid was separated from the colorless solution via filtration and washed with water, methanol, and acetone. The obtained yellow solid was dried in vacuo to provide yellow PEEK-CBZ powder **12** (279 mg, 96 wt.% based on used PEEK powder).

Combustion analysis of this obtained powder showed average 0.26% nitrogen and the existence of 0.019 mmol of the carbazole unit per 100 mg (0.052 mmol per 279 mg).

The ^{1}H NMR spectroscopic analysis of a $CDCl_3$ suspension containing PEEK-CBZ **12** detects negligible trace amount CBZ-derived products (Fig. S7). Especially, **7a** was not detected.

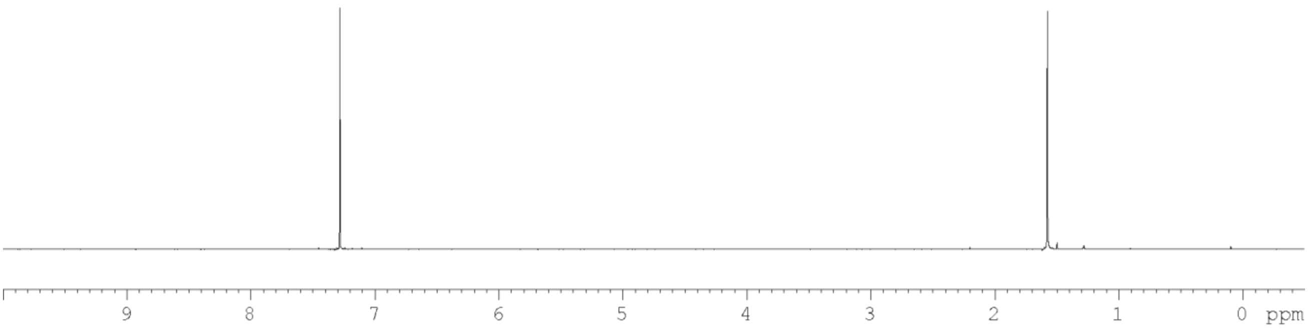

Fig. S7 | ^{1}H NMR spectroscopic analyses (600 MHz) of $CDCl_3$ containing PEEK-CBZ powder **12**.

Table S1 | Summary of the degradation of PEEK surface

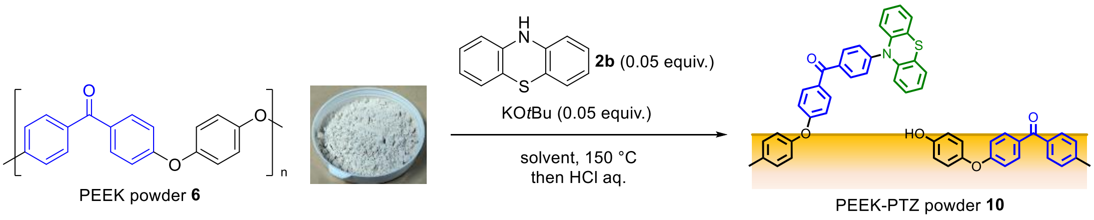

Entry	**6** (mmol)[b]	**2b** (mmol)	KO*t*Bu (mmol)	Solvent (mL)	Time (h)	PEEK-PTZ	**2b** conv. (%)	Degree of Crystallinity[c]	N /wt.%[d]
1-1	287 mg (0.997)	10.9 mg (0.0547)	6.3 mg (0.0561)	DMI (0.8)	20	**10**, 266 mg (93 wt.%)	43	1.86	0.14[d]
1-2	292 mg (1.01)	10.6 mg (0.0532)	6.4 mg (0.057)	DMI (0.8)	20	**10**^{rep}, 285 mg (98 wt.%)	57	1.89	0.18
2-1	287 mg (0.997)	10.0 mg (0.0502)	5.7 mg (0.0508)	DMAc (0.8)	20	**10**^{DMAc}, 281 mg (98 wt.%)	84	1.95	0.35
2-2	288 mg (0.999)	10.0 mg (0.0502)	6.2 mg (0.055)	DMAc (0.8)	20	**10**^{DMAcrep}, 282 mg (98 wt.%)	51	1.91	0.30
3	293 mg (1.02)	10.2 mg (0.0512)	5.9 mg (0.0523)	DMI (0.8)	1	**10**^{1h}, 286 mg (98 wt.%)	52	1.86	0.19
4	287 mg (0.997)	10.4 mg (0.0522)	5.6 mg (0.050)	DMI (0.8)	5	**10**^{5h}, 284 mg (99 wt.%)	54	1.95	0.18
5	2.80 g (9.69)	106 mg (0.53)	54.5 mg (0.49)	DMI (8.0)	20	**10'**, 2.75 g (98 wt.%)	46	1.85	0.16

[a] Unless otherwise noted, a mixture of PEEK powder **6**, **2b**, KO*t*Bu, and solvent was stirred at 150 °C. The resultant mixture was quenched by aq. HCl. Produced powder was obtained after washing with water, methanol, and acetone, and drying in vacuo. [b] Relative to the molecular weight of the monomer. [c] These values were calculated by the area ratios of intensities of the peaks between 1310 and 1280 cm^{-1} in the IR spectra. [d] Determined by combustion analysis.

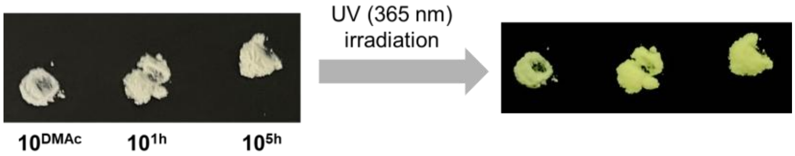

Fig. S8 | Observation of PEEK-PTZ **10**^{DMAc} (left), **10**^{1h} (middle), and **10**^{5h} (right) under 365 nm UV irradiation.

It is noteworthy that PEEK powder is insoluble in organic solvents and could not be analyzed at all by using high-temperature GPC with 1-chloronaphthalene at 220 °C. Also, PEEK-PTZ powder **10**, **10**^{DMAc}, and PEEK-CBZ **12** could not be analyzed by the high-temperature GPC.

4-2. Analytical data of functionalized PEEK powder

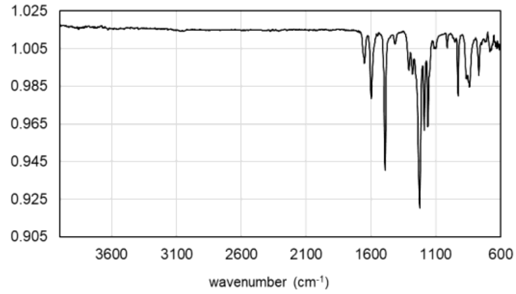

6

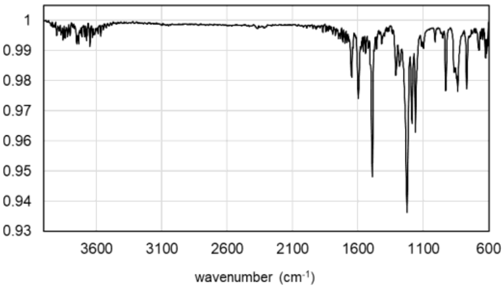

6-heat

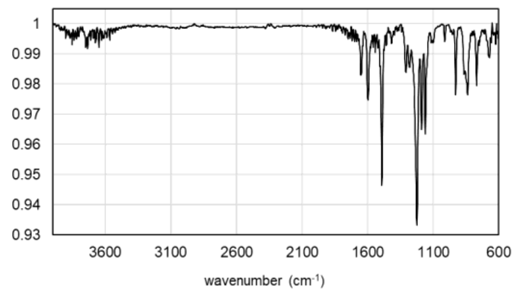

6-heat^{neat}

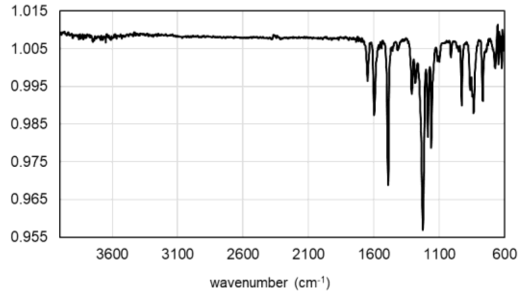

10

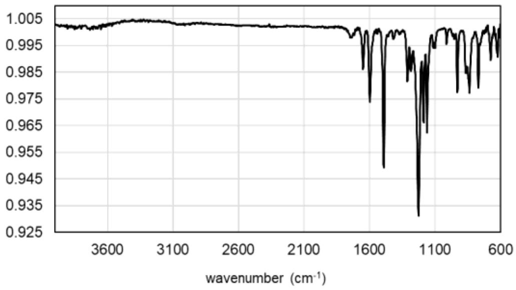

10’

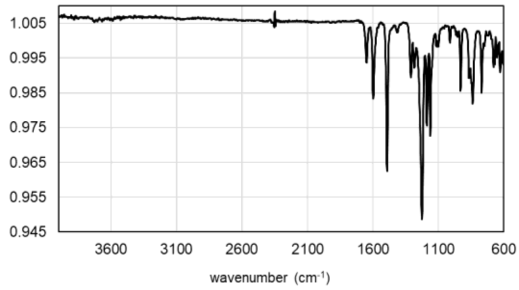

10^{DMAc}

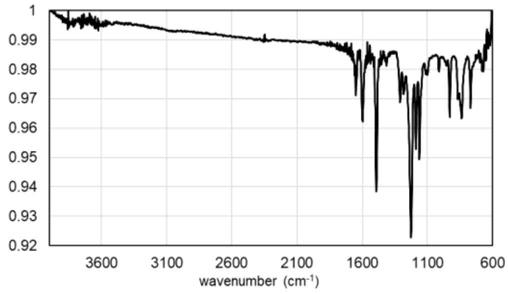

11

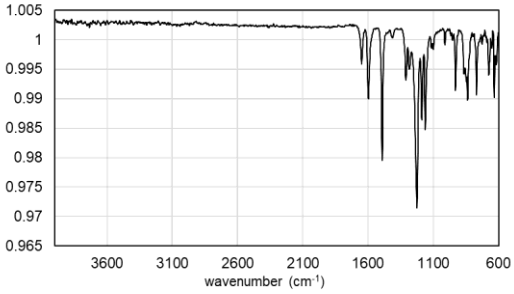

12

Fig. S9 | ATR-FTIR spectroscopic analyses of PEEK powder **6**, PEEK powder **6-heat** heated at 150 °C in DMI for 24 h, PEEK powder **6-heat^{neat}** heated at 150 °C for 24 h, PEEK-PTZ powder **10**, **10’**, **10^{DMAc}**, PEEK-benzoPTZ powder **11**, and PEEK-CBZ powder **12**.

IR (neat) of PEEK powder **6**: 1650, 1598, 1491, 1415, 1308, 1279, 1224, 1188, 1160, 1101, 1011, 951, 928, 839, 768, 682 cm^{-1}. Degree of crystallinity (area ratios of intensities of the peaks between 1310 cm^{-1} derived from crystalline moiety and 1280 cm^{-1} derived from crystalline and amorphous moieties)[S10], **6**: 0.75, **6-heat**: 1.86, **6-heat^{neat}**: 1.76, **10**: 1.86, **10’**: 1.85, **10^{DMAc}**: 1.95, **11**: 2.00, **12**: 2.00.

Above observed IR charts from **6**, **6-heat**, **6-heat^{neat}**, **10**, **10’**, **10^{DMAc}**, **11**, and **12** look same instead of the area ratios of intensities of the peaks between 1310 and 1280 cm^{-1}.

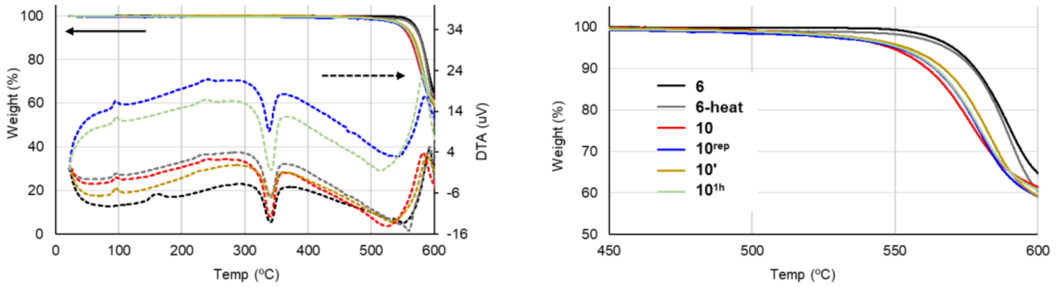

a)

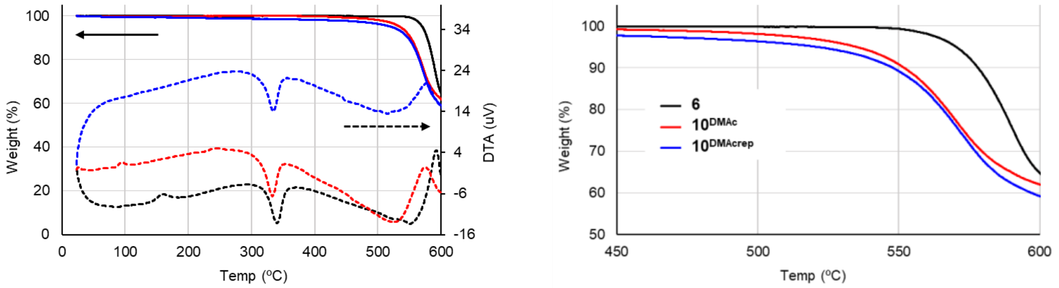

b)

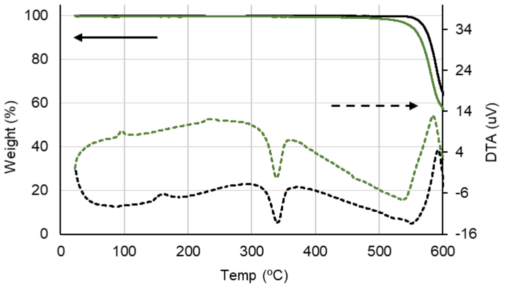

c)

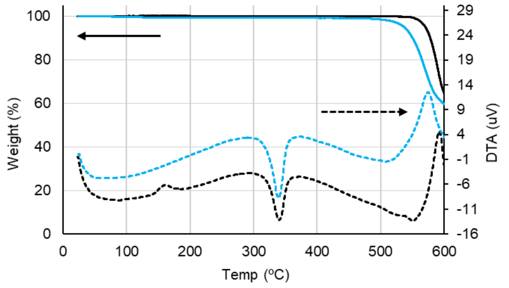

d)

Fig. S10 | Thermogravimetric analysis (TGA, bold line) and differential thermal analysis (DTA, dashed line) of products and PEEK powder **6** (black). **a** PEEK powder **6-heat** heated at 150 °C in DMI for 24 h (gray), PEEK-PTZ powder **10** (red), **10$^{\mathbf{rep}}$** (blue), **10'** (yellow), and **10$^{\mathbf{1h}}$** (pale green), **b** **10$^{\mathbf{DMAc}}$** (red) and **10$^{\mathbf{DMAcrep}}$** (blue), **c** **11** (green), and **d** **12** (pale blue).

TGA data of PEEK powder **6**: 0.00% 48.79 °C, -1.00% 554.25 °C, -5.00% 570.51 °C, -10.00% 578.15 °C, -20.00% 587.07 °C, -30.00% 594.32 °C. DTA data: T_p 161.35 °C, T_m 340.36 °C (onset).

TGA data of PEEK powder **6-heat** heated at 150 °C in DMI for 24 h: 0.00% 23.80 °C, -1.00% 499.95 °C, -5.00% 568.03 °C, -10.00% 577.19 °C, -20.00% 585.97 °C, -30.00% 592.07 °C, -40.00% 600.64 °C. DTA data: T_p 95.83, 250.78 °C, T_m 341.01 °C (onset).

TGA data of PEEK-PTZ powder **10**: 0.00% 53.10 °C, -1.00% 511.00 °C, -5.00% 549.51 °C, -10.00% 561.76 °C, -20.00% 573.72 °C, -30.00% 583.40 °C. DTA data: T_p 95.27, 235.76 °C, T_m 339.79 °C (onset).

TGA data of **10$^{\mathbf{rep}}$**: 0.00% 34.01 °C, -1.00% 478.60 °C, -5.00% 551.67 °C, -10.00% 564.37 °C, -20.00% 575.73 °C, -30.00% 583.71 °C, -40.00% 598.16 °C. DTA data: T_p 94.55, 241.76 °C, T_m 338.33 °C (onset).

TGA data of **10'**: 0.00% 107.54 °C, -1.00% 500.46 °C, -5.00% 554.11 °C, -10.00% 567.01 °C, -20.00% 578.32 °C, -30.00% 586.03 °C, -40.00% 597.93 °C. DTA data: T_p 94.78, 226.11 °C, T_m 340.42 °C (onset).

TGA data of $\mathbf{10^{DMAc}}$: 0.00% 34.84 °C, -1.00% 474.69 °C, -5.00% 535.85 °C, -10.00% 552.26 °C, -20.00% 573.72 °C, -30.00% 567.35 °C, -40.00% 579.72 °C. DTA data: T_p 94.55, 248.99 °C, T_m 333.14 °C (onset).

TGA data of $\mathbf{10^{DMAcrep}}$: 0.00% 30.32 °C, -1.00% 248.32 °C, -5.00% 523.14 °C, -10.00% 548.48 °C, -20.00% 565.87 °C, -30.00% 577.32 °C, -40.00% 597.79 °C. DTA data: T_m 334.37 °C (onset).

TGA data of $\mathbf{10^{1h}}$: 0.00% 66.77 °C, -1.00% 504.44 °C, -5.00% 552.68 °C, -10.00% 564.66 °C, -20.00% 576.10 °C, -30.00% 584.63 °C. DTA data: T_p 95.88, 238.46 °C, T_m 341.47 °C (onset).

TGA data of PEEK-benzoPTZ powder **11**: 0.00% 33.41 °C, -1.00% 496.34 °C, -5.00% 551.74 °C, -10.00% 564.34 °C, -20.00% 576.05 °C, -30.00% 584.00 °C, -40.00% 595.21 °C. DTA data: T_p 95.36, 239.44 °C, T_m 338.87 °C (onset).

TGA data of PEEK-CBZ powder **12**: 0.00% 40.64 °C, -1.00% 479.54 °C, -5.00% 537.48 °C, -10.00% 550.42 °C, -20.00% 565.24 °C, -30.00% 576.78 °C, -40.00% 599.44 °C. DTA data: T_m 339.29 °C (onset).

The analytic results between **10** and $\mathbf{10^{rep}}$, and $\mathbf{10^{DMAc}}$ and $\mathbf{10^{DMAcrep}}$, were almost the same, demonstrating the reproducibility of this surface functionalization. The comparison of TGA analyses of **10** with $\mathbf{10^{DMAc}}$ showed that the thermal stability was better maintained when synthesized in DMI solvent.

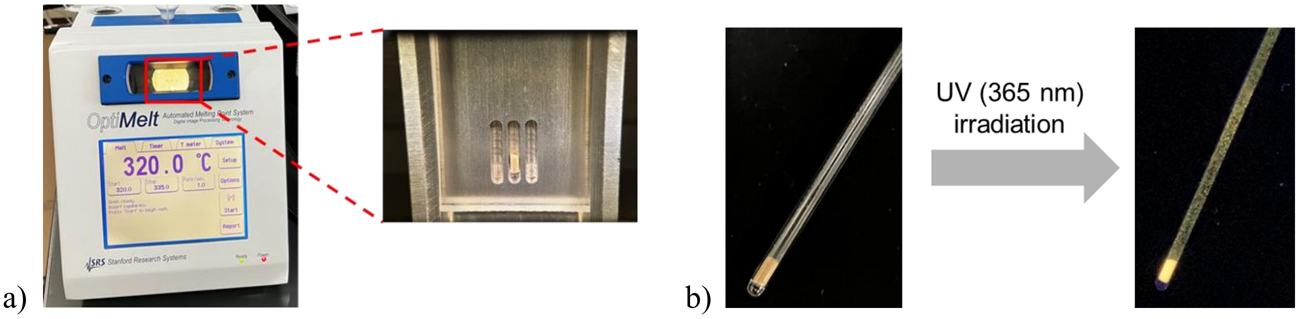

Fig. S11 | Heat test of PEEK-PTZ powder **10** at 320 °C for 3 h. **a** Photo image of this test using Optimelt Automated Melting Point System. **b** Photo image of heated **10**.

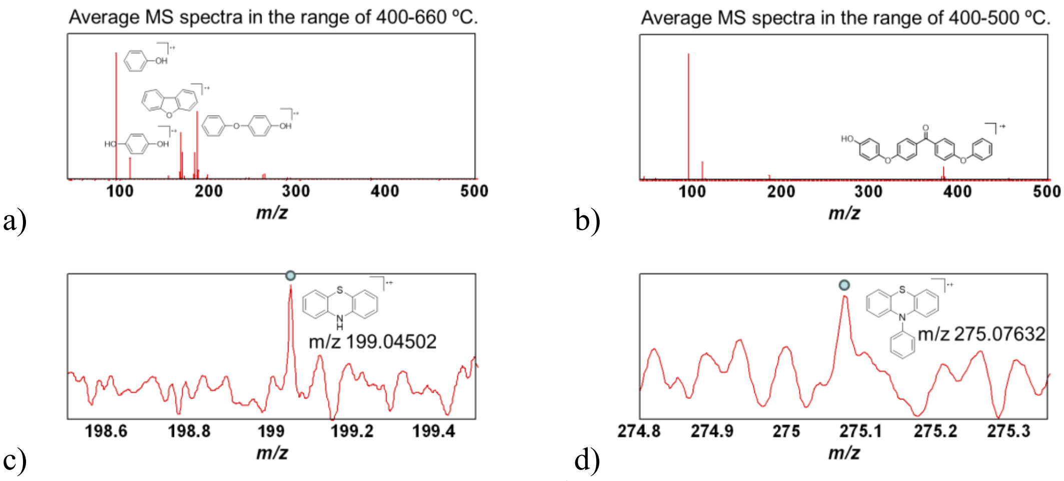

Fig. S12 | EGA-TOFMS analysis of 10^{DMAc}. a Average MS spectra in the range of 400-600 °C. **b** Average MS spectra in the range of 400-500 °C. **c** Enlarged view of the mass spectrum near m/z 199. **d** Enlarged view of the mass spectrum near m/z 275.

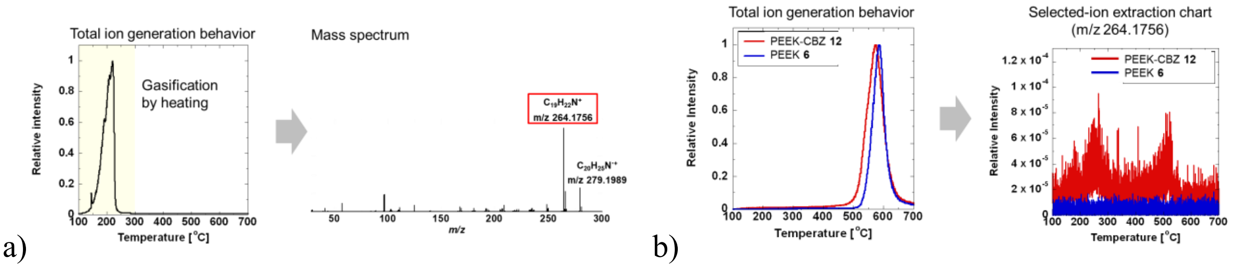

Fig. S13 | EGA-TOFMS analysis of 3,6-bis-*tert*-butylcarbazole (**2a**) and PEEK-CBZ powder **12**. **a** Total ion generation behavior and its mass spectrum of **7a**. **b** Total ion generation behavior and its mass spectra of **6** and **12**.

In EGA-TOFMS analysis of **10^{DMAc}**, average MS spectra in the range of 400-600 °C mainly detects pyrolysis products specific to the main chain of PEEK (Fig. S12a). Relatively large pyrolysis products such as m/z 382 were also detected near the starting temperature of pyrolysis (range of 400-500 °C) (Fig. S12b). Phenothiazine radical cation and *N*-phenyl-phenothiazine were slightly detected in the range of 400-600 °C (Fig. S12c and S12d). Notably, phenothiazine or benzophenothiazine unit was not detected by **10** or **11** maybe due to low introduced amounts.

In EGA-TOFMS analysis of **12**, a small amount of ions derived from 3,6-di-*tert*-butylcarbazole were detected (Fig. S13). Selected-ion extraction chart (m/z 264.1756) showed that ions were generated in two different temperature regions. The ions generated at higher temperatures (ca. 500 °C) may be components that are tightly bound to polymers.

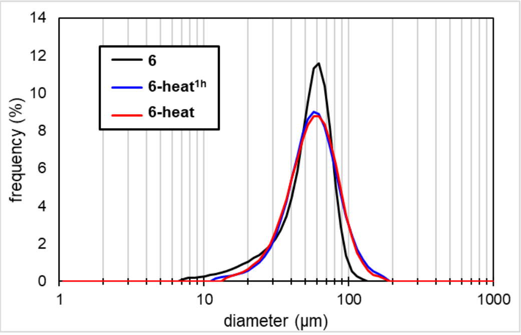

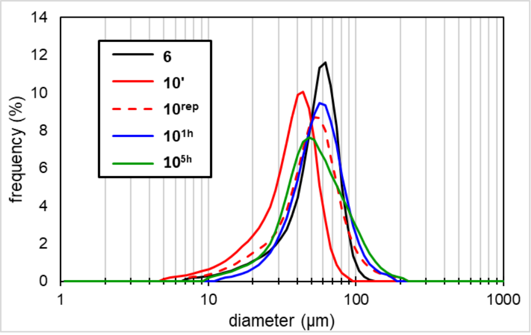

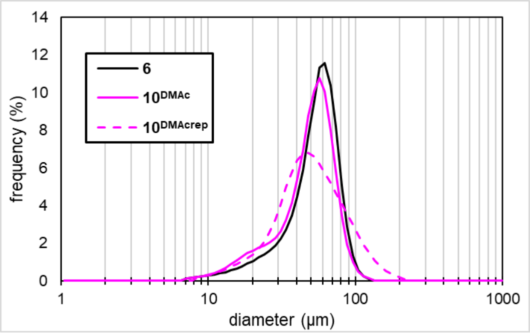

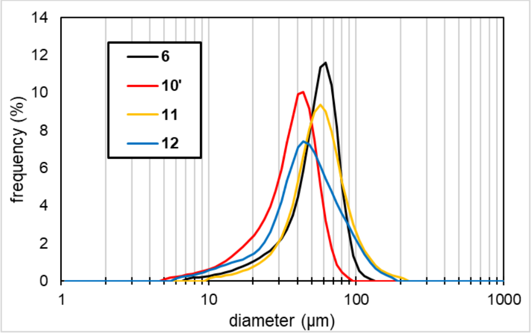

Resin	6	6-heat^{1h}	6-heat	10'	10^{rep}	10^{1h}	10^{5h}	10^{DMAc}	10^{DMAcrep}	11	12
MV (µm)	51.83	58.33	57.61	35.93	50.89	56.87	55.50	47.95	53.56	57.62	48.66
MN (µm)	17.56	29.95	31.99	12.15	16.70	29.83	23.13	17.17	19.27	25.09	13.36
MA (µm)	41.88	48.63	48.51	28.29	38.24	47.66	42.87	37.91	39.33	46.56	34.53
CS (m^2/mL)	0.14	0.12	0.12	0.21	0.16	0.13	0.14	0.16	0.15	0.13	0.17
SD (µm)	18.40	21.77	21.70	14.40	21.50	20.66	24.82	19.00	26.08	21.65	23.48
diameter (µm)	frequency (%)										
3.889	0	0	0	0	0	0	0	0	0	0	0
4.241	0	0	0	0	0	0	0	0	0	0	0
4.625	0	0	0	0	0	0	0	0	0	0	0
5.044	0	0	0	0.12	0	0	0	0	0	0	0
5.5	0	0	0	0.19	0	0	0	0	0	0	0
5.998	0	0	0	0.23	0	0	0	0	0	0	0.16
6.541	0	0	0	0.27	0	0	0	0	0	0	0.24
7.133	0.12	0	0	0.33	0.14	0	0	0.13	0	0	0.27
7.778	0.19	0	0	0.39	0.22	0	0	0.19	0	0	0.32
8.482	0.2	0	0	0.46	0.25	0	0	0.21	0.17	0	0.4
9.25	0.24	0	0	0.54	0.29	0	0	0.26	0.27	0	0.48
10.09	0.28	0	0	0.63	0.36	0	0.19	0.32	0.31	0.14	0.58
11	0.33	0	0	0.75	0.44	0	0.29	0.41	0.39	0.22	0.68
12	0.39	0.14	0	0.9	0.54	0.13	0.34	0.52	0.5	0.24	0.78
13.08	0.46	0.22	0	1.08	0.66	0.2	0.42	0.68	0.62	0.29	0.88
14.27	0.55	0.25	0.19	1.3	0.81	0.24	0.53	0.87	0.75	0.36	0.97
15.56	0.66	0.3	0.31	1.54	0.97	0.3	0.63	1.07	0.89	0.44	1.06
16.96	0.78	0.37	0.39	1.82	1.15	0.39	0.75	1.29	1.04	0.53	1.15
18.5	0.91	0.47	0.5	2.11	1.33	0.51	0.88	1.47	1.21	0.64	1.28
20.17	1.06	0.59	0.66	2.43	1.51	0.66	1.04	1.62	1.41	0.78	1.44
22	1.23	0.76	0.87	2.82	1.71	0.87	1.26	1.75	1.69	0.94	1.69
23.99	1.41	0.99	1.14	3.29	1.92	1.14	1.55	1.86	2.06	1.15	2.05
26.16	1.62	1.32	1.51	3.95	2.2	1.5	2	2.02	2.58	1.43	2.59
28.53	1.89	1.77	1.99	4.79	2.55	1.97	2.62	2.26	3.26	1.8	3.31
31.11	2.25	2.4	2.63	5.93	3.07	2.58	3.46	2.65	4.06	2.34	4.22
33.93	2.73	3.22	3.43	7.34	3.75	3.34	4.55	3.22	4.97	3.06	5.32
37	3.47	4.24	4.38	8.67	4.67	4.28	5.63	4.13	5.76	4.05	6.28

40.35	4.46	5.45	5.46	9.91	5.83	5.41	6.71	5.37	6.42	5.3	7.08
44	5.88	6.65	6.52	10.05	6.99	6.62	7.38	6.93	6.78	6.64	7.4
47.98	7.73	7.83	7.54	9.16	8.16	7.89	7.64	8.81	6.8	8.06	7.25
52.33	9.53	8.63	8.3	7.49	8.71	8.85	7.5	10.09	6.62	8.96	6.82
57.06	11.33	9.03	8.77	5.07	8.67	9.47	6.97	10.78	6.22	9.36	6.11
62.23	11.58	8.89	8.77	3.2	7.97	9.33	6.38	10.06	5.74	9.01	5.41
67.86	10.38	8.19	8.35	1.73	6.6	8.49	5.72	7.99	5.2	7.93	4.74
74	8.19	7.16	7.48	0.87	5.22	7.23	5.07	5.81	4.65	6.67	4.12
80.7	5.05	5.85	6.22	0.45	3.82	5.6	4.47	3.47	4.1	5.24	3.56
88	2.87	4.59	4.89	0.19	2.74	4.17	3.85	1.93	3.54	4.01	2.99
95.96	1.34	3.41	3.5	0	1.93	2.92	3.22	0.99	2.99	2.99	2.44
104.7	0.55	2.46	2.39	0	1.36	2	2.6	0.47	2.45	2.19	1.91
114.1	0.25	1.71	1.53	0	0.98	1.36	1.99	0.25	1.93	1.6	1.4
124.5	0.09	1.17	0.95	0	0.71	0.92	1.47	0.12	1.48	1.16	1
135.7	0	0.79	0.58	0	0.53	0.64	1.03	0	1.08	0.83	0.67
148	0	0.54	0.36	0	0.4	0.45	0.7	0	0.76	0.59	0.45
161.4	0	0.39	0.25	0	0.3	0.34	0.47	0	0.52	0.41	0.32
176	0	0.22	0.14	0	0.23	0.2	0.32	0	0.36	0.29	0.18
191.9	0	0	0	0	0.19	0	0.23	0	0.27	0.22	0
209.3	0	0	0	0	0.12	0	0.14	0	0.15	0.13	0
228.2	0	0	0	0	0	0	0	0	0	0	0
248.9	0	0	0	0	0	0	0	0	0	0	0
271.4	0	0	0	0	0	0	0	0	0	0	0
296	0	0	0	0	0	0	0	0	0	0	0

Fig. S14 | Results of particle size distribution of PEEK powder **6**, PEEK powder **6-heat^{1h}** and **6-heat** heated at 150 °C for 1 h and 24 h in DMI, respectively, PEEK-PTZ powder **10'**, **10^{rep}**, **10^{1h}**, **10^{5h}**, **10^{DMAc}**, **10^{DMAcrep}**, PEEK-benzoPTZ powder **11**, and PEEK-CBZ powder **12**. MV: mean volume diameter, MN: mean number diameter, MA: mean area diameter, CS: specific surface area, SD: standard deviation.

a)

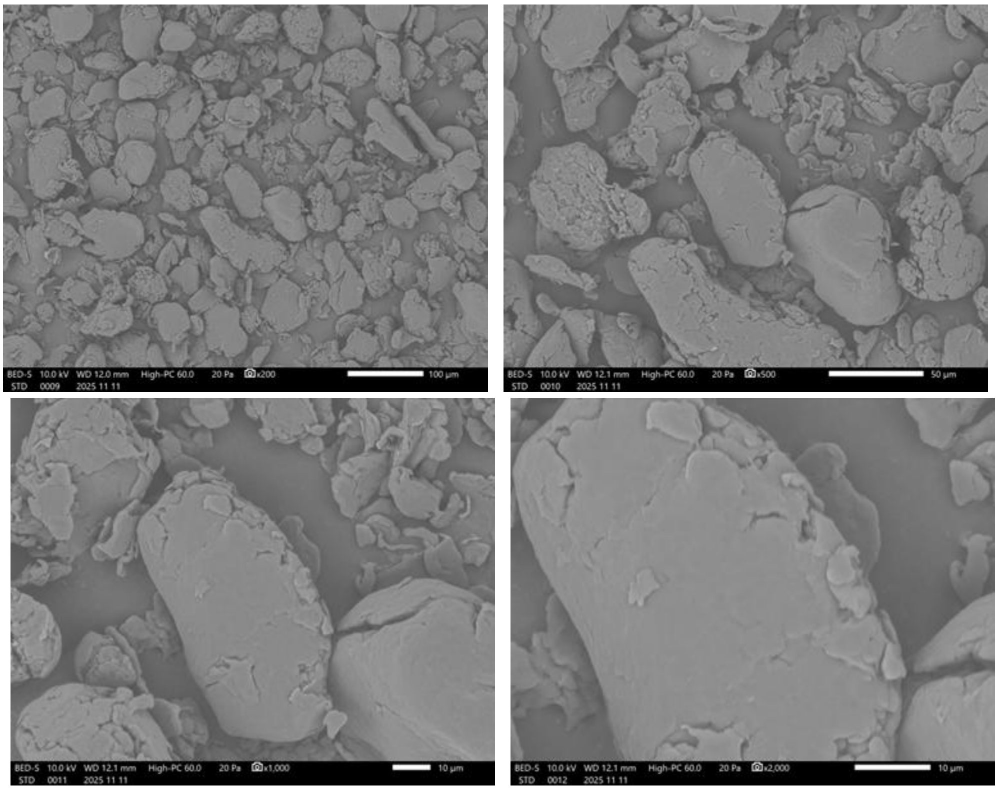

b)

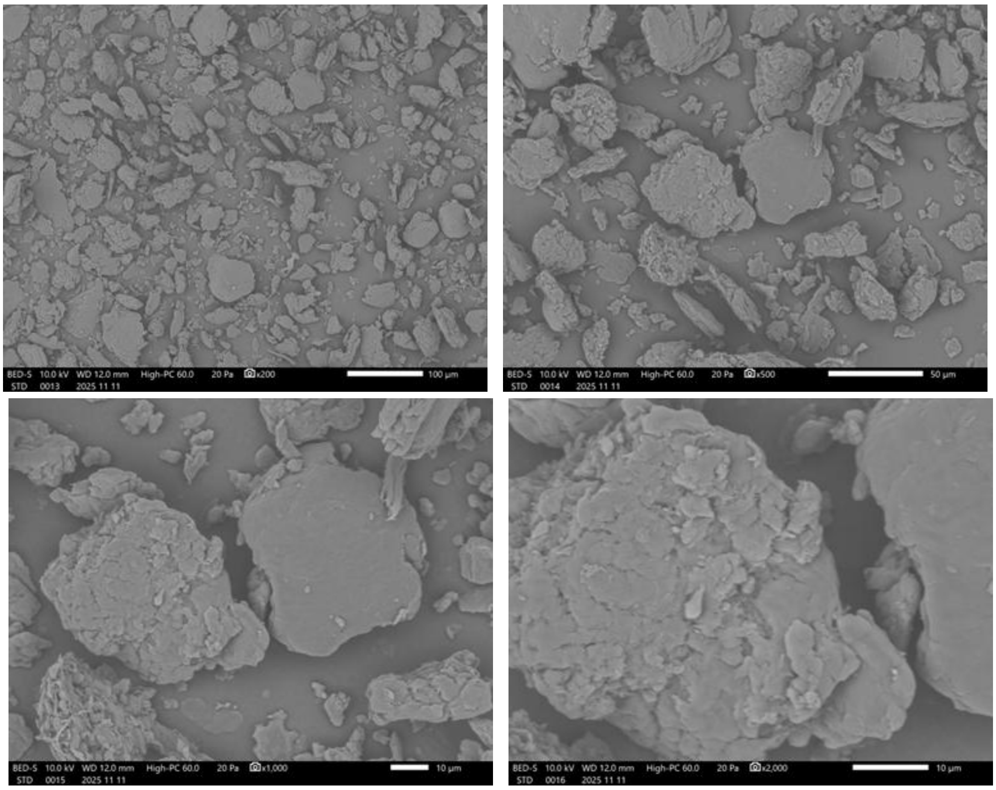

Fig. S15 | a SEM images of PEEK powder **6** (top reft: ×200, top right: ×500, bottom left: ×1,000, bottom right: ×2,000). **b** SEM images of PEEK-PTZ powder **10’** (top reft: ×200, top right: ×500, bottom left: ×1,000, bottom right: ×2,000).

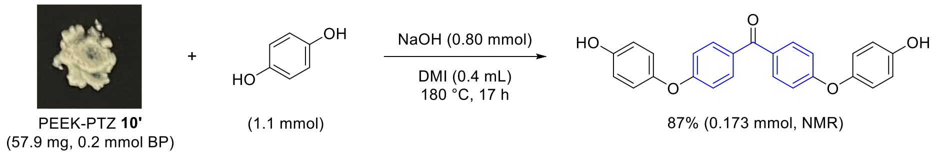

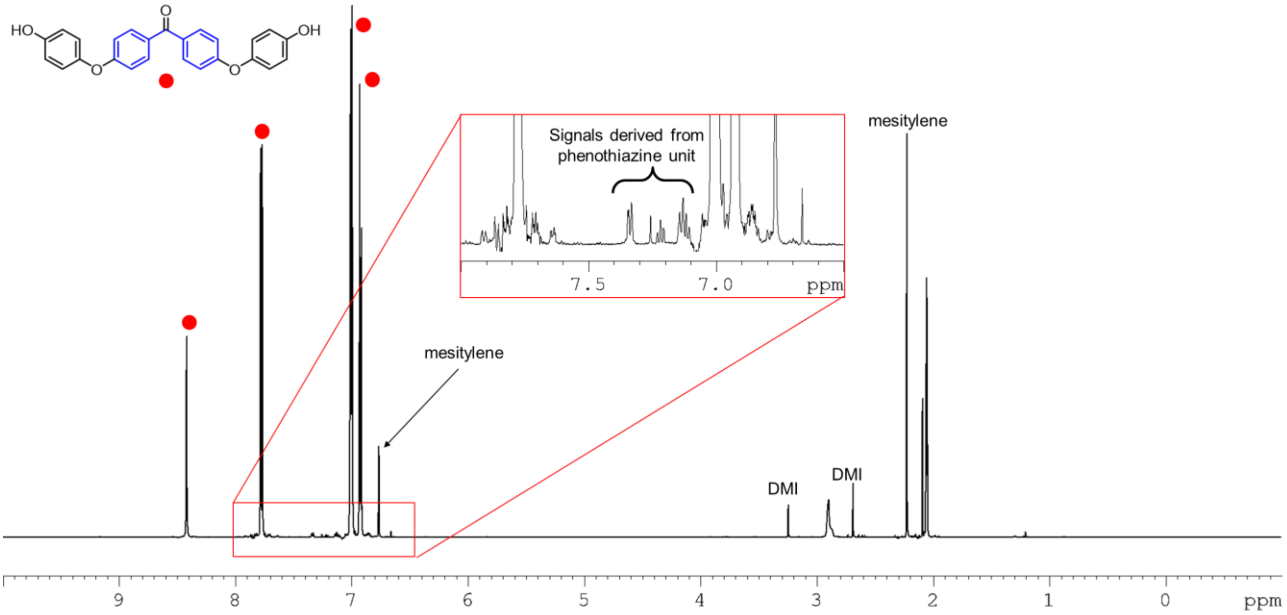

Fig. S16 | Depolymerization of 10' with hydroquinone.[S11] To a mixture of **10'**, NaOH, and hydroquinone was added DMI. After the resultant mixture was stirred at 180 °C for 17 h, the degradation mixture was quenched using hydrogen chloride (4 M) in 1,4-dioxane. The obtained solution was analyzed by ^{1}H NMR spectroscopy using acetone-d_6 solvent and mesitylene as an internal standard to determine the yields of the products. In this case, some signals likely derived from phenothiazine unit were detected.

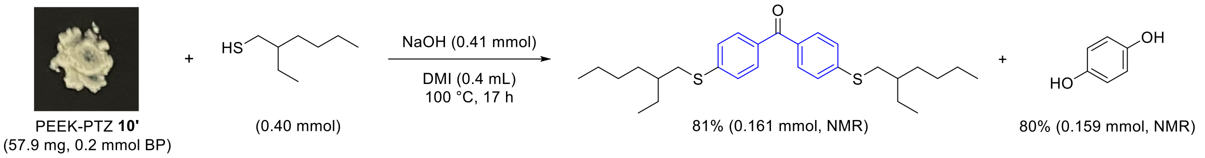

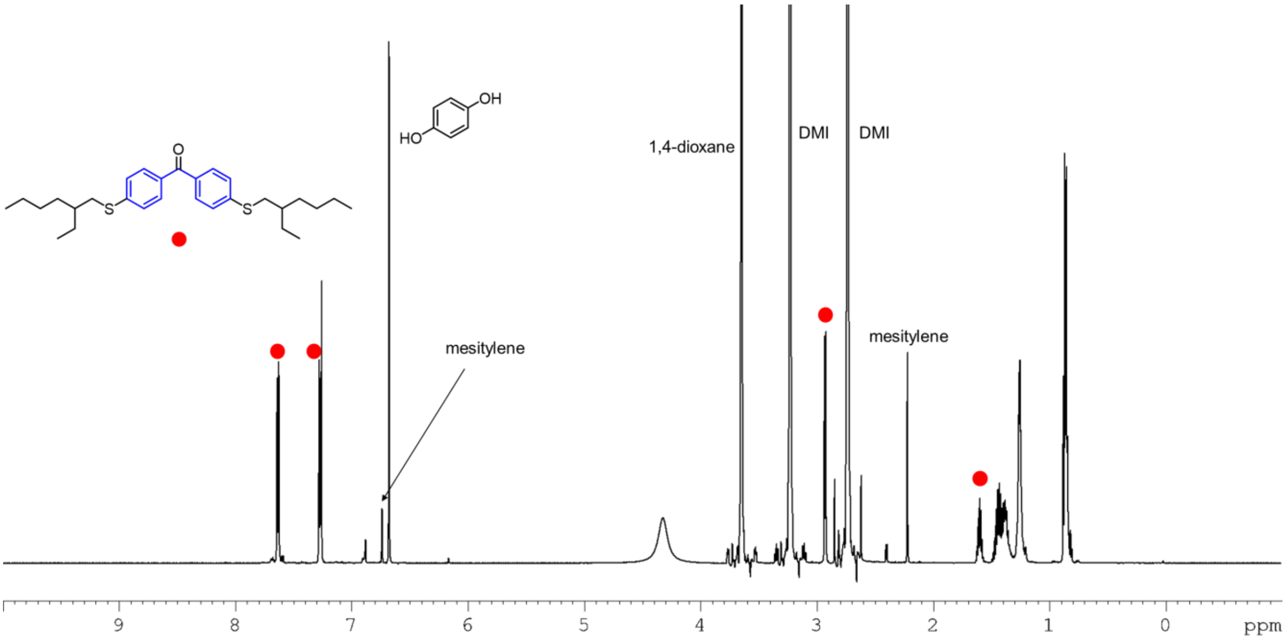

Fig. S17 | Degradation of 10’ with 2-ethylhexanethiol.[S9,S12] To a mixture of **10’** and NaOH were added DMI and thiol. After the resultant mixture was stirred at 100 °C for 17 h, the degradation mixture was quenched using hydrogen chloride (4 M) in 1,4-dioxane. The obtained solution was analyzed by ^{1}H NMR spectroscopy using $CDCl_3$ solvent and mesitylene as an internal standard to determine the yields of the products.

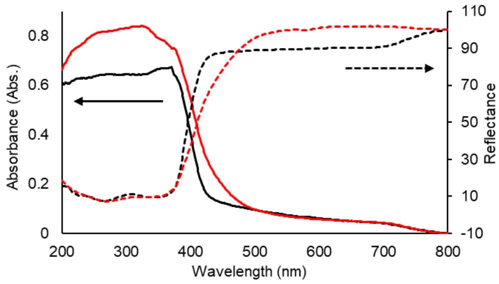

10

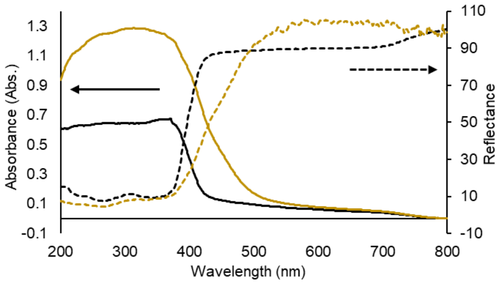

11

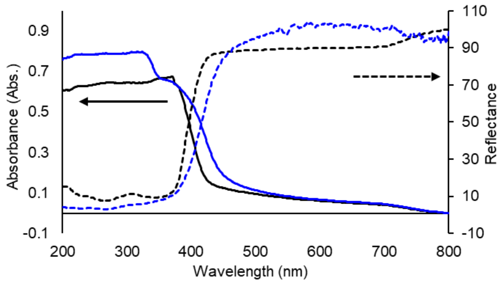

12

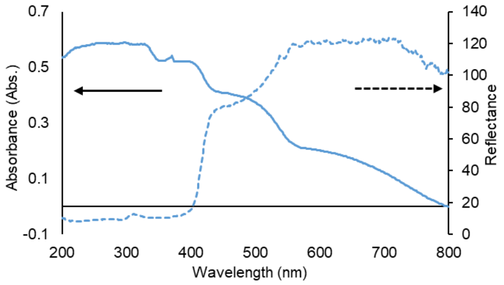

7a

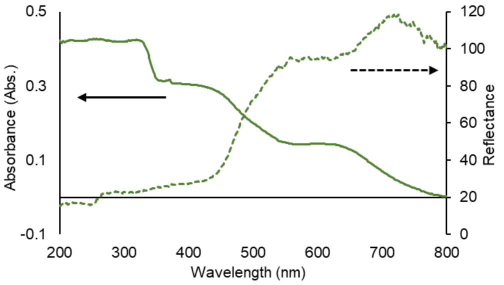

7b

Fig. S18 | Absorption (bold) and reflectance (dash) spectra of PEEK-PTZ powder **10** (red), PEEK-benzoPTZ powder **11** (yellow), and PEEK-CBZ powder **12** (blue) with PEEK powder **6** (black), and **7a** (pale blue) and **7b** (green).

These absorption and reflection analyses show that the absorption property of PEEK-PTZ **10** is slightly affected by the benzophenone-phenothiazine unit on the surface. PEEK-PTZ can absorb light at a wavelength of 405 nm to be excited rather than normal PEEK. In this relation, PEEK-benzoPTZ **11** and PEEK-CBZ **12** also showed the same trend as **10**.

PEEK powder **6**

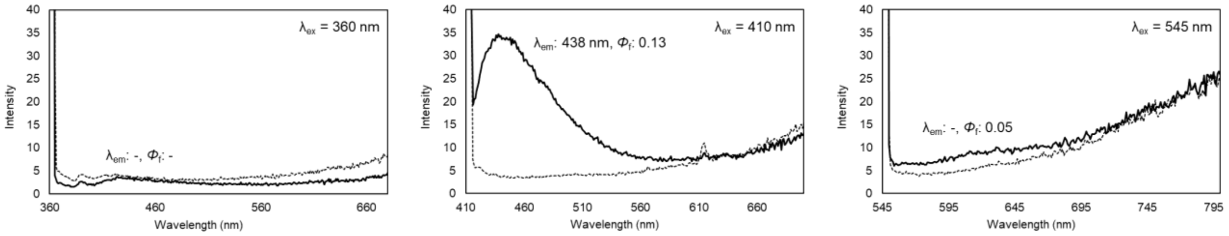

7b

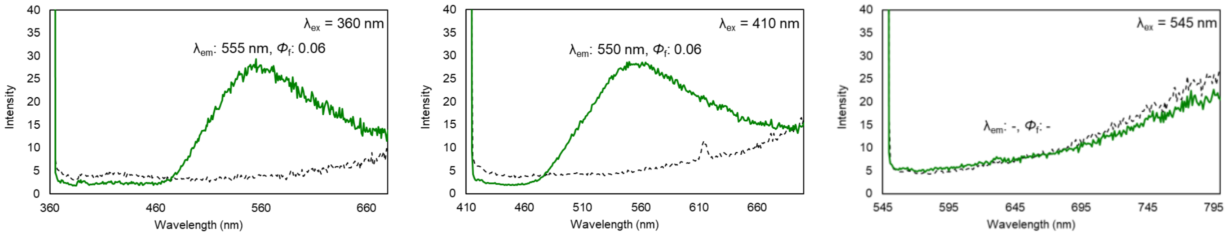

PEEK-PTZ (**10**)

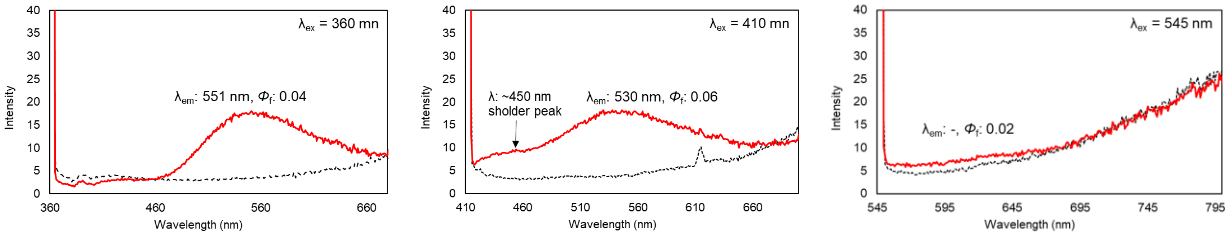

10^{DMAc}

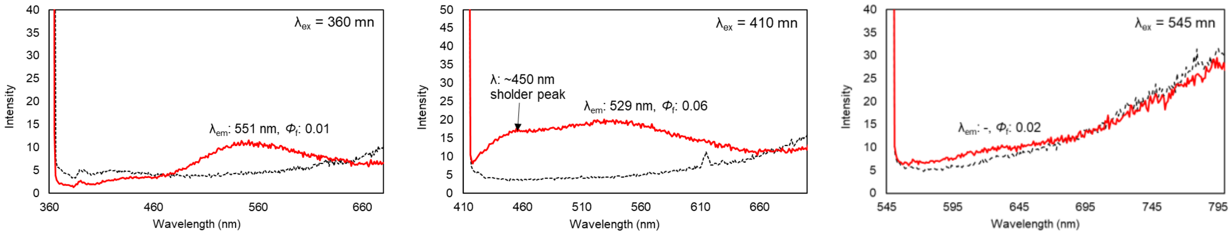

10'

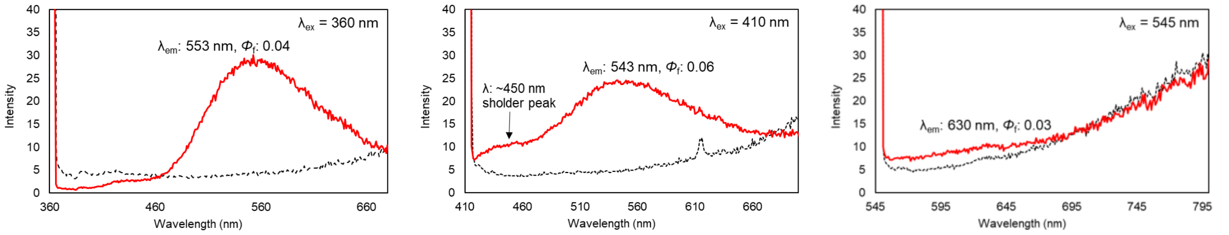

10^{1h}

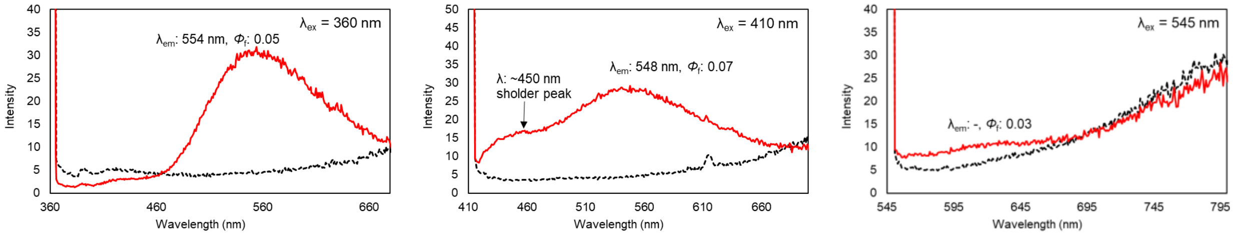

10^{5h}

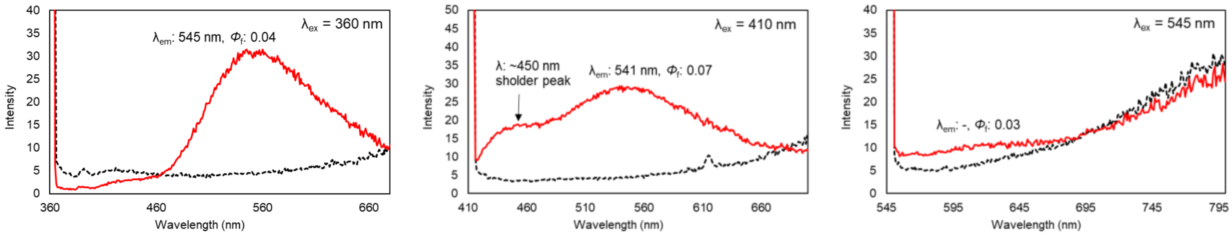

Fig. S19 | Photoluminescence spectra at a powder state with emission maxima (λ_{em}) and absolute quantum efficiencies (Φ_f). PEEK powder **6** (black/solid), **7b** (green/solid), PEEK-PTZ powder **10** (red/solid), $\mathbf{10^{DMAc}}$ (red/solid), **10'** (red/solid), $\mathbf{10^{1h}}$ (red/solid), and $\mathbf{10^{5h}}$ (red/solid) with blank chart (black/dash) obtained by the irradiation of 360, 410, and 545 nm light.

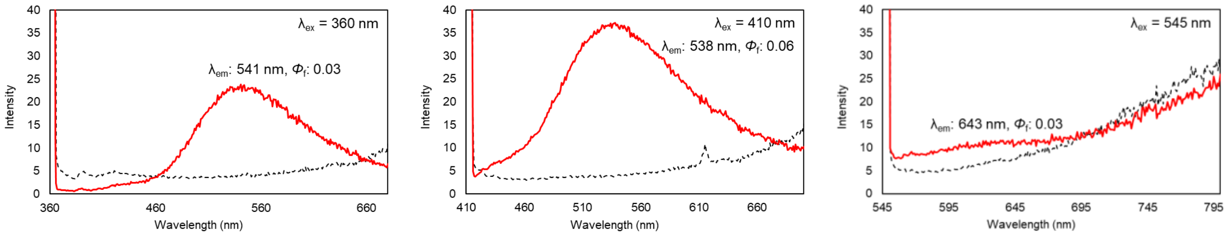

Fig. S20 | Fluorescence spectra of PEEK-benzoPTZ (**11**) at a powder state with emission maxima (λ_{em}) and absolute quantum efficiencies (Φ_f). **11** (red/solid) with blank chart (black/dash) obtained by the irradiation of 360, 410, and 545 nm light.

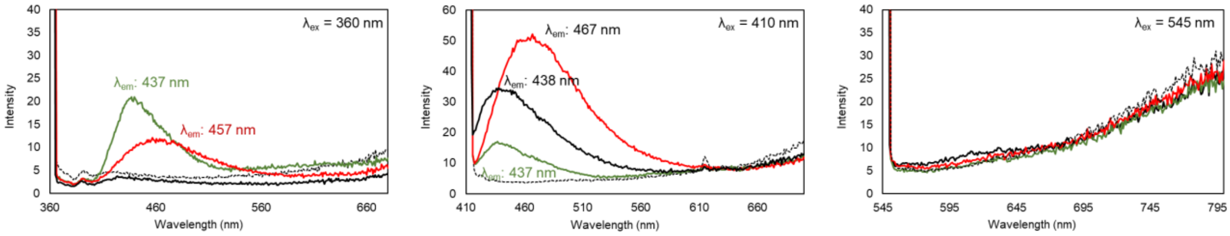

Fig. S21 | Fluorescence spectra of **7a** and PEEK-carbazole powder **12** at a powder state with emission maxima (λ_{em}). **7a** (green/solid) and PEEK-CBZ powder **12** (red/solid) with PEEK powder **6** (black), and blank chart (black/dash) obtained by the irradiation of 360, 410, and 545 nm light.

5. Catalytic reduction of 4-halobenzonitrile to benzonitrile under photo irradiation.[S13]

5-1. Procedure.

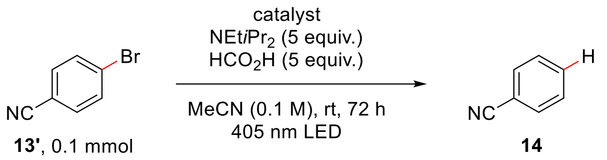

To a mixture of 4-bromobenzonitrile **13'** (18.5 mg, 0.102 mmol), PEEK-PTZ **10'** (5.1 mg, 0.5 mol%), and acetonitrile (1.0 mL) was added diisopropylethylamine (90 µL, 0.53 mmol) and formic acid (20 µL, 0.53 mmol) in a 3.0 mL vial under argon atmosphere. The resultant mixture was stirred at 30 °C (measured by thermometer) under 400-410 nm photoirradiation using LED light purchased from Aldrich (catalog No. ALDRP2, 400-410 nm) at 1.5 to 2 cm distance for 72 h (Fig. S18). The reaction mixture was cooled to room temperature. After mesitylene (5.0 µL, internal standard) was added to the reaction solution, the mixture was analyzed by GC to determine the yield of benzonitrile **14** in 82% (three times average (80%, 82%, 82%)) and full consumption of **13'**. The mixture was also analyzed by ^{1}H NMR spectroscopy, showing that **14** was obtained in 79% yield (three times average (76%, 81%, 81%)).

Based on this experimental protocol, the reaction using 4-chlorobenzonitrile (**13**) was examined.

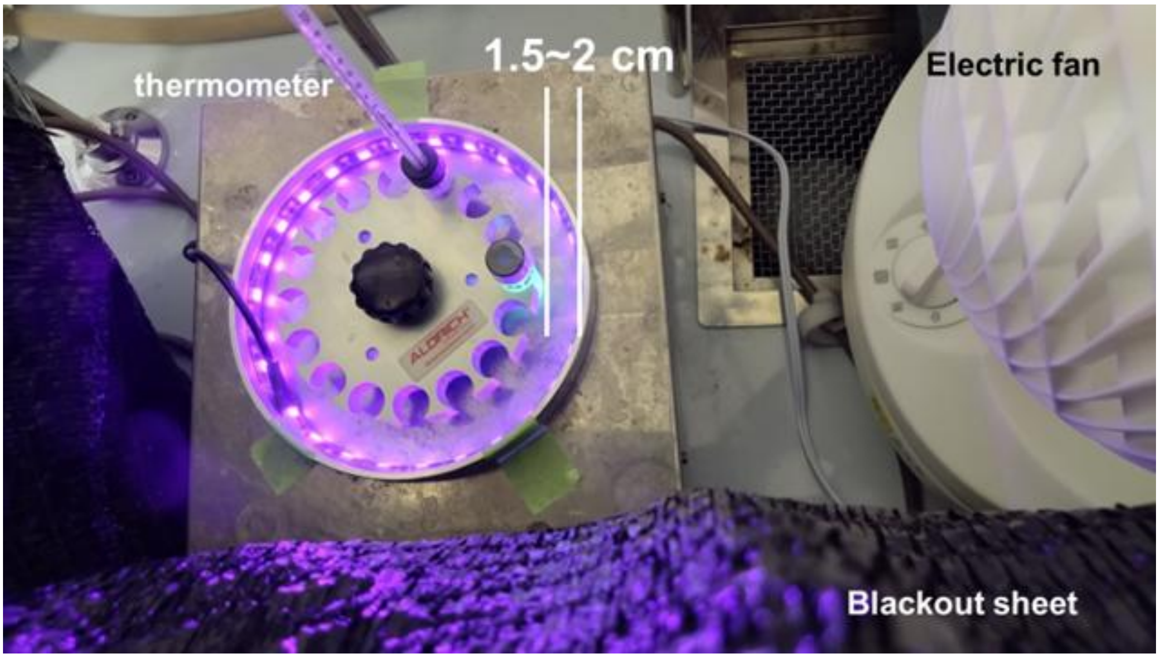

Fig. S22 | Reaction using photoredox catalysts under photo irradiation by purple LED light ring for Aldrich® micro photochemical reactor (spectral range 400-410 nm) at a distance of 1.5-2.0 cm.

Caution: It is important to keep the distance between the solution in the reaction vials and this light source purchased from Aldrich at 1.5-2.0 cm in this experiment using **13'**. When the distance

between the solution and the light source is reduced to 0.1 cm, the reaction proceeds without a catalyst to form **14** in 51% yield.

Table S2 | Catalytic dehalogenation of 4-bromobenzonitrile (13').[a]

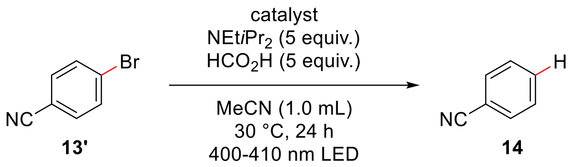

Run	13' (mmol)	Catalyst	Time	**14** (%)
1-1	0.1	-	24 h	0
1-2	0.1	-	24 h	3
2	0.1	*N*-Ph-PTZ (5 mol%)	24 h	69
3-1	0.1	PEEK powder **6** (4.9 mg, 17 mol% PEEK)	24 h	5
3-2	0.1	PEEK powder **6** (5.2 mg, 18 mol% PEEK)	24 h	5
4-1	0.1	PEEK-PTZ **10** (4.7 mg, 0.5 mol% BP-PTZ)	24 h	31
4-2	0.1	PEEK-PTZ **10** (5.1 mg, 0.5 mol% BP-PTZ)	24 h	36
4-3	0.1	PEEK-PTZ **10** (4.9 mg, 0.5 mol% BP-PTZ)	24 h	34
5	0.1	PEEK-PTZ $\mathbf{10^{rep}}$ (5.0 mg, 0.5 mol% BP-PTZ)	24 h	24
6	0.1	PEEK-PTZ $\mathbf{10^{DMAc}}$ (5.2 mg, 0.5 mol% BP-PTZ)	24 h	24
7	0.2	PEEK-PTZ **10'** (9.9 mg, 0.5 mol% BP-PTZ)	24 h	21
8-1	0.1	PEEK-PTZ **10'** (5.1 mg, 0.5 mol% BP-PTZ)	72 h	80
8-2	0.1	PEEK-PTZ **10'** (5.1 mg, 0.5 mol% BP-PTZ)	72 h	82
8-3	0.1	PEEK-PTZ **10'** (5.1 mg, 0.5 mol% BP-PTZ)	72 h	82
9	0.1	*N*-Ph-benzoPTZ (5 mol%)	24 h	61
10	0.1	PEEK-benzoPTZ **11** (5.1 mg, 0.5 mol% BP-benzoPTZ)	24 h	25
11[b]	0.1	*N*-Ph-benzoPTZ (5 mol%)	24 h	21
12[b]	0.1	PEEK-benzoPTZ **11** (5.1 mg, 0.5 mol% BP-benzoPTZ)	24 h	0

[a] A mixture of **13'** (0.1 mmol), catalyst, NEt*i*Pr$_2$ (0.5 mmol), HCO$_2$H (0.5 mmol), and acetonitrile (1.0 mL) was stirred at 30 °C under UV irradiation using LED light purchased from Aldrich (catalog No. ALDRP2, 400-410 nm) at a distance of 1.5-2.0 cm (Fig. S18). Yields were determined by GC. [b] UV irradiation using LED blue light purchased from Aldrich (catalog No. ALDKIT001, 435-445 nm) at a distance of 1.5-2.0 cm.

The dehydrogenation of 4-bromobenzonitrile (**13'**) proceeded to observe benzonitrile (**14**) observed when using 5 mol% of the standard catalyst 10-phenylphenothiazine (*N*-Ph-PTZ). Meanwhile, using about 5 mg of **10** containing ca. 0.5 mol% of BP-PTZ afforded **14** in about 30% yield. The yield of **14** increased to about 80% when using about 5 mg of **10'** for a prolonged reaction time of 72 h. The same catalytic activity was observed for $\mathbf{10^{DMAc}}$ and **11**.

Table S3 | Catalytic dehalogenation of 4-chlorobenzonitrile (13).[a]

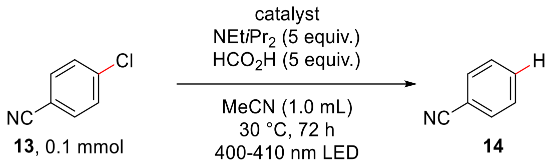

Entry	catalyst	**14** (%)
1	-	1
2	*N*-Ph-PTZ (5 mol%)	89
3	**6** (5 mol%), 24 h	75
4	PEEK powder **6** (4.9 mg, 17 mol% PEEK)	3
5	PEEK-PTZ **10** (5.1 mg, 0.5 mol% BP-PTZ)	4
6-1	PEEK-PTZ **10** (4.9 mg, 0.5 mol% BP-PTZ)	92
6-2	PEEK-PTZ **10** (5.1 mg, 0.5 mol% BP-PTZ)	91
6-3	PEEK-PTZ **10** (4.9 mg, 0.5 mol% BP-PTZ)	87
6-4	PEEK-PTZ **10** (5.0 mg, 0.5 mol% BP-PTZ)	89

[a] A mixture of **13** (0.1 mmol), catalyst, NEt*i*Pr$_2$ (0.5 mmol), HCO_2H (0.5 mmol), and acetonitrile (1.0 mL) was stirred at 30 °C under UV irradiation using LED light purchased from Aldrich (catalog No. ALDRP2, 400-410 nm) at a distance of 0.1 cm for Entries 1,3,4,6 (Fig. S23) and 1.5-2.0 cm for Entries 2 and 5. Yields were determined by GC and ^{1}H NMR spectroscopy.

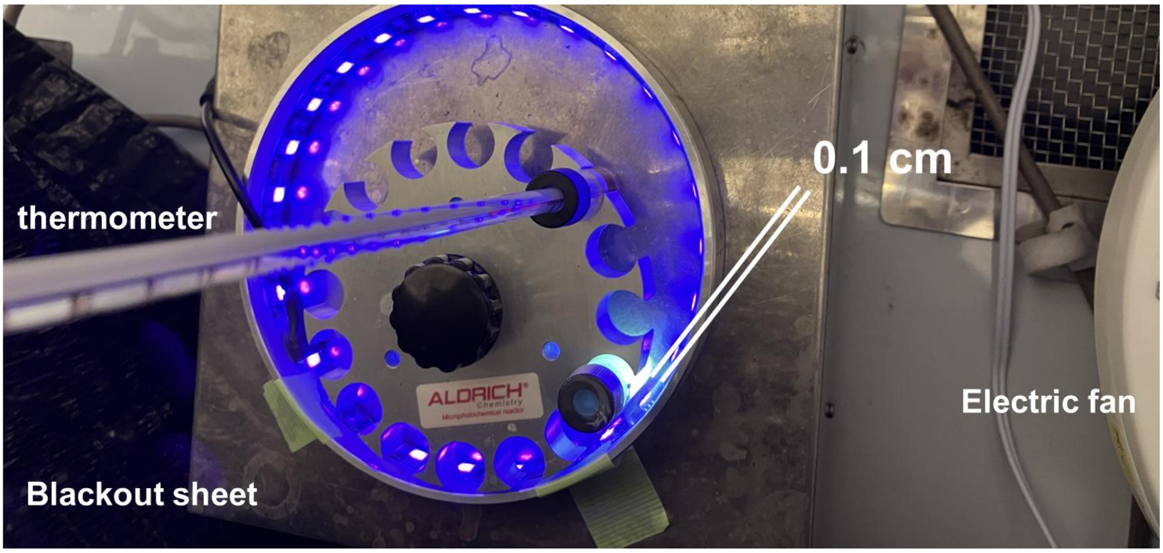

Fig. S23 | Reaction using photoredox catalysts under photo irradiation by purple LED light ring for Aldrich® micro photochemical reactor (spectral range 400-410 nm) at a distance of 0.1 cm.

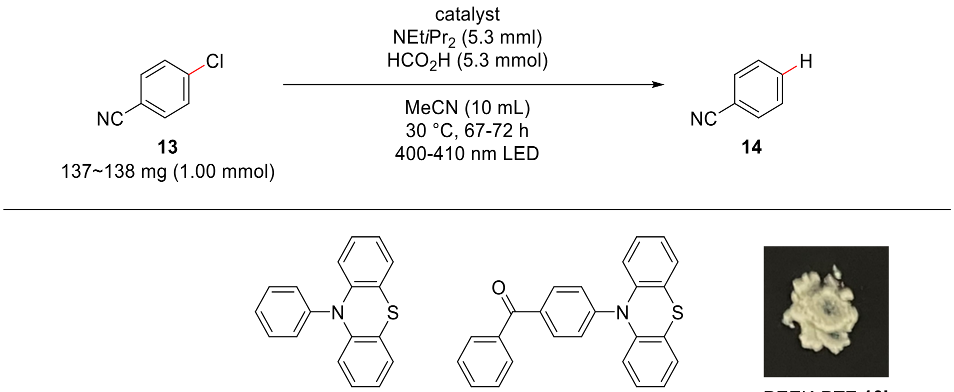

no catalyst	*N*-Ph-PTZ (0.5 mol%)	**9** (0.5 mol%)	PEEK-PTZ **10'** (50.9 mg, 0.6 mol% BPZ)
reaction time, 68 h	reaction time, 67 h	reaction time, 67 h	reaction time, 72 h
yield of **14**, <1%	yield of **14**, 67%	yield of **14**, 57%	yield of **14**, 67%

Fig. S24 | Dehalogenation of **13** (1.0 mmol) using a catalyst: *N*-Ph-PTZ (0. 54 mol%), **9** (0.50 mol%), or PEEK-PTZ **10’** (0.56 mol% BP-PTZ unit), ethyldiisopropylamine (5.3 equiv.), formic acid (5.3 equiv.), and acetonitrile (10 mL) in a 12 mL vial at 30 °C for 67-72 h under UV irradiation using LED light purchased from Aldrich (catalog No. ALDRP2, 400-410 nm) at a distance of 0.1 cm. Based on the yield of **14** using PEEK-PTZ powder **10’**, the reaction rate decreased maybe because the light absorption efficiency declined due to the increase in solution volume.

As shown above, PEEK-PTZ powder showed catalytic activity for the dehalogenation of 4-chlorobenzonitrile. At first glance, this powder seems that this powder showed different catalytic activity from PTH@SWNT developed by Blanco and Alemán in the dehalogenation reaction.[S14] However, we mentioned that *this comparison is unfair and difficult*. This is because Blanco and Alemán chose 420 nm LED source which is different wavelength.

Compared with the reaction based on 0.1 mmol of **13** shown in Table S3, the reaction rate based on 1.0 mmol of **13** (tenfold amount) decreased maybe because the light absorption efficiency declined due to the increase in solution volume. Under these 1.0 mmol scale conditions, the reaction rate using PEEK-PTZ **10’** (0.56 mol% BP-PTZ unit) was similar to the case using *N*-Ph-PTZ (0. 54 mol%) and slightly faster than **9** (0.50 mol%).

6. Preliminary degradative surface functionalization of purchased PEEK pellets

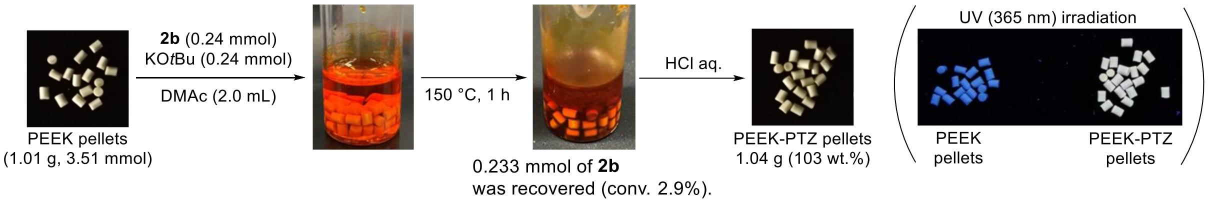

Degradative surface functionalization of PEEK pellets by 10*H*-phenothiazine (2b) and potassium *tert*-butoxide in DMAc solvent. To a mixture of PEEK pellets (1.01 g, 3.51 mmol of PEEK relative to the molecular weight of the monomer), 10*H*-phenothiazine (**2b**) (47.8 mg, 0.240 mmol), and potassium *tert*-butoxide (27.0 mg, 0.241 mmol) was added *N*,*N*-dimethylacetamide (DMAc, 2.0 mL) in a 12 mL vial under argon atmosphere. The resultant mixture was stirred at 150 °C for 1 h. The reaction mixture was cooled to room temperature. The red solution was removed by decantation. The pellets were treated with HCl aq. (2 M, 4.0 mL) and then washed with $CDCl_3$. At that time, removed DMAc and HCl solution was mixed with the $CDCl_3$. The $CDCl_3$ layer was analyzed by ^{1}H NMR spectroscopy, which contained 0.233 mmol of **2b** (2.9% conversion). The resultant pellets were then washed with methanol and acetone. The obtained pellets were dried at 115 °C for 1 h to obtain pale-yellow PEEK-PTZ pellets (1.04 g, 103 wt.% based on used PEEK pellets), which showed emission under 365 nm irradiation.

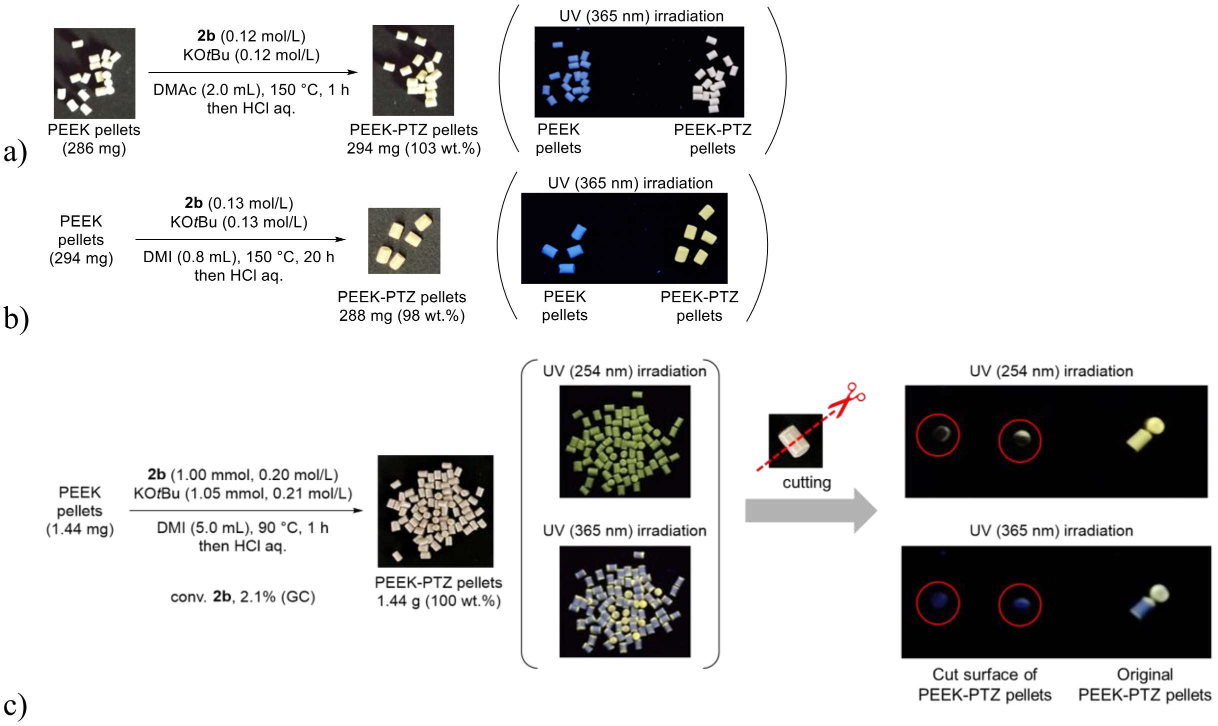

Fig. S25 | Checking the reproducibility of the degradative surface functionalization of PEEK pellets and its emission under UV irradiation. **a** Small scale reaction of pellets with **2b** and KO*t*Bu (0.12 mol/L) in DMAc at 150 °C for 1 h. **b** Reaction with **2b** and KO*t*Bu (0.13 mol/L) in DMI at 150 °C for 20 h. **c** Reaction with **2b** and KO*t*Bu (0.20 mol/L) in DMI at 90 °C for 1 h. Cut surface of one PEEK-PTZ pellet did not emit under UV irradiation unlike the original surface.

7. Preliminary degradative surface functionalization of purchased PEEK plates

Preliminary reaction of a commercially available pure PEEK plate **A** from Asone (catalog Number: 2-9239-1) with 0.2 mol/L of **2b** and KO*t*Bu in DMI at 150 °C was examined. As a result, yellow light emissive PEEK plates were obtained after 4 h (Fig. S26a). However, the surface structure of the PEEK plates was damaged during the reaction process. In fact, the flat surface of PEEK plate changed to cobblestone-like surface after 1 h and to a granular lumpy surface after 4 h (Fig. S26b). In addition, the surface became brittle, and the weight of the plates decreased. Raman microscopic analyses of **A** and the plates after the reaction for 1 h and 4 h detected only the PEEK signals which became relatively stronger with reaction time. Probably, the facile cleavage of the main chains of PEEK caused the aggregation of the low-molecular-weight surface polymers.

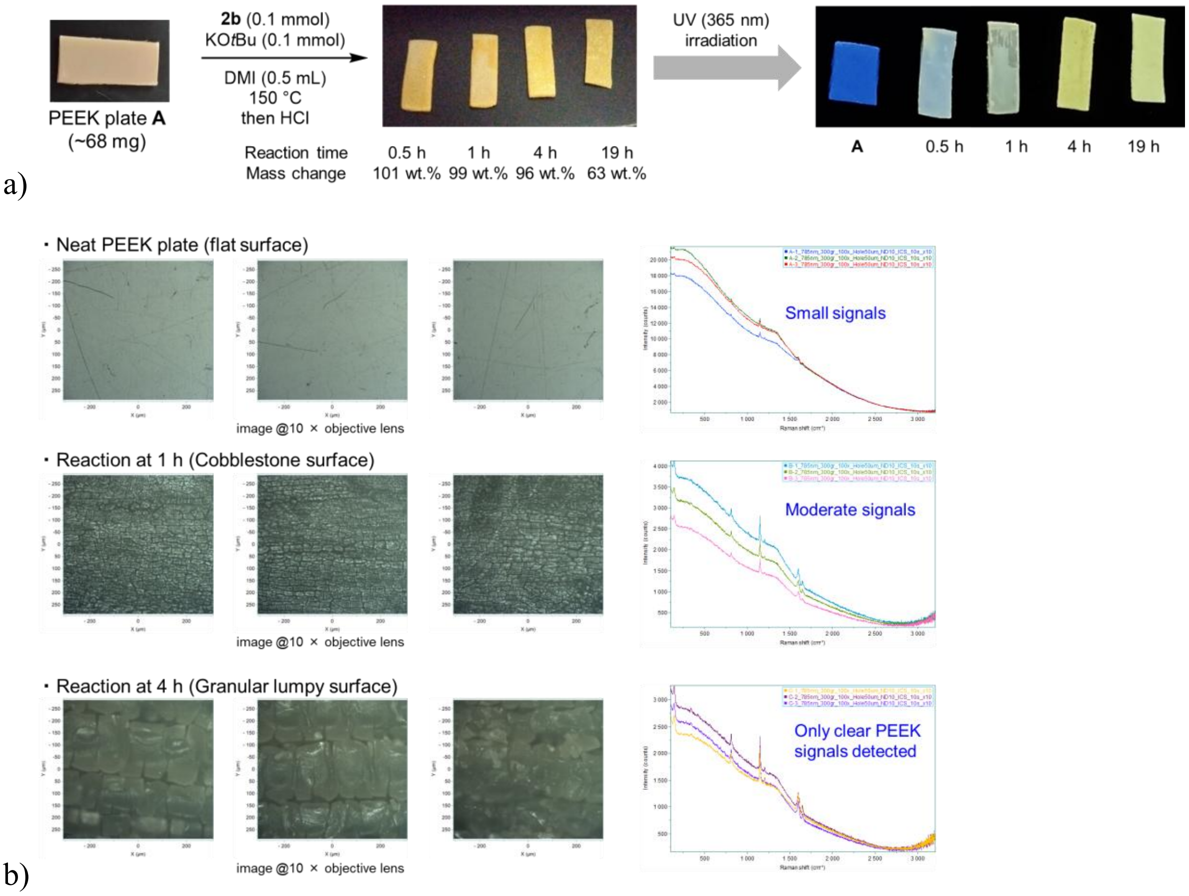

Fig. S26 | Preliminary experiments of the surface reaction of PEEK plate **A** purchased from Asone (catalog Number: 2-9239-1) with 0.2 mol/L of **2b** and KO*t*Bu in DMI at 150 °C. **a** Reaction using PEEK plates **A** with ca. 5 mm × 20 mm × 1 mm for 0.5, 1, 4, and 19 h and produced plates which were irradiated under 365 nm light. **b** Raman microscopic analyses of neat PEEK plate **A** and the plates after the reaction for 1 h and 4 h.

Next, the reaction of various sized PEEK plates **A** with a range of PTZ-based nucleophile concentrations in DMAc at 150 ºC for 1 h was examined (Fig. S27). As a result, 0.10 mol/L of the nucleophile was enough to provide a pale-yellow light emissive PEEK-PTZ plate **C** irradiated by 365 nm light. In this case, the surface structures of the plates were not noticeably damaged. The plate **B** from 0.02 mol/L of the nucleophile emitted little luminescence compared with the original PEEK and **A-heat** heated in DMAc at 150 ºC for 1 h. The surface structures and luminescence of the plate **D** from 0.20 mol/L did not show much difference from **C**.

Observed reflection spectra of functionalized PEEK plates showed that the longest absorption wavelength was shifted to the longer wavelength (ca. 470 nm) compared to PEEK plate **A** and heated **A-heat** (Fig. S28). The photoluminescence spectra irradiated by 360 nm light showed that 0.11 mol/L of nucleophiles in DMAc solvent provided photo-emissive PEEK plate **C** (Fig. S29). The plate **D** obtained using 0.20 mol/L of nucleophiles showed the same results as **C**. The comparison of reflection and photoluminescence spectra of between each plate obtained from the same amounts of reactants showed the reproducibility of this functionalization.

Thus, these experiments demonstrated that more than 0.1 mol/L of **2b** and KO*t*Bu in DMAc were enough to produce light emissive PEEK plates without damaging surface moieties.

a)

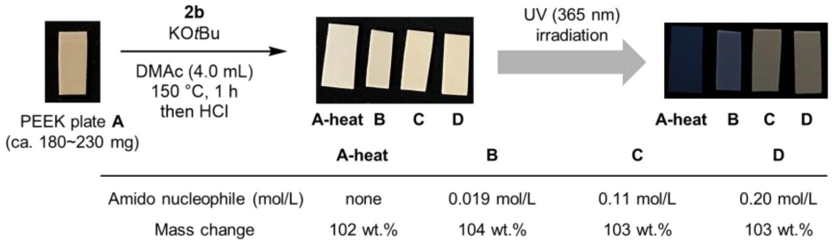

b)

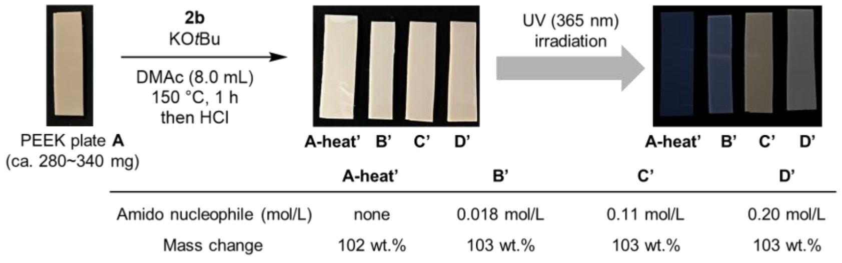

Fig. S27 | Preliminary experiments of the surface reaction of PEEK plate **A** purchased from Asone (catalog Number: 2-9239-1) with a range of concentration of **2b** and KO*t*Bu in DMAc at 150 °C for 1 h. **a** Reaction using PEEK plate **A** with ca. 10 mm × 20 mm × 1 mm. **b** Reaction using PEEK plate **A** with ca. 10 mm × 30 mm × 1 mm.

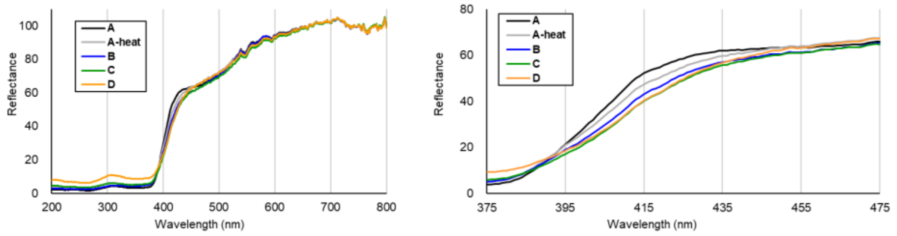

a)

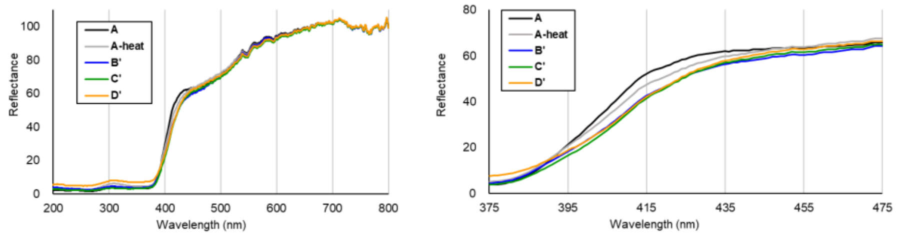

b)

Fig. S28 | Refraction spectra of modified PEEK plates. **a** PEEK plate **A** (black), PEEK plate **A-heat** heated in DMAc at 150 ºC for 1 h (gray), **B** (blue), **C** (green), and **D** (orange). **b** PEEK plate **A** (black), **A-heat** (gray), **B'** (blue), **C'** (green), **D'** (orange).

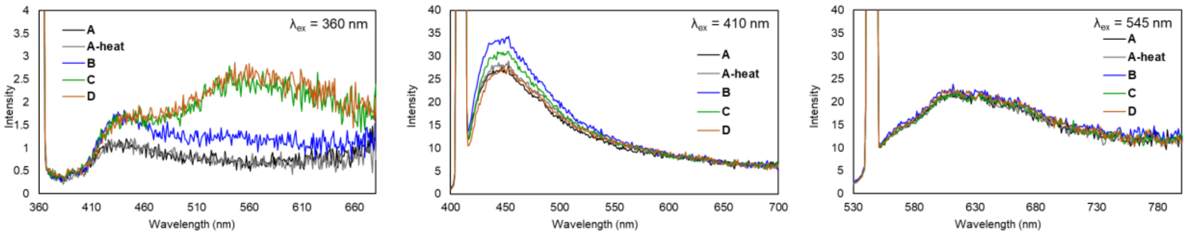

a)

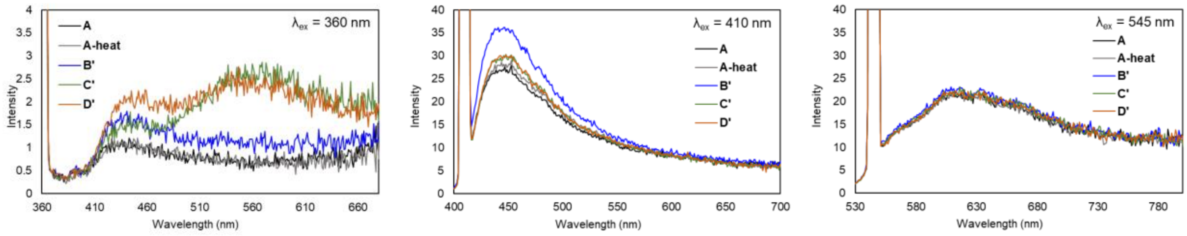

b)

Fig. S29 | Photoluminescence spectra of modified PEEK plates obtained by the irradiation of 360, 410, and 545 nm light. **a** PEEK plate **A** (black), PEEK plate **A-heat** heated in DMAc at 150 ºC for 1 h (gray), **B** (blue), **C** (green), and **D** (orange). **b** PEEK plate **A** (black), **A-heat** (gray), **B'** (blue), **C'** (green), **D'** (orange)

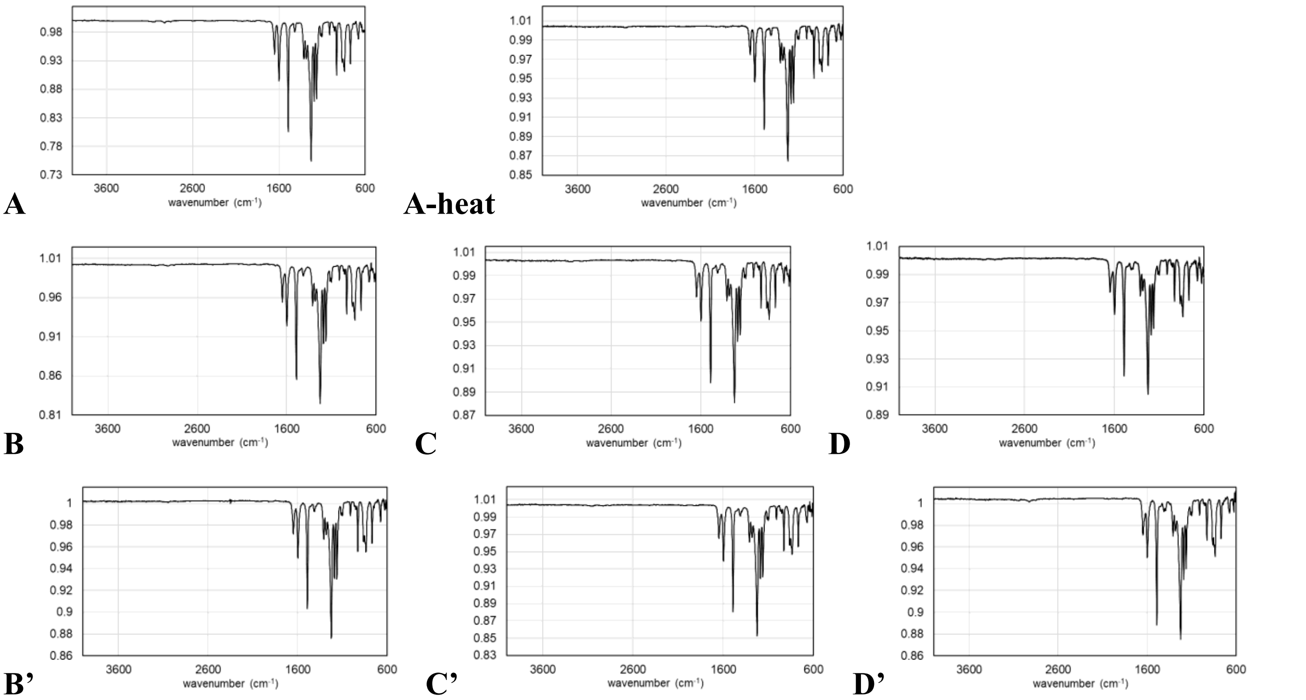

Fig. S30 | ATR-FTIR spectra of **A**, **A-heat**, **B**, **C**, **D**, **B’**, **C’**, and **D’**. Degree of crystallinity of PEEK thin plate (area ratios of intensities of the peaks between 1310 cm^{-1} and 1280 cm^{-1} in ATR-FTIR spectroscopies), **A**: 1.65, **A-heat**: 1.66, **B**: 1.85, **C**: 1.92, **D**: 2.02, **B’**: 1.76, **C’**: 1.82, **D’**: 1.98.

Degradative surface functionalization of **A** was attempted with **2b** and KO*t*Bu (0.20 mol/L) in DMI under mile conditions: 90 °C for 1 h. As a result, weak photo emissive PEEK-PTZ plates **E** were obtained under UV (254 nm and 365 nm) irradiation. Obviously, side parts of **A** were well-functionalized. One plate was cut and the resulting cut surface and original side parts were irradiated by UV light. As a result, the cut surface did not emit, showing that this degradative functionalization occurred on the surface (Fig. S31).

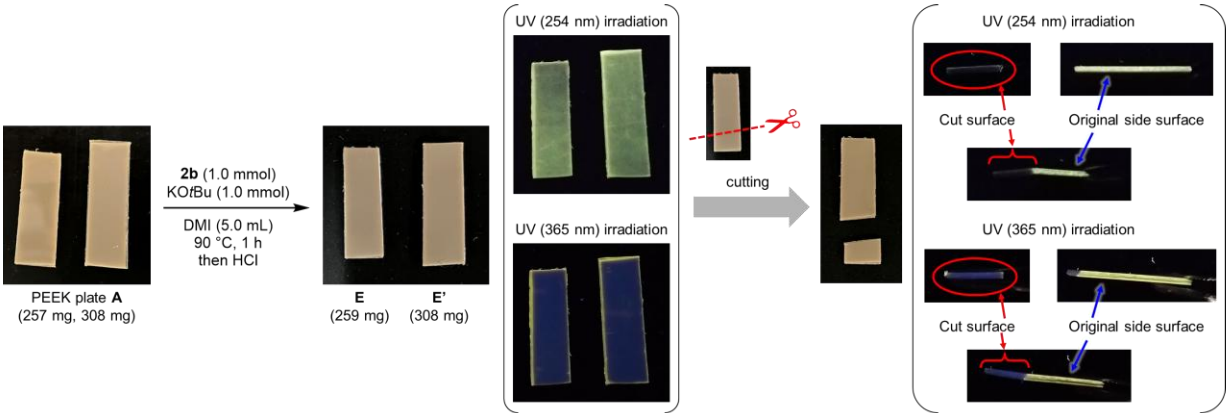

Fig. S31 | Degradative surface functionalization of PEEK plates **A** with **2b** and KO*t*Bu (0.20 mol/L) in DMI at 90 °C for 1 h to form PEEK-PTZ plates **E**. Whole parts of **E** show yellow emission under 254 nm light irradiation whereas edge parts emit under 365 nm light irradiation. Cut surface of one PEEK-PTZ pellet did not emit under UV irradiation unlike the original surface.

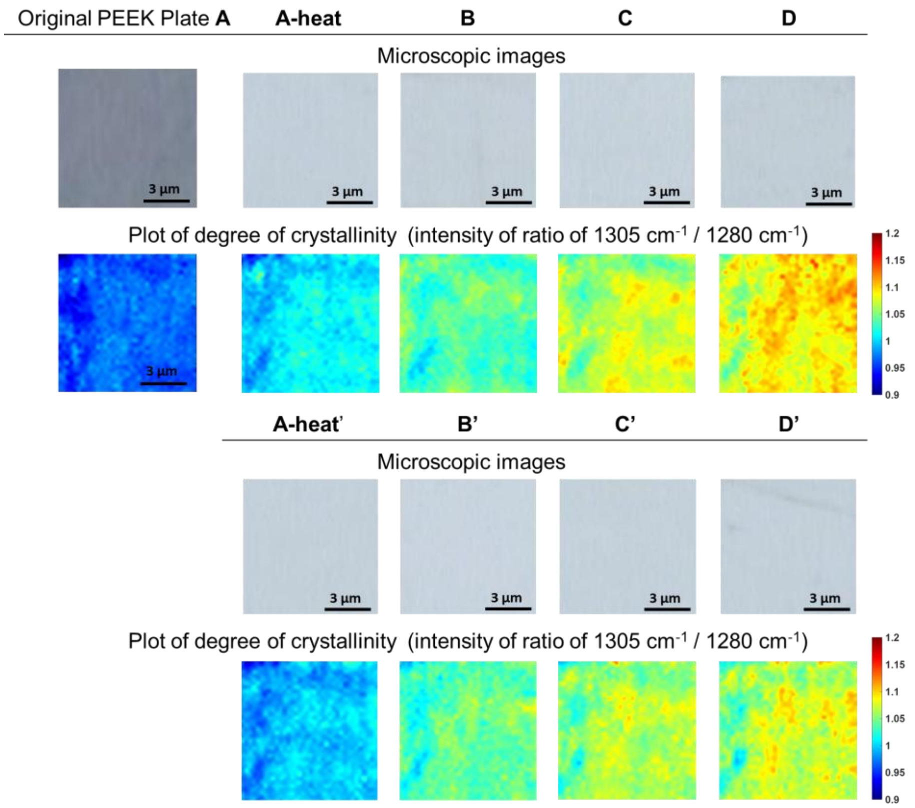

Fig. S32 | Microscopic images and FTIR microscopic analyses of PEEK plates **A**, **A-heat**, **B**, **C**, **D**, **A-heat'**, **B'**, **C'**, and **D'**.

Microscopic images showed that functionalized PEEK plates were not damaged physically. FTIR microscopic images, based on the intensity ratio of 1305 cm^{-1} to 1280 cm^{-1}, showed that heating of PEEK in DMAc at 150 ºC for 1 h enhanced the degree of crystallinity (Fig. S32). However, treatment of PEEK plate with **2b** and KO*t*Bu resulted in more crystallinity, and the degree of crystallinity was higher at higher concentrations of the nucleophile. The trend of the results obtained from microscopic FTIR analyses is in good agreement with the results from ATR-FTIR spectroscopy shown in Fig. S30.

8. Degradative surface functionalization of PEEK plates made of 3D printer

8-1. Procedures

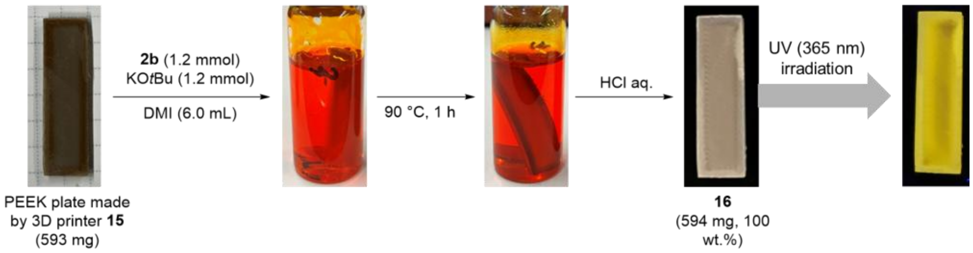

Degradative surface functionalization of PEEK plates using 10*H*-phenothiazine (2b) and KO*t*Bu in DMI solvent at 90 °C. PEEK plate **15** with dimensions of ca. 10 mm × 30 mm × 2 mm (593 mg), which was fabricated using a 3D printer, was placed in a 12 mL vial, and 10*H*-phenothiazine (**2b**) (239 mg, 1.20 mmol), KO*t*Bu (135 mg, 1.20 mmol), DMI (6.0 mL), and a magnetic stirring bar were added under argon atmosphere. The mixture was stirred at 90 °C for 1 h and then cooled to room temperature. The red solution was removed by decantation, and the plate was treated with water and HCl aq. (2 M, 1.0 mL) and then washed with water, methanol, and acetone. The obtained plate was dried at 115 °C for 1 h to provide **16** (594 mg, 100 wt.% based on the PEEK plate used) which was irradiated by 365 nm light to show yellow emission. Of note, the surface structure of **16** was not damaged noticeably during the degradative functionalization.

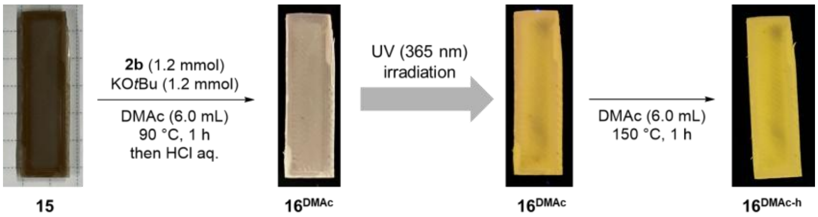

Degradative surface functionalization of PEEK plates using 10*H*-phenothiazine (2b) and KO*t*Bu in DMAc solvent at 90 °C. PEEK plate **15** with dimensions of ca. 10 mm × 30 mm × 2 mm (598 mg), which was fabricated using a 3D printer, was placed in a 12 mL vial, and 10*H*-phenothiazine (**2b**) (239 mg, 1.20 mmol), KO*t*Bu (135 mg, 1.20 mmol), DMAc (6.0 mL), and a magnetic stirring bar were added under argon atmosphere. The mixture was stirred at 90 °C for 1 h and then cooled to room temperature. The red solution was removed by decantation, and the plate was treated with water and HCl aq. (2 M, 1.0 mL) and then washed with water, methanol, and acetone. The obtained plate was dried at 115 °C for 1 h to provide $\mathbf{16^{DMAc}}$ (603 mg, 101 wt.% based on the PEEK plate used) which was irradiated by 365 nm light to show yellow emission. The surface structure of $\mathbf{16^{DMAc}}$ was not damaged noticeably.

Then, $\mathbf{16^{DMAc}}$ was dipped in DMAc (6.0 mL) and heated at 150 °C for 1 h. After washing with acetone and drying at 115 °C for 20 min, the plate $\mathbf{16^{DMAc\text{-}h}}$ was irradiated by 365 nm light to show yellow emission.

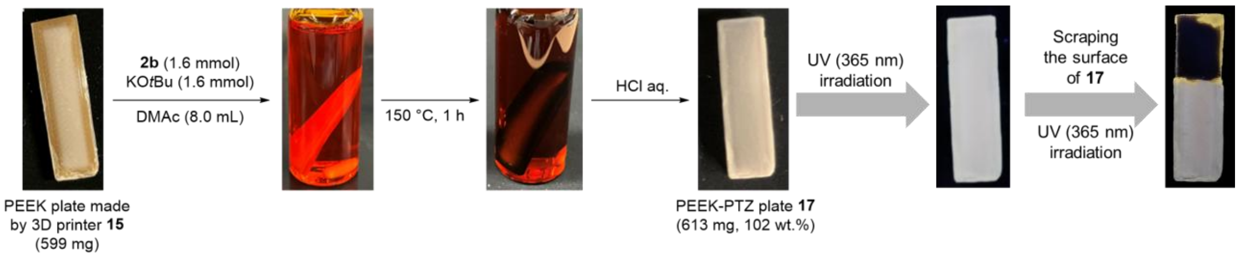

Degradative surface functionalization of PEEK plates by 10*H*-phenothiazine (2b) and potassium *tert*-butoxide in DMAc solvent at 150 °C. PEEK plate **15** with dimensions of ca. 10 mm × 30 mm × 2 mm (599 mg) was placed in a 12 mL vial. 10*H*-Phenothiazine (**2b**) (316 mg, 1.59 mmol), potassium *tert*-butoxide (181 mg, 1.61 mmol), DMAc (8.0 mL), and a magnetic stirring bar were added under argon atmosphere. At that time, the plate **15** was immersed in the solution. This mixture was stirred at 150 °C for 1 h and cooled to room temperature. The red solution was removed by decantation, and the plate was treated with water and HCl aq. (2 M, 1.0 mL) and then washed with water, methanol, and acetone. The obtained plate was dried at 115 °C for 1 h to provide **17** (613 mg, 102 wt.% based on used PEEK plate). The surface structure of **17** was not damaged noticeably during the degradative functionalization. **17** showed a white emission under 365 nm photo irradiation. After the surface was scrapped, the corresponding part did not show the emission, and the generated edge part emitted a yellow light.

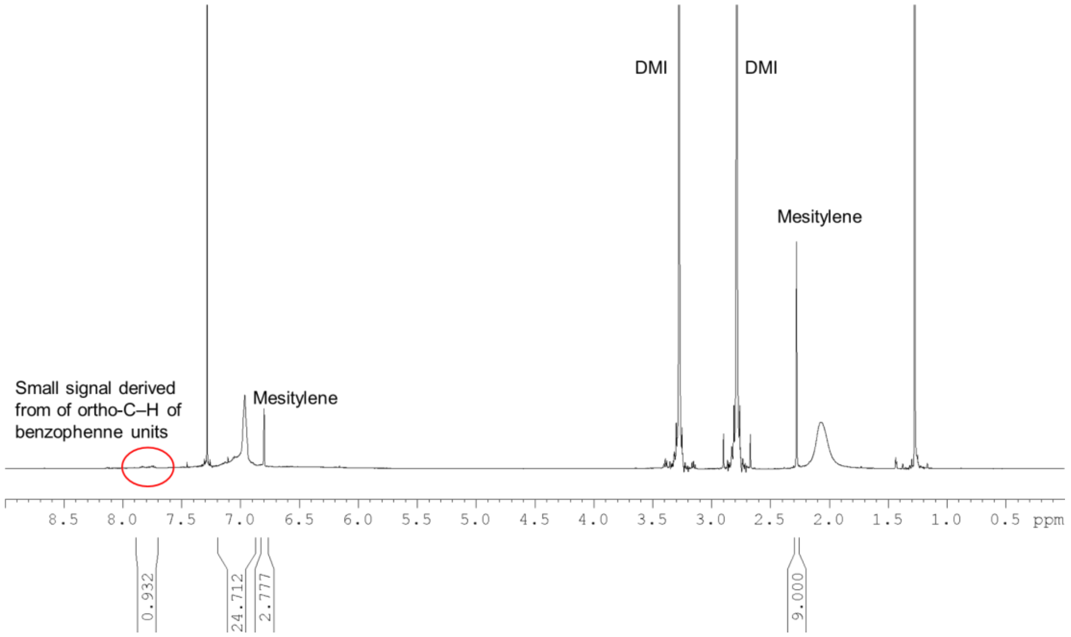

Fig. S33 | NMR spectroscopic analysis of the soluble crude mixture from the reaction of PEEK powder **6** with phenothiazine (**2b**) (2.5 equiv.) and KO*t*Bu (2.5 equiv.) in DMI solvent at 90 °C for 20 h. After the treatment with 1 M HCl aq., soluble organic components were extracted with $CDCl_3$ and analyzed using mesitylene as an intermediate.

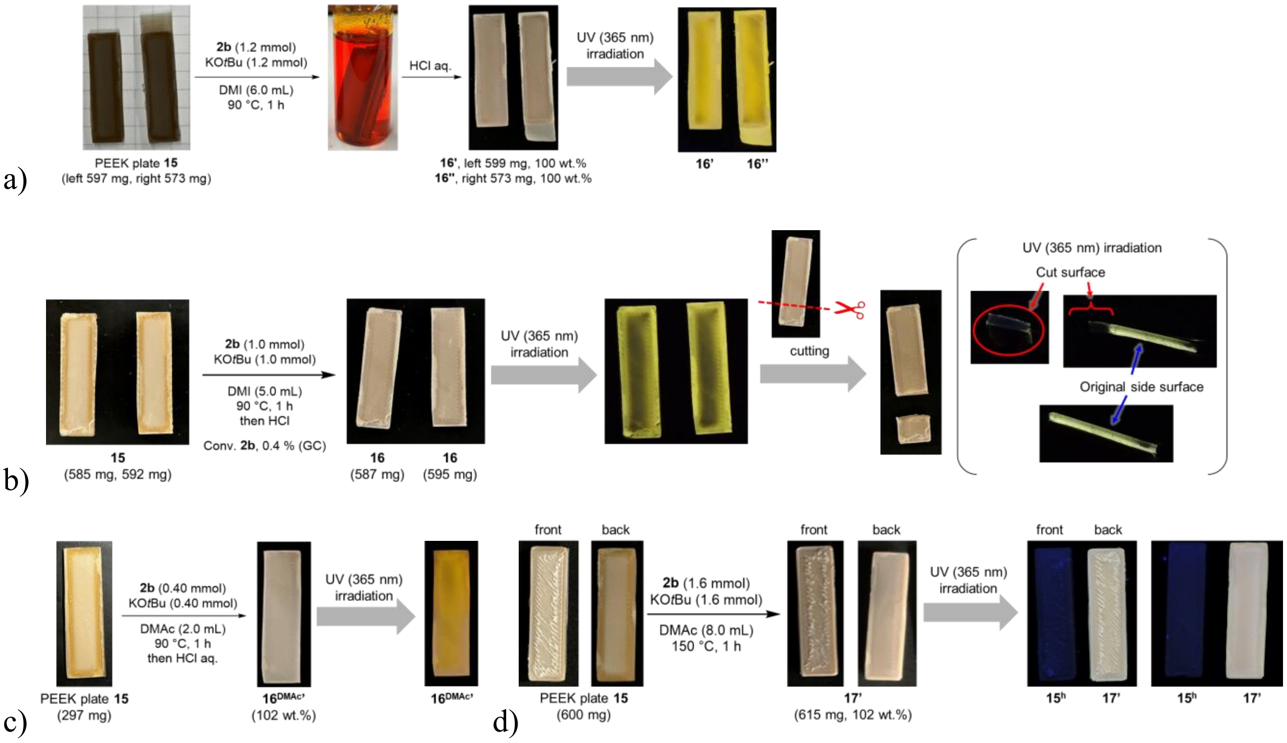

Fig. S34 | Checking the reproducibility. The production of the yellow light emissive PEEK plates using **a, b** DMI or **c** DMAc solvent at 90 °C. **d** The production of the white light emissive PEEK plate **17'** using DMAc solvent at 150 °C. In **b**, one PEEK-PTZ plate **16** was cut and generated cut surface did not emit under 365 nm light irradiation unlike the original surface.

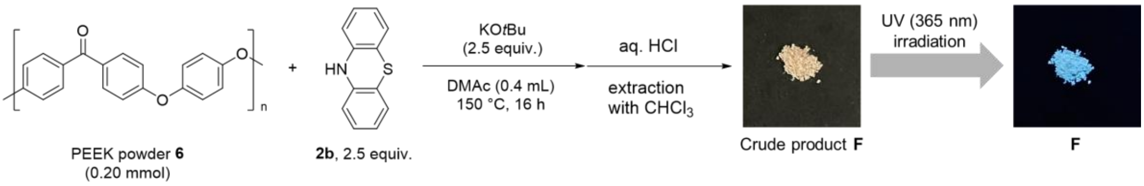

Fig. S35 | Reaction of PEEK powder **6** with **2b** and KO*t*Bu in DMAc at 150 °C for 16 h to generate complex mixture **F** which emitted blue light under 365 nm irradiation.

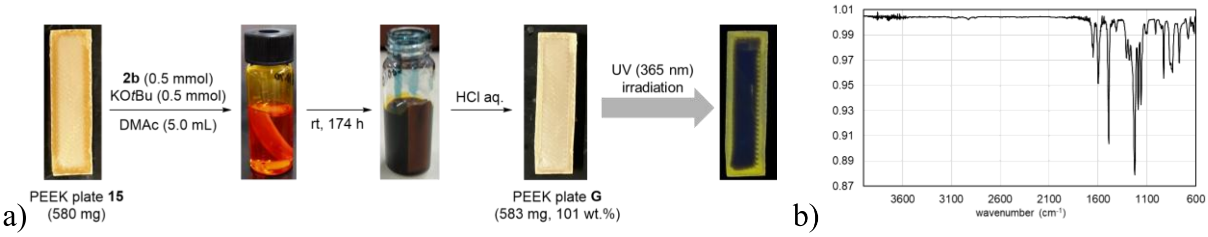

Fig. S36 | Reaction of **15** with **2b** and KOtBu in DMAc at room temperature. **a** Reaction scheme: PEEK plate **15** with dimensions of ca. 10 mm × 30 mm × 2 mm (580 mg), which was fabricated using a 3D printer was treated with **2b** (99.4 mg, 0.50 mmol, 0.1 mol/L) and KO*t*Bu (57.9 mg, 0.516 mmol, 0.1 mol/L) in DMAc (5.0 mL) at room temperature for 174 h. After the treatment with 1 M HCl aq., the treated plate was washed with water, methanol, and acetone, and dried at 150 °C for 30 min. Obtained plate **G** (583 mg) showed yellow emission at the edge moiety under 365 nm photo irradiation. **b** ATR-FTIR spectra of the center part of **G**. Degree of crystallinity (area ratios of intensities of the peaks between 1310 cm^{-1} derived from crystalline moiety and 1280 cm^{-1} derived from crystalline and amorphous moieties)[S10] of center part: 1.53.

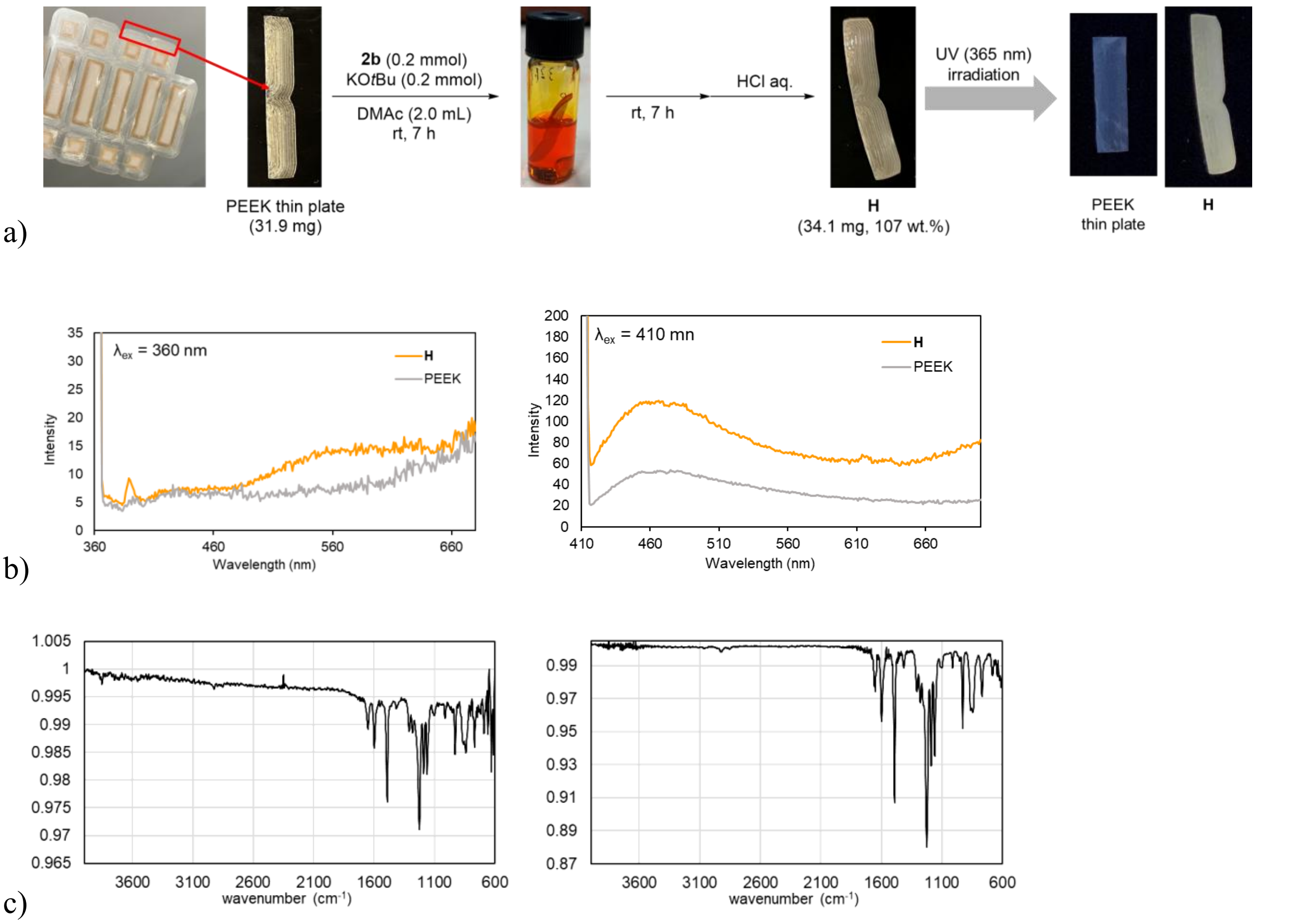

Fig. S37 | Reaction of PEEK thin plate with **2b** and KOtBu in DMAc at room temperature. **a** Reaction scheme: PEEK thin plate (thickness: 0.1 mm) (31.9 mg) which is runner parts of prepared PEEK plate by 3D printer, was treated with **2b** (39.8 mg, 0.20 mmol, 0.1 mol/L) and KO*t*Bu (24.4 mg, 0.217 mmol, 0.1 mol/L) in DMAc (2.0 mL) at room temperature for 7 h. After the treatment with 1 M HCl aq. treated thin plate was washed with water, methanol, and acetone, and dried at room temperature. Prepared thin plate **H** (34.1 mg) showed white emission under 365 nm photo irradiation. **b** Photoluminescence spectra of **H** and PEEK thin plate by the irradiation of 360 nm and 410 nm light. **c** ATR-FTIR spectra of the center part of **H** (left) and PEEK thin plate (right). Degree of crystallinity of PEEK thin plate (area ratios of intensities of the peaks between 1310 cm^{-1} and 1280 cm^{-1} in ATR-FTIR spectroscopies): 1.19 and **H**: 1.74.

The treatment of PEEK thin plate with **2b** was examined at room temperature for 7 h under same concentration as Fig. S36, and light emissive plate was obtained (Fig. S37). The degree of crystallinity of the thin plate was 1.19 which was lower than the center of **15** (1.42) (Fig. S40), indicating that the low crystallinity affects the reaction rate. Moreover, the degree of crystallinity of **H** was 1.74, which is higher than the PEEK thin plate.

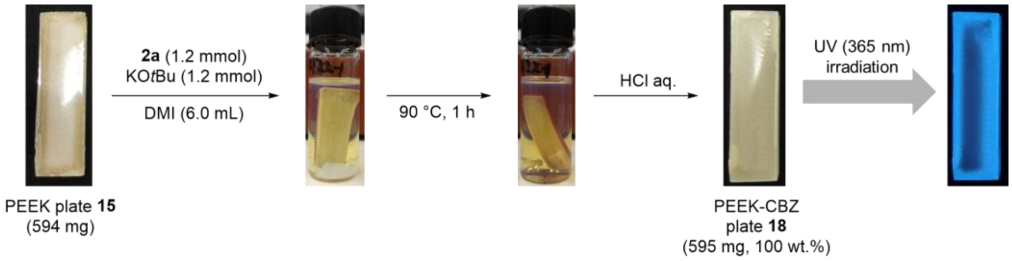

Degradative surface functionalization of PEEK plates by 3,6-di-*tert*-butylcarbazole (2a) and potassium *tert*-butoxide in DMI solvent. PEEK plate **15** with dimensions of ca. 10 mm × 30 mm × 2 mm (594 mg), which was fabricated using a 3D printer, was placed in a 12 mL vial. 3,6-Di-*tert*-butylcarbazole (**2a**) (336 mg, 1.2 mmol), potassium *tert*-butoxide (138 mg, 1.23 mmol), DMI (6.0 mL), and a magnetic stirring bar were added to this vial under argon atmosphere. This mixture was stirred at 90 °C for 1 h and cooled to room temperature. The reaction solution was removed by decantation, and the plate was treated with water and HCl aq. (2 M, 1.0 mL) and then washed with water, methanol, and acetone. The obtained plate was dried at 115 °C for 1 h to provide **18** (595 mg, 100 wt.% based on the PEEK plate used) which showed a blue emission under 365 nm UV irradiation. The surface structure of **18** was not damaged noticeably.

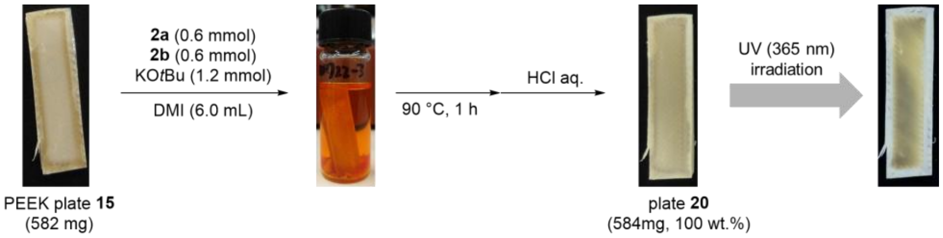

Degradative surface functionalization of PEEK plates by 3,6-di-*tert*-butylcarbazole (2a), 10*H*-phenothiazine (2b), and potassium *tert*-butoxide in DMI solvent. PEEK plate **15** with dimensions of ca. 10 mm × 30 mm × 2 mm (582 mg), which was fabricated using a 3D printer, was placed in a 12 mL vial. 3,6-Di-*tert*-butylcarbazole (**2a**) (168 mg, 0.60 mmol), 10*H*-phenothiazine (**2b**) (119 mg, 0.60 mmol), potassium *tert*-butoxide (138 mg, 1.2 mmol), DMI (6.0 mL), and a magnetic stirring bar were added to this vial under argon atmosphere. This mixture was stirred at 90 °C for 1 h and cooled to room temperature. The reaction solution was removed by decantation, and the plate was treated with water and HCl aq. (2 M, 1.0 mL) and then washed with water, methanol, and acetone. The obtained plate was dried at 115 °C for 1 h to provide **20** (584 mg, 100 wt.% based on used PEEK plate) which showed a pale blue-yellow emission under 365 nm UV irradiation.

*The plates **19** and **21** were prepared according to the above procedure.*

7-2. Examination of solvents for degradative surface functionalization.

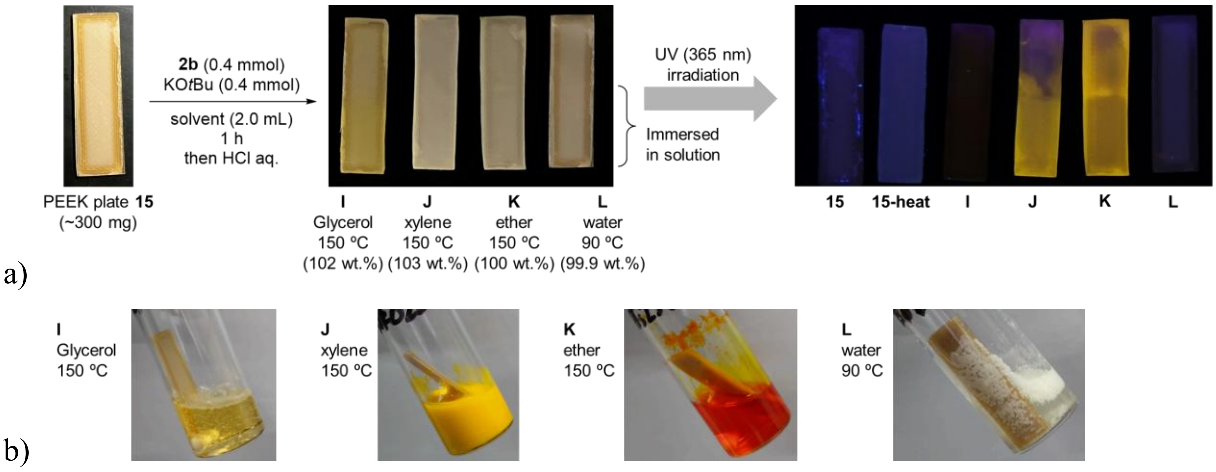

Fig. S38 | Examination of degradative surface functionalization of PEEK plate **15** in various solvents. **a** Reaction scheme: PEEK plate made of the 3D printer **15** with dimensions of ca. 10 mm × 30 mm × 1 mm, was treated with **2b** (0.4 mmol) and KO*t*Bu (0.4 mmol) in solvent (2.0 mL) at 90 °C or 150 °C for 1 h. After the treatment with HCl aq. and washing with HCl aq., methanol, and acetone, obtained plates **I** (from glycerol at 150 °C), **J** (from xylene at 150 °C), **K** (from di(ethoxyethyl)ether at 150 °C), and **L** (from water at 90 °C) were irradiated under 365 nm light. As a result, plates **J** and **K** showed observable yellow emission whereas plate **I** showed slightly yellow emission. **b** Photo images after the reaction mixtures.

The results suggested that many organic solvents such as amides, ethers, alcohols, and arenes were utilized for the surface degradation (Fig. S38). It is assumed that even a small amount of reactant dissolution can undergo the surface degradation. However, glycerol showed less activity probably because of trapping bases. No reaction was observed when using water.

7-3. Analyses of functionalized PEEK plates.

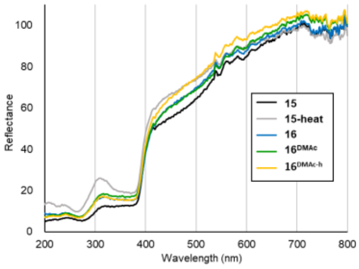

a)

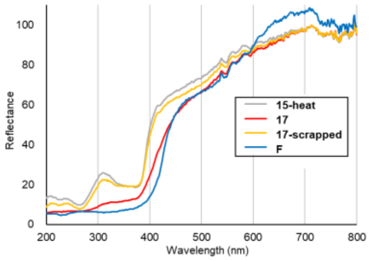

b)

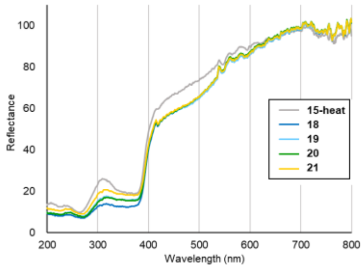

c)

Fig. S39 | Refraction spectra of modified PEEK plates. **a** PEEK plate **15** (black), PEEK plate **15-heat** heated in DMAc at 150 ºC for 1 h (gray), **16** (blue), **16$^{\mathbf{DMAc}}$** (green), and **16$^{\mathbf{DMAc\text{-}h}}$** (yellow). **b 15-heat** (gray), **17** (red), **17**-scrapped (yellow), and **F** (blue). **c 15-heat** (gray), **18** (blue), **19** (pale blue), **20** (green), and **21** (yellow).

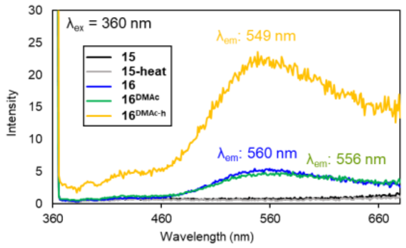

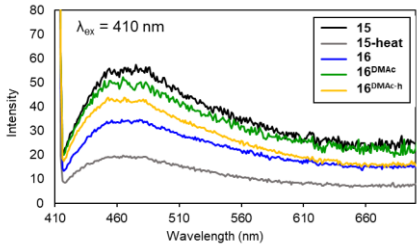

a)

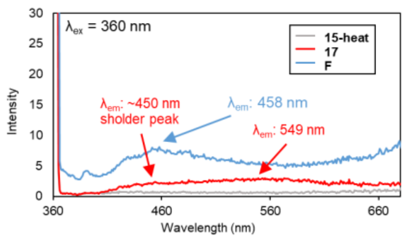

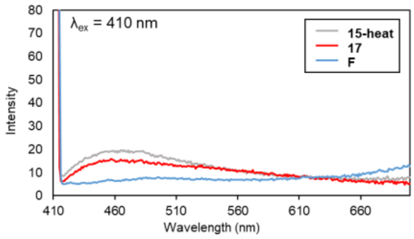

b)

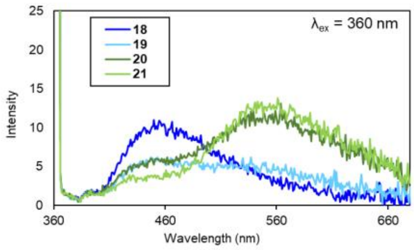

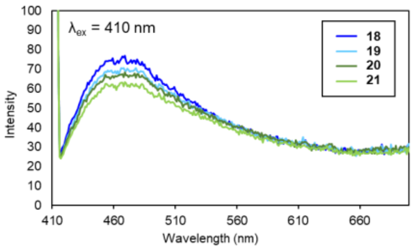

c)

Fig. S40 | Photoluminescence spectra of modified PEEK plates obtained by the irradiation of 360 and 410 nm light. **a** PEEK plate **15** (black), PEEK plate **15-heat** heated in DMAc at 150 ºC for 1 h (gray), **16** (blue), **16$^{\mathbf{DMAc}}$** (green), and **16$^{\mathbf{DMAc\text{-}h}}$** (yellow). **b 15-heat** (gray), **17** (red), and **F** (blue). **c 18** (blue), **19** (pale blue), **20** (green), and **21** (pale green).

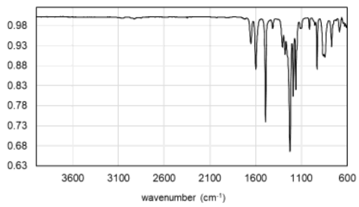

15

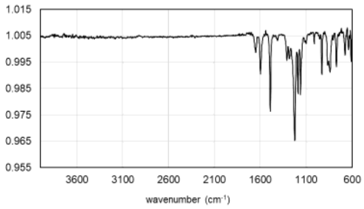

15-heat

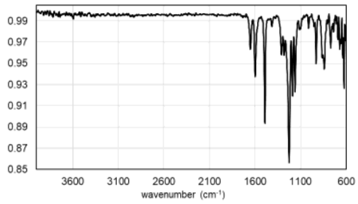

16

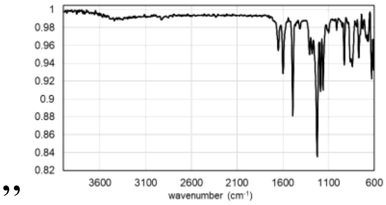

16’’

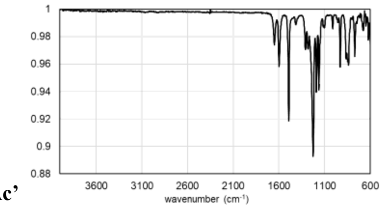

16$^{\mathbf{DMAc'}}$

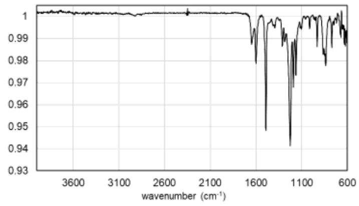

17

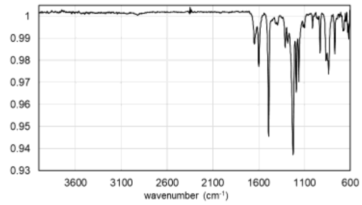

17’

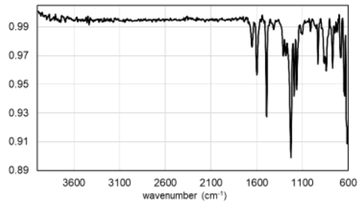

18

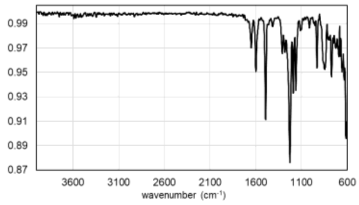

19

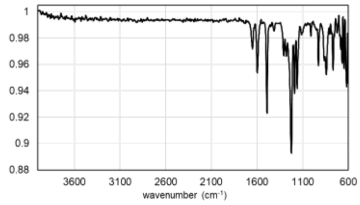

20

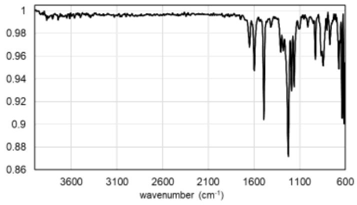

21

Fig. S41 | ATR-FTIR spectra of PEEK plate **15**, PEEK plate **15-heat** heated in DMAc at 150 °C for 1 h, and functionalized PEEK plates, center part of **16**, **16’’**, **16$^{\mathbf{DMAc'}}$**, **17**, **17’**, **18**, **19**, **20**, and **21**.

IR spectroscopic results of PEEK plates looked the same as PEEK powder **6**. Degree of crystallinity (area ratios of intensities of the peaks between 1310 cm^{-1} derived from crystalline moiety and 1280 cm^{-1} derived from crystalline and amorphous moieties)[S10] of **15**: 1.42, **15-heat**: 1.55, **16**: 1.82, **16”**: 1.55, **17**: 2.10, **17’**: 2.04, **18**: 1.55, **19**: 1.84, **20**: 1.60, and **21**: 1.70.

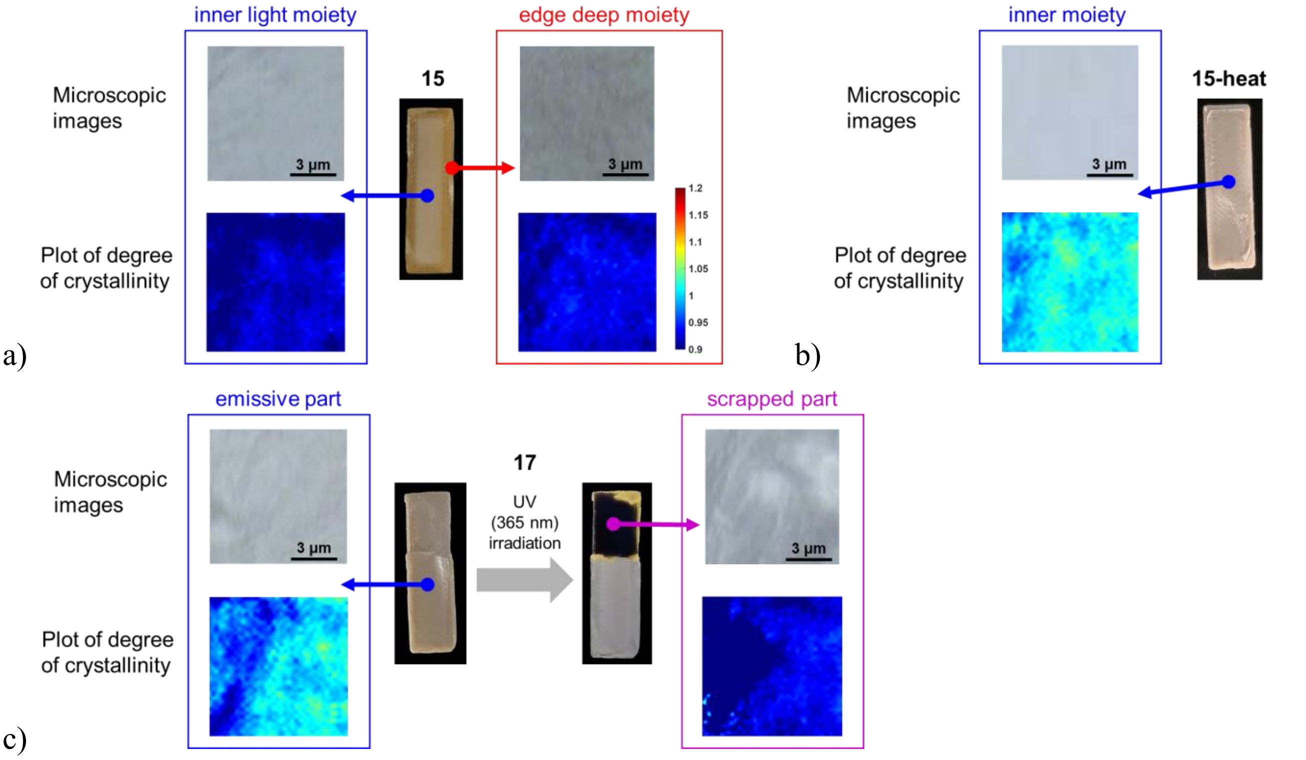

Fig. S42 | Microscopic images and FTIR microscopic analyses of PEEK plates **15**, **15-heat**, and **17**. FTIR microscopic images showed the degree of crystallinity determined by the intensity ratio between the signal of 1305 cm^{-1} and 1280 cm^{-1}. **a** Inner light moiety and edge deep moiety of **15**. **b** **15-heat**. **c** Original light emissive moiety and scrapped moiety of **17**.

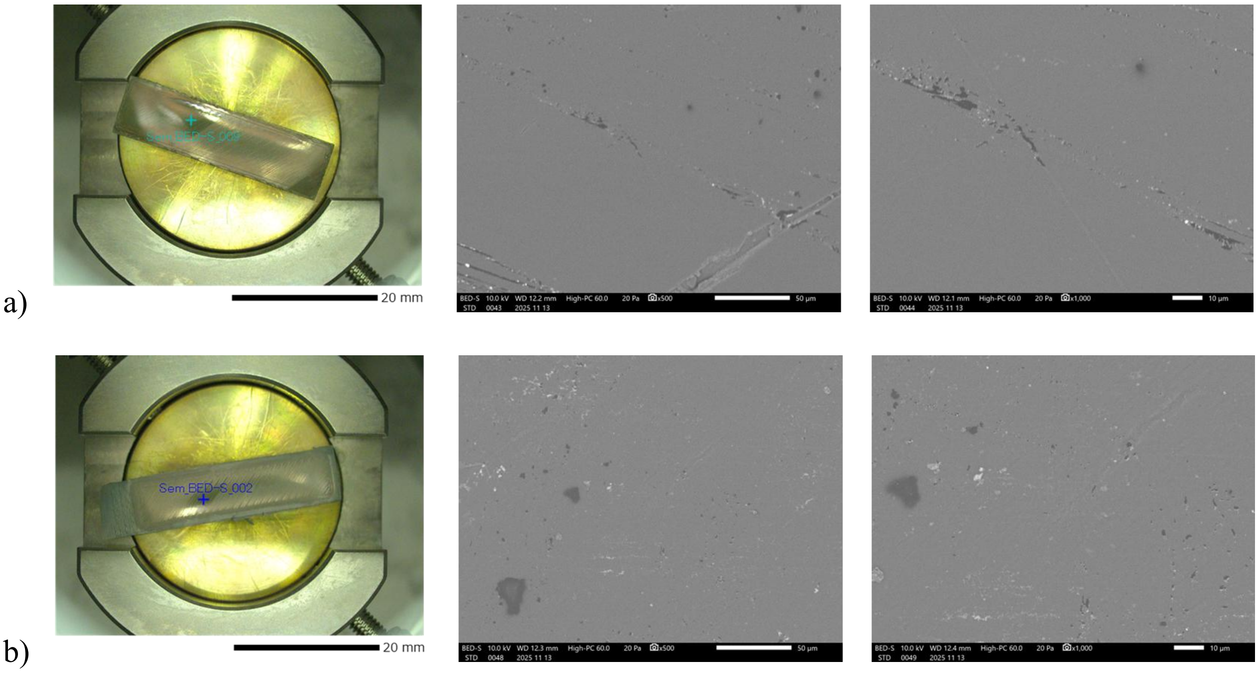

Fig. S43 | a SEM images of prepared PEEK plate **15** (reft: Observation Position (+), middle: ×500, right: ×1,000). **b** SEM images of **16’’** (reft: Observation Position (+), middle: ×500, right: ×1,000).

Microscopic images showed that functionalized PEEK plate **17** was not damaged physically (Fig. S42). The FTIR microscopic image showed that the inner light moiety and the edge moiety of **15** were the same degree of crystallinity. **15-heat** and the original part of **17** showed the same degree of crystalline improvement compared to **15**. The scrapped surface of **17** was the same degree of crystallinity as **15**, demonstrating that the increasing of crystallinity by the functionalization treatment in DMAc occurs only on the surface.

SEM images between prepared PEEK plate **15** and **16’’** also showed that the reaction using **2b** (0.2 mol/L) and KO*t*Bu (0.2 mol/L) in DMI at 90 °C did not damage the surface (Fig. S43).

Table S4 | Summary of degree of crystallinity, emission maxima (λ_{em}), and absolute internal quantum efficiencies (Φ_f) of original PEEK samples and functionalized PEEK products.

PEEK	Degree of crystallinity	360 nm	410 nm	545 nm
		λ_{em} (nm), (Φ_f)	λ_{em} (nm), (Φ_f)	λ_{em} (nm), (Φ_f)
**6**	0.75	424 (<1%)	438 (13%)	- (5%)
**6-heat**	1.86	-	-	-
**10**	1.86	551 (4%)	530 (6%)	- (2%)
**10'**	1.85	553 (4%)	543 (6%)	630 (3%)
**10^{DMAc}**	1.95	551 (1%)	529 (6%)	- (2%)
**10^{DMAcrep}**	1.91	538 (2%)	537 (7%)	- (2%)
**10^{1h}**	1.86	554 (5%)	548 (7%)	- (3%)
**10^{5h}**	1.95	545 (4%)	541 (7%)	- (3%)
**11**	1.75	541 (3%)	538 (6%)	643 (3%)
**12**	2.00	457 (2%)	467 (12%)	- (1%)
**15**	1.42	- (-)	473 (1%)	621 (0.6%)
**15-heat**	1.55	- (-)	465 (2%)	629 (0.7%)
**16**	1.82	560 (<1%)	464 (2%)	N.A.
**16"**	1.55	568 (<1%)	484 (2%)	N.A.
**16^{DMAc}**	N.A.	556 (<1%)	465 (3%)	N.A.
**16$^{DMAc'}$**	1.64	467 (<1%)	479 (2%)	633 (2%)
**17**	2.10	549 (<1%)	457 (1%)	618 (0.7%)
**17'**	2.05	543 (<1%)	451 (1%)	625 (0.6%)
**18**	1.55	450 (<1%)	469 (5%)	N.A.
**19**	1.84	447 (<1%)	478 (4%)	N.A.
**20**	1.60	564 (<1%)	469 (4%)	N.A.
**21**	1.70	561 (<1%)	469 (3%)	N.A.
**A**	1.65	443 (<1%)	453 (2%)	620 (2%)
**A-heat**	1.66	451 (<1%)	453 (2%)	613 (2%)
**B**	1.85	441 (<1%)	453 (3%)	612 (2%)
**C**	1.92	541 (<1%)	452 (3%)	615 (2%)
**D**	2.02	546 (<1%)	448 (2%)	613 (2%)
**B'**	1.76	441 (<1%)	447 (3%)	609 (2%)
**C'**	1.82	569 (<1%)	448 (2%)	616 (2%)
**D'**	1.98	547 (<1%)	448 (3%)	636 (2%)

N.A.: not analyzed.

Absolute internal quantum efficiencies were calculated according to the following equation.

Φ_f = (amount of light emitted) / (amount of light absorbed) = $(S_sE_m - S_bE_m) / (S_bE_x - S_sE_x)$
S_bE_x : excitation peak area of blank spectrum
S_bE_m : fluorescence peak area of blank spectrum
S_sE_x : excitation peak area of sample spectrum
S_sE_m : fluorescence peak area of sample spectrum

Supplementary references

S1) Zhang, Q., Li, J., Shizu, K., Huang, S., Hirata, S., Miyazaki, H., Adachi, C. Design of Efficient Thermally Activated Delayed Fluorescence Materials for Pure Blue Organic Light Emitting Diodes. *J. Am. Chem. Soc*. **134**, 14706−14709 (2012).

S2) Xu, S., Liu, T., Mu, Y., Wang, Y.-F., Chi, Z., Lo, C.-C., Liu, S., Zhang, Y., Lien, A., Xu, J., An Organic Molecule with Asymmetric Structure Exhibiting Aggregation-Induced Emission, Delayed Fluorescence, and Mechanoluminescence. *Angew. Chem. Int. Ed.* **54**, 874−878 (2015).

S3) Gan, S., Luo, W., He, B., Chen, L., Nie, H., Hu, R., Qin, A., Zhao, Z., Tang, B. Z. Integration of aggregation-induced emission and delayed fluorescence into electronic donor−acceptor conjugates. *J. Mater. Chem. C,* **4**, 3705−3708 (2016).

S4) Xie, Z., Chen, C., Xu, S., Li, J., Zhang, Y., Liu, S., Xu, J., Chi, Z. White-Light Emission Strategy of a Single Organic Compound with Aggregation-Induced Emission and Delayed Fluorescence Properties. *Angew. Chem. Int. Ed*. **54**, 7181−7184 (2015).

S5) Li, W., Huang, Q., Yang, Z., Zhang, X., Ma, D., Zhao, J., Xu, C., Mao, Z., Zhang, Y., Chi, Z. Activating Versatile Mechanoluminescence in Organic Host–Guest Crystals by Controlling Exciton Transfer. *Angew. Chem. Int. Ed*. **58**, 22645−22651 (2020).

S6) Qi, Y., Wang, Y., Yu, Y., Liu, Z., Zhang, Y., Qi, Y., Zhou, C. Exploring highly efficient light conversion agents for agricultural film based on aggregation induced emission effects. *J. Mater. Chem. C,* **4**, 11291−11297 (2016).

S7) Huang, B., Ban, X., Sun, K., Ma, Z., Mei, Y., Jiang, W., Lin, B., Sun, Y. Thermally activated delayed fluorescence materials based on benzophenone derivative as emitter for efficient solution-processed non-doped green OLED. *Dyes Pigments*. **133**, 380−386 (2016).

S8) Xu, S., Duan, Y., Manghnani, P., Chen, K, C., Kozlov, S. M., Liu, B. Stereoisomerization during Molecular Packing. *Adv. Mater.* **33***,* 2100986 (2021).

S9) Minami, Y., Imamura, S., Matsuyama, N., Nakajima, Y., Yoshida M. Catalytic thiolation-depolymerization-like decomposition of oxyphenylene-type super engineering plastics *via* selective carbon–oxygen main chain cleavages. *Commun. Chem*. **7**, 37 (2024).

S10) Chalmers, J. M., Gaskin, W. F. & Mackenzie, M. W. Crystallinity in Poly(AryI-Ether-Ketone) Plaques Studied by Multiple Internal Reflection Spectroscopy. *Poly. Bull.* **11**, 433−435 (1984).

S11) Minami, Y., Honobe, R., Tsuyuki, S., Sato, K. & Yoshida, M. Facile Depolymerization of thermally stable polyetherethersulfone and polyetheretherketone using hydroquinone and bases. *ChemSusChem*. e202401778 (2025).

S12) Minami, Y., Matsuyama, N., Takeichi, Y., Watanabe, R., Mathew, S. & Nakajima, Y. Depolymerization of robust polyetheretherketone to regenerate monomer units using sulfur reagents. *Commun. Chem.* **6**, 14 (2023).

S13) Discekici, E. H., Treat, N. J., Poelma, S. O., Mattson, K. M., Hudson, Z. M., Luo, Y., Hawker, C. J. & de Alaniz, J. R. A highly reducing metal-free photoredox catalyst: design and application in radical dehalogenations. *Chem. Commun.* **51**, 11705−11708 (2015).

S14) González-Muñoz, D., Martín-Somer, A., Strobl, K., Cabrera, S., De Pablo, P. J., Díaz-Tendero, S., Blanco, M. & Alemán, J. Enhancing visible-light photocatalysis via endohedral functionalization of single-walled carbon nanotubes with organic dyes. *ACS Appl. Mater. Interfaces* **13**, 24877−24886 (2021).
