# Supplementary material for: Amination–degradation of super engineering plastics for the construction of surface emissive resin materials
Source: Commun Chem. 2026 Apr 30;9:233. doi: 10.1038/s42004-026-02051-1 (PMC13338230; doi:10.1038/s42004-026-02051-1)
Supplement: Supplementary file 3 — Description of Additional Supplementary Files [file 42004_2026_2051_MOESM3_ESM.pdf]

Description of Additional Supplementary Files

File Name: Supplementary Data 1
Description: Original ^{1}H and ^{13}C NMR spectra

File Name: Supplementary Data 2
Description: Source data for charts: Fig. 4d, 4e, 5a, 5b, 6c, 6d, 8c, and 8d.
