# Supplementary material for: Amination–degradation of super engineering plastics for the construction of surface emissive resin materials
Source: Commun Chem. 2026 Apr 30;9:233. doi: 10.1038/s42004-026-02051-1 (PMC13338230; doi:10.1038/s42004-026-02051-1)

Supplementary Data 1

NMR charts of obtained chemicals.

^{1}H NMR (600 MHz) spectrum of **3a** ($CDCl_3$)

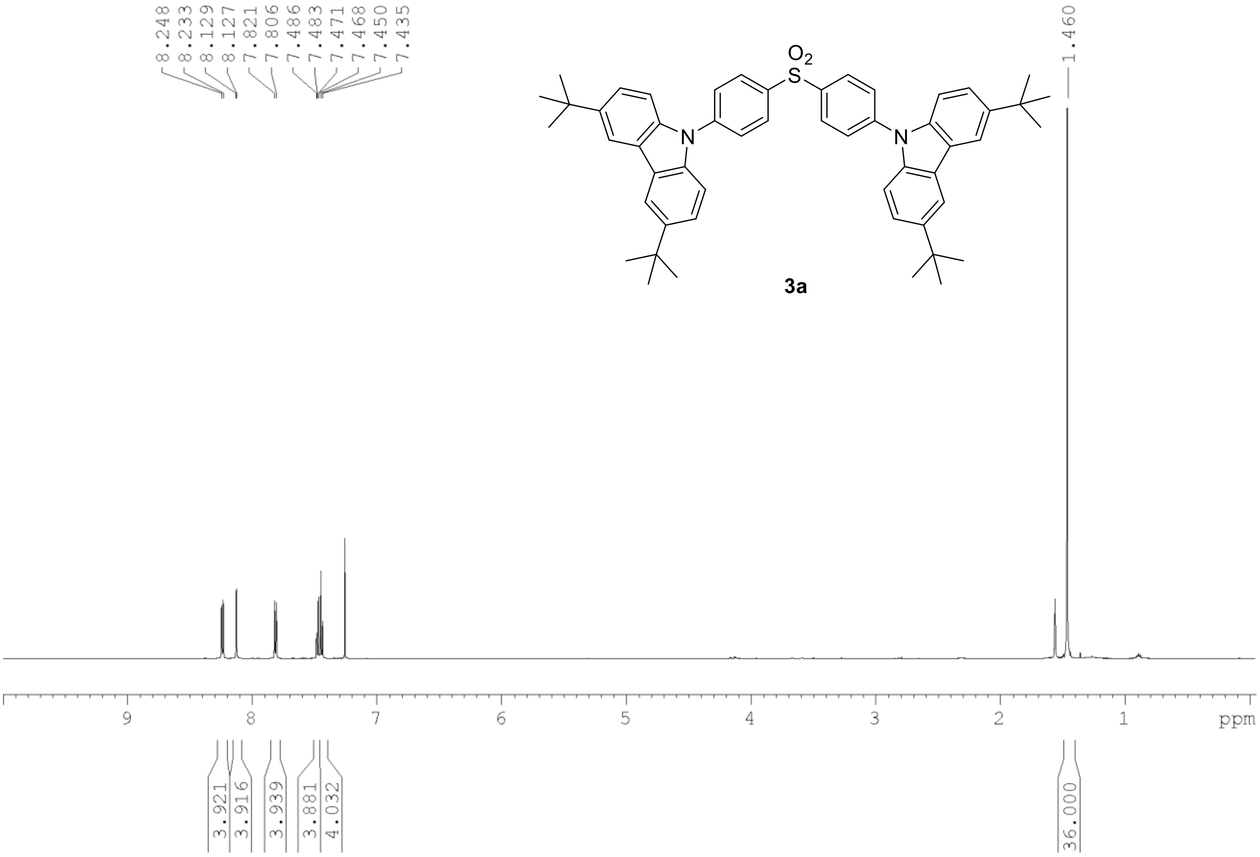

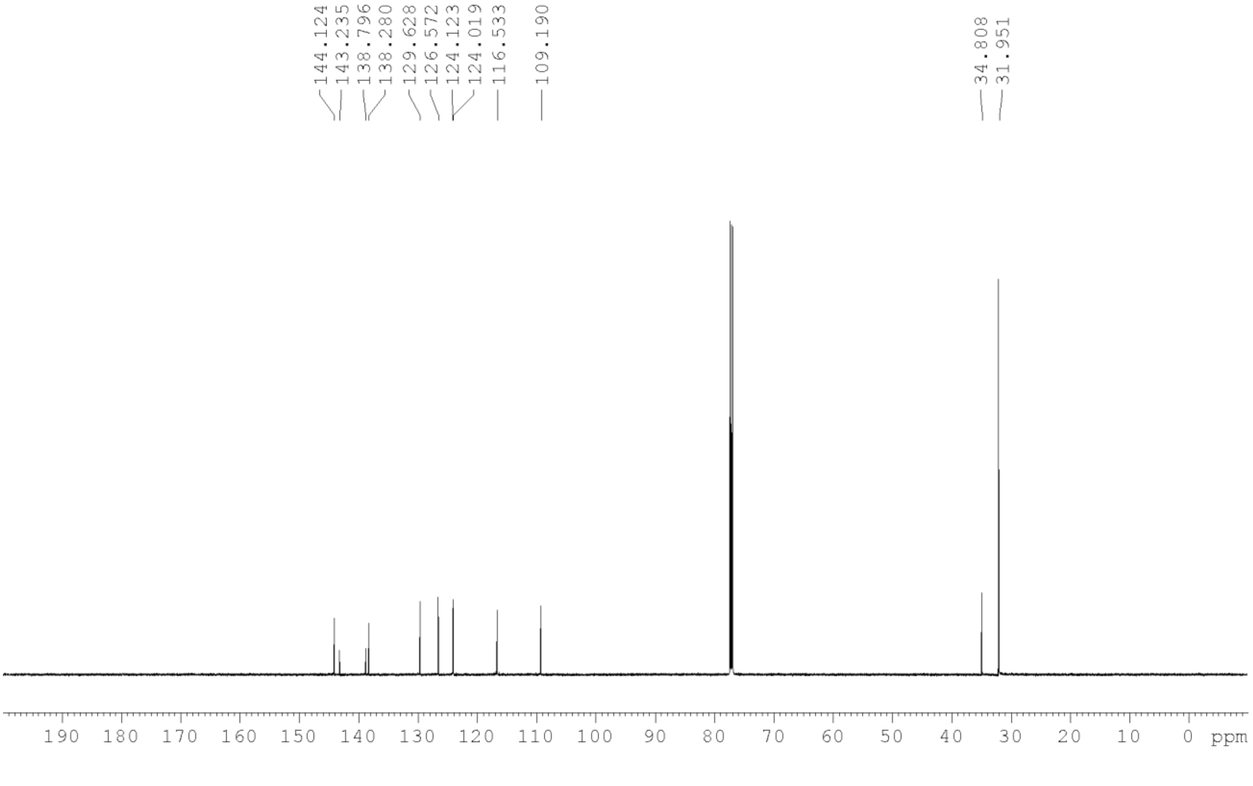

^{1}H NMR (600 MHz) spectrum of **3b** ($CDCl_3$)

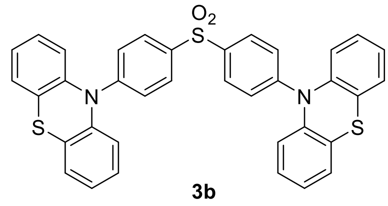

3b

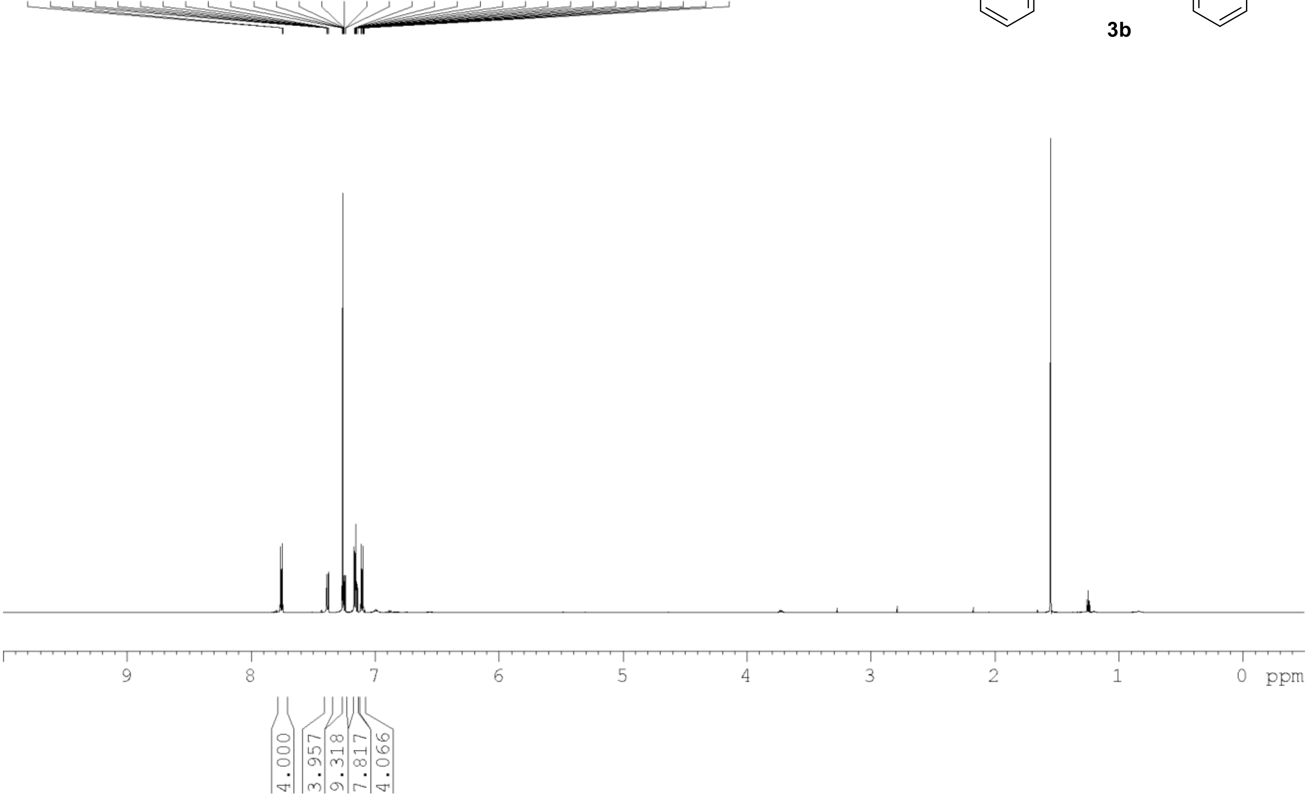

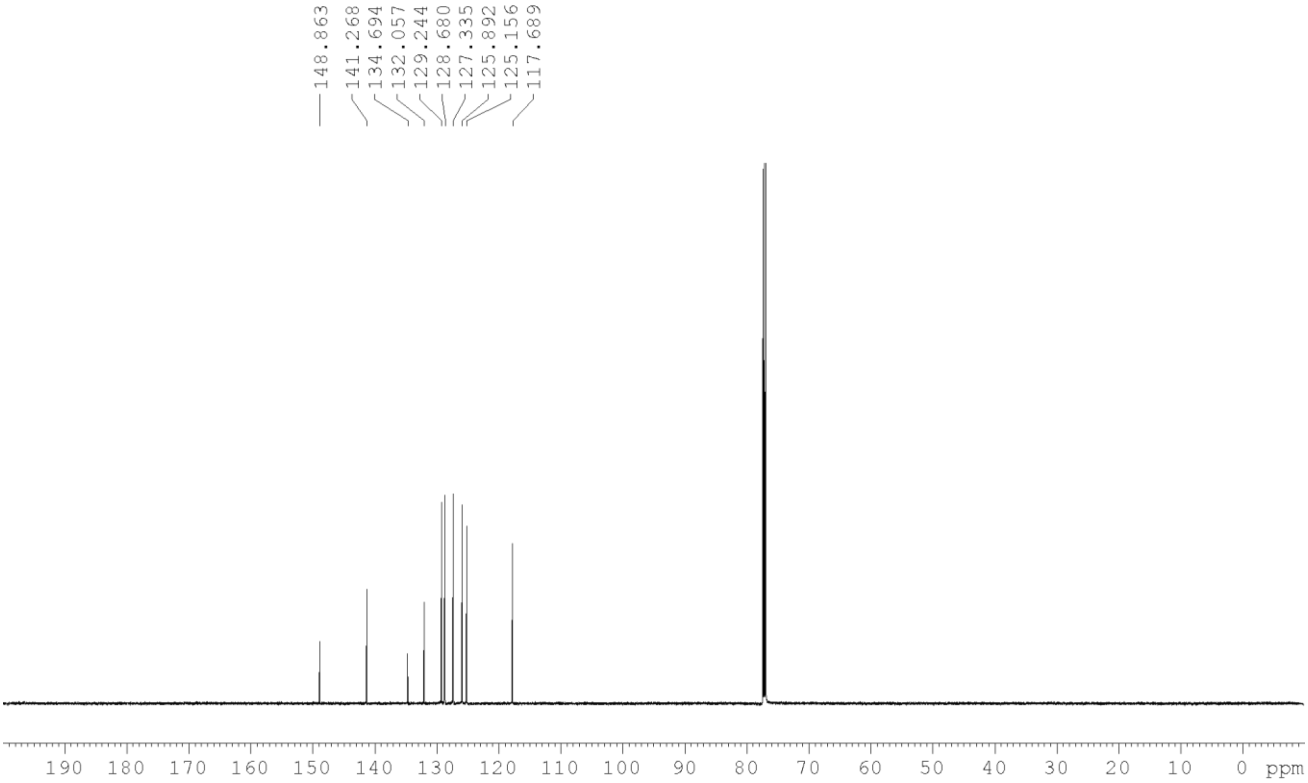

^{1}H NMR (600 MHz) spectrum of **3c** ($CDCl_3$)

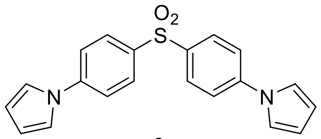

3c

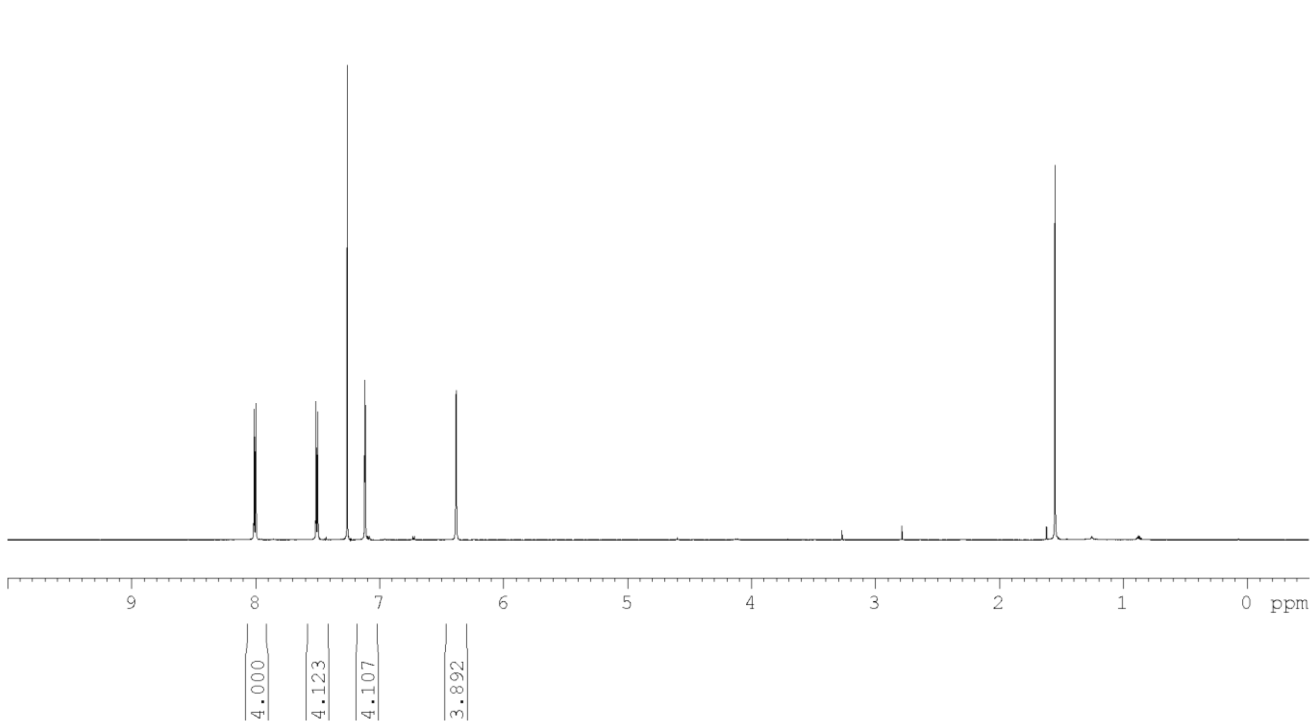

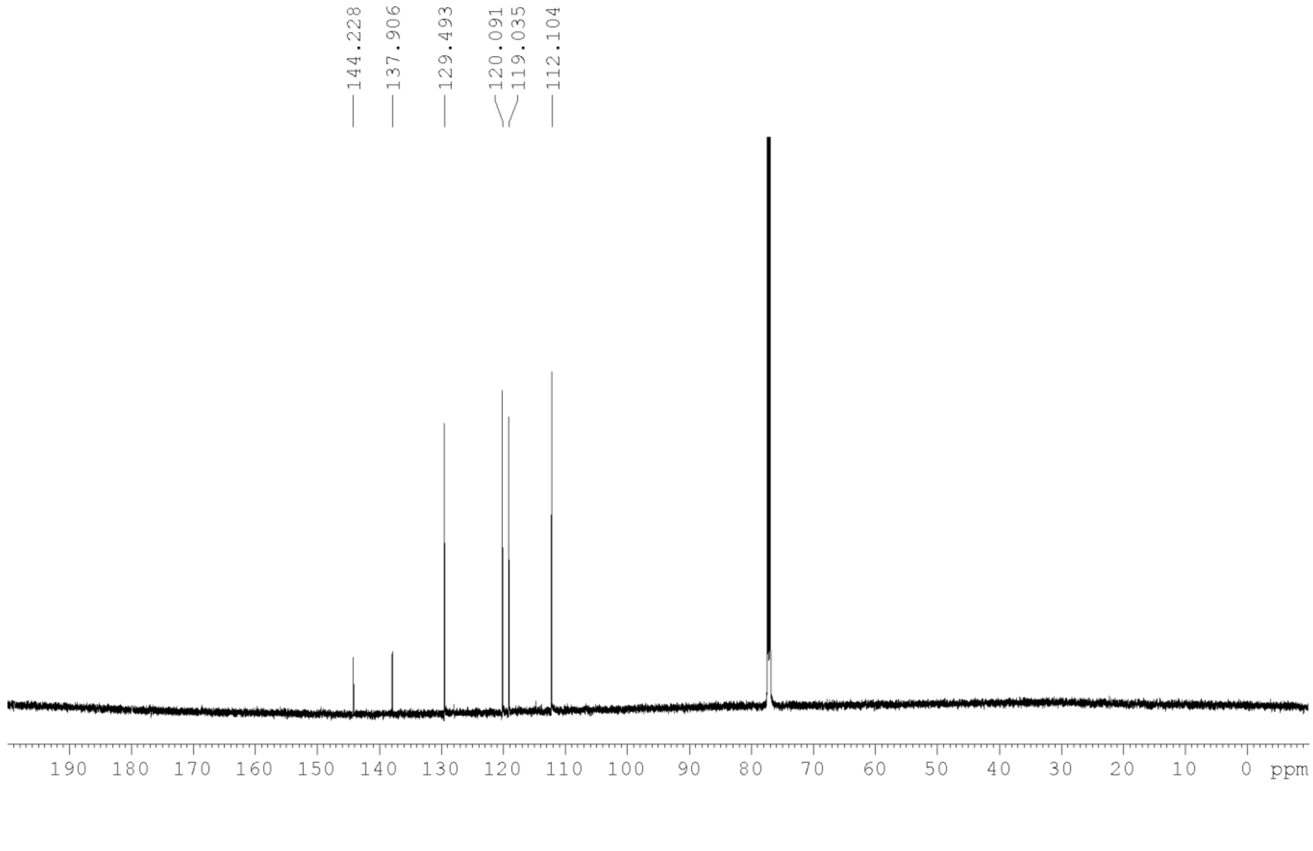

^{1}H NMR (600 MHz) spectrum of **7a** ($CDCl_3$)

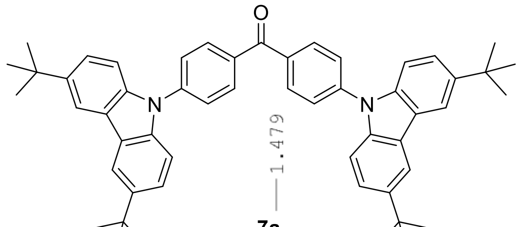

7a

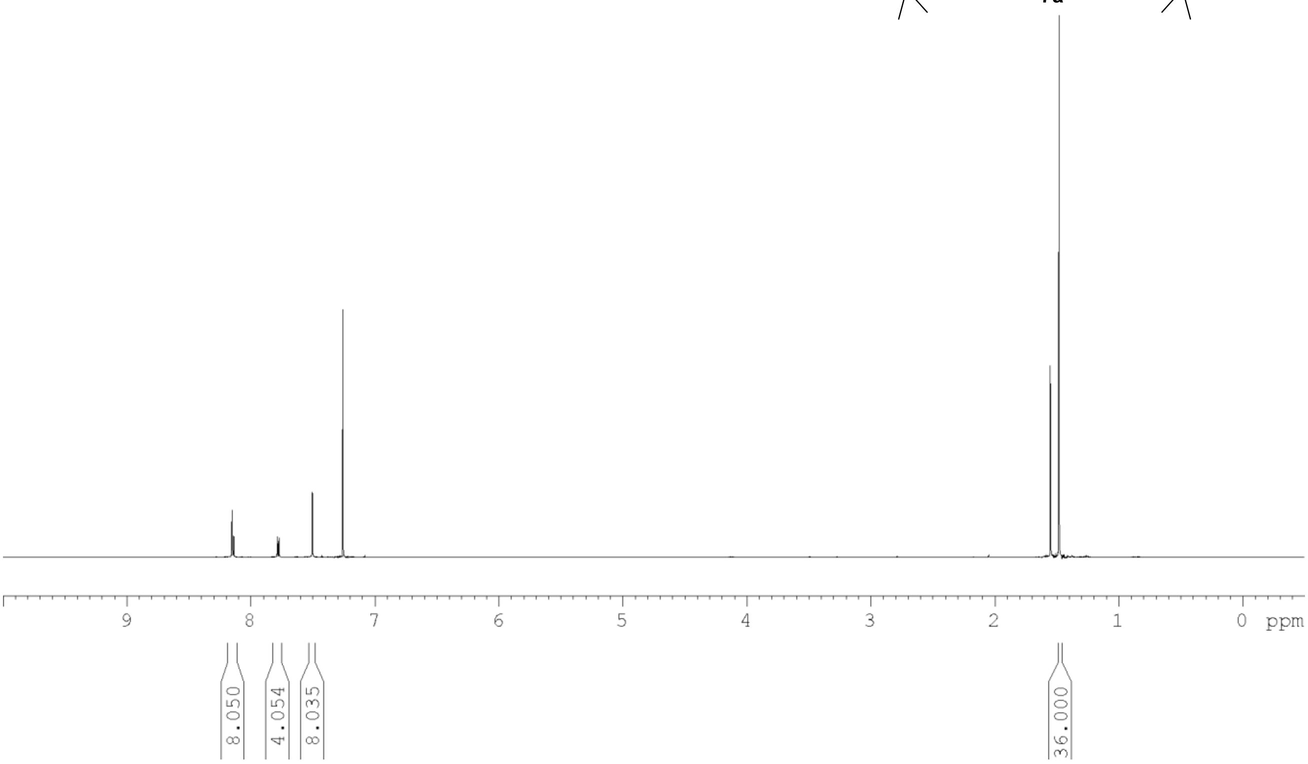

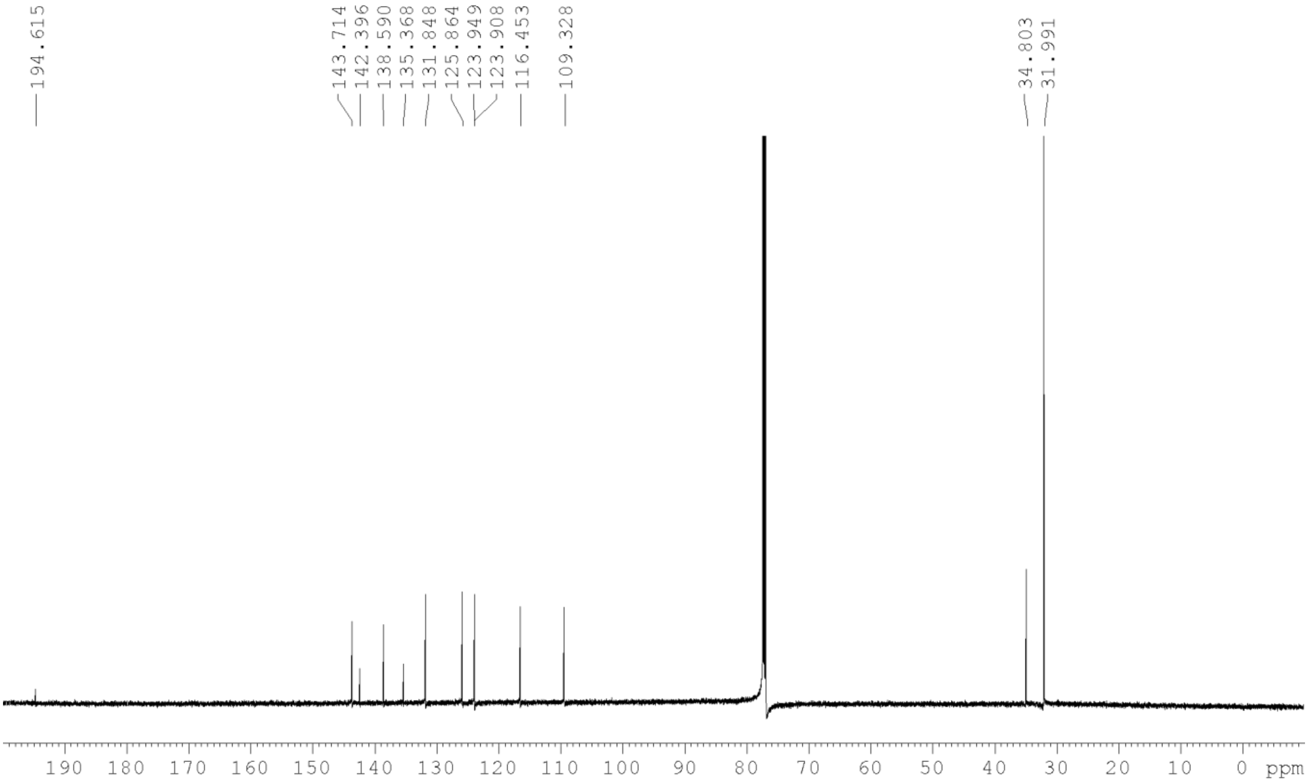

^{1}H NMR (600 MHz) spectrum of **7b** ($CDCl_3$)

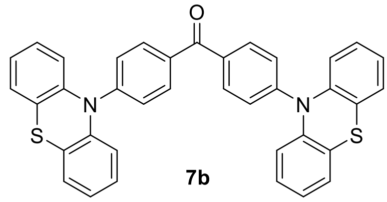

7b

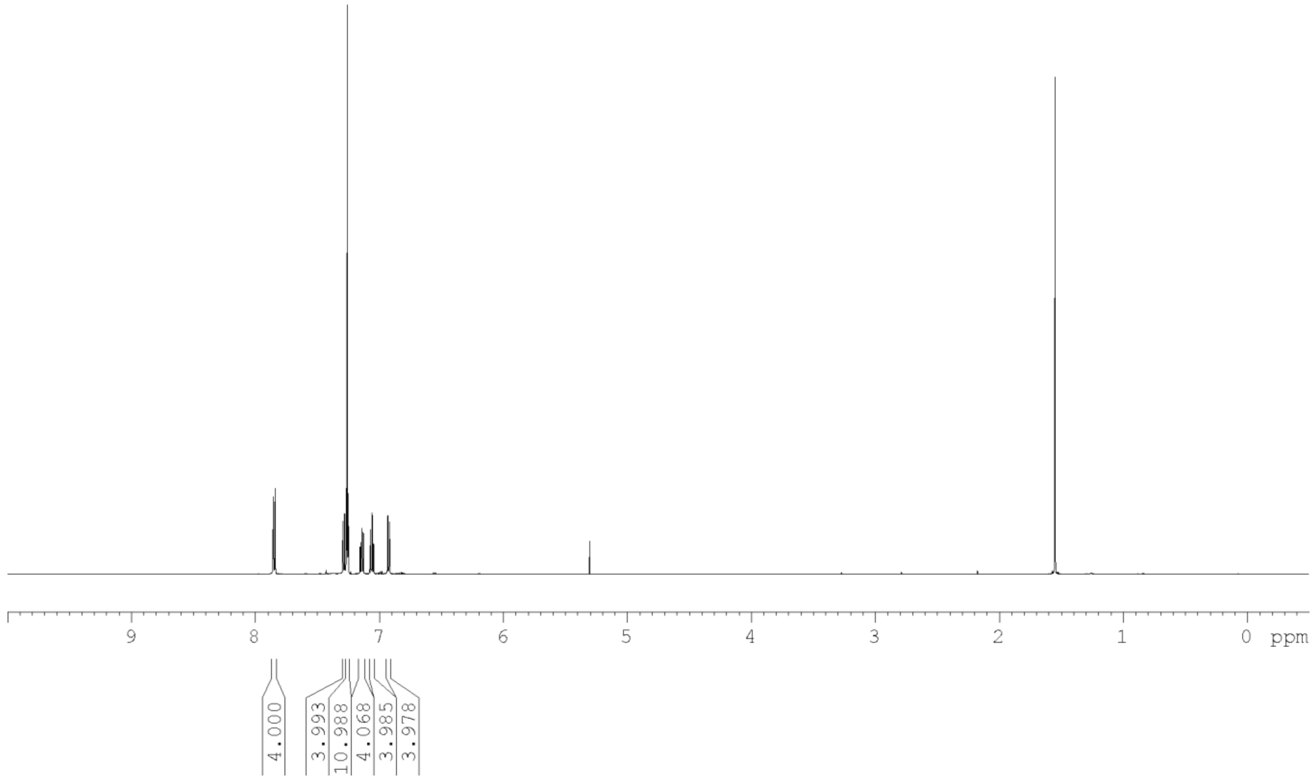

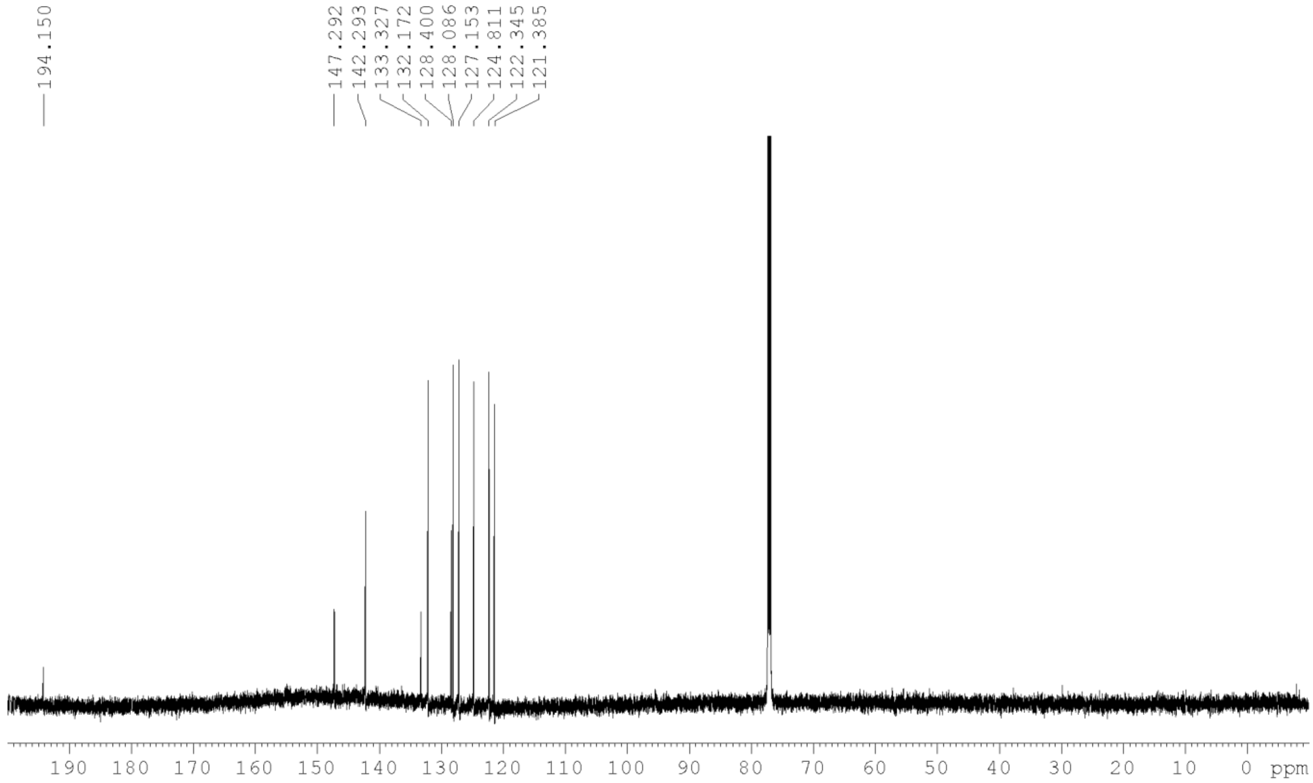

^{1}H NMR (600 MHz) spectrum of **9** ($CDCl_3$)

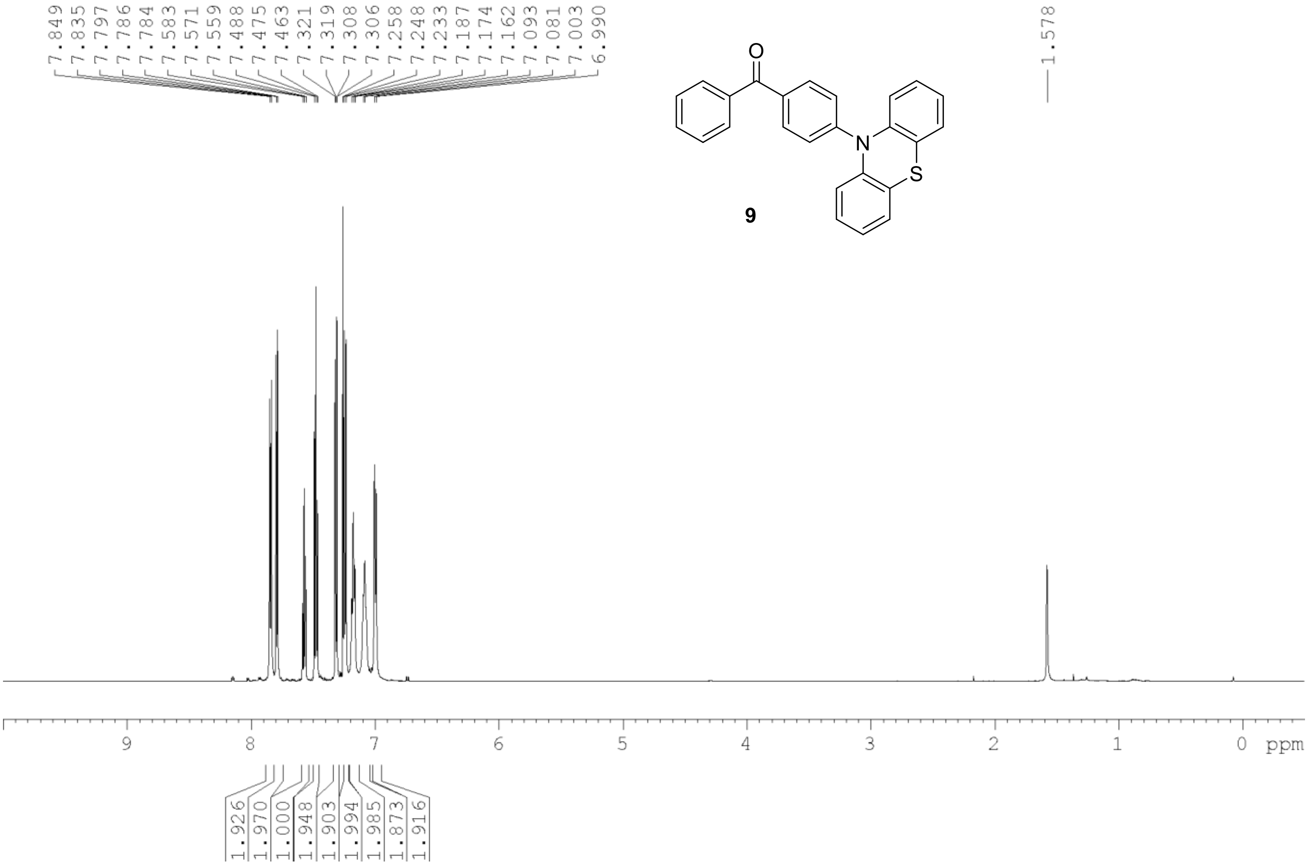

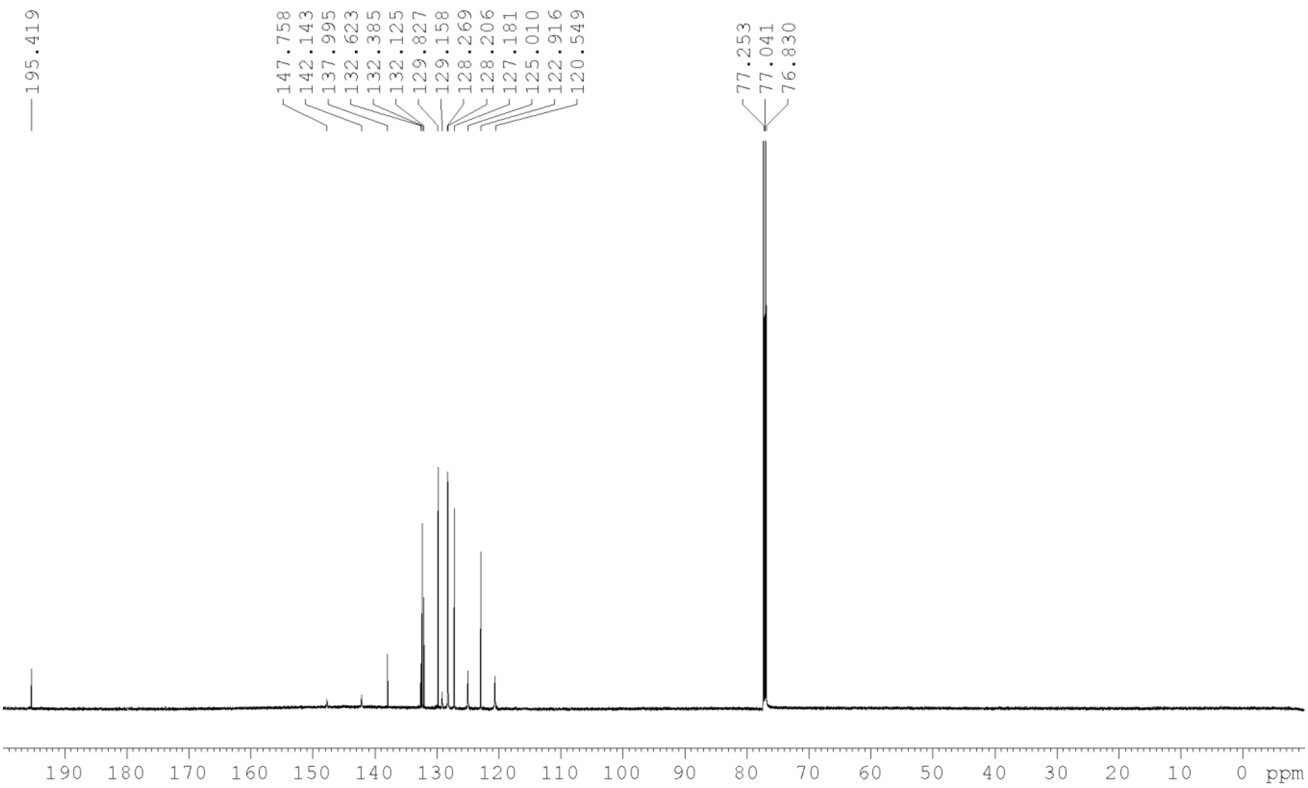

Supplement: Supplementary file 4 — Supplementary Data 1 [file 42004_2026_2051_MOESM4_ESM.pdf]
