# Supplementary material for: Amination–degradation of super engineering plastics for the construction of surface emissive resin materials
Source: Commun Chem. 2026 Apr 30;9:233. doi: 10.1038/s42004-026-02051-1 (PMC13338230; doi:10.1038/s42004-026-02051-1)
Supplement: Supplementary file 1 — Transparent Peer Review file [file 42004_2026_2051_MOESM1_ESM.pdf]

nature portfolio

Peer Review File

Amination–Degradation of Super Engineering Plastics for the Construction of Surface Emissive Resin Materials

Corresponding Author: Dr Yasunori Minami

This file contains all reviewer reports in order by version, followed by all author rebuttals in order by version.

Attachments originally included by the reviewers as part of their assessment can be found at the end of this file.

Version 0:

Reviewer comments:

Reviewer #1

(Remarks to the Author)
The detailed comments are attached as file.

The manuscript can be accepted, after additional editorial revisions.

Reviewer #2

(Remarks to the Author)
My recommendation is either major revision and/or submission to another journal. The chemistry here is not that novel for a communication and there substantial issues with the chemistry.

There are two issues. The first is whether the SNAr reaction of an amine with PEEK or PSU is really novel given the authors' prior work in references 23, 24, 25, and 27 showing that a variety of nucleophiles effect a very similar SNAr reaction. There are other references to similar chemistry for polyethersulfones (e.g. reference 31) too. Thus, I would argue that the SNAr chemistry as a method to modify PEEK and similar polymers is pretty well established and not novel enough for publication as a communication. Specifically, the use of amines or a pyrrolide anion as nucleophiles isn't sufficient for publication given the well-established knowledge base of SNAr chemistry generally and for polymers like PEEK and PES. The second and I think deciding issue is whether the products of this chemistry – the photoemissive surface functionalization and/or the photocatalyst chemistry supplement this relatively trivial extension of SNAr chemistry enough to make the paper significant. The bulk of my review addresses these second two issues.

The first of the two new aspects of the derivatization chemistry is that the functionalization converts a "pale blue fluorescent" parent PEEK into a surface-functionalized emissive PEEK derivative with a yellow fluorescence. I did not understand why that was important for either a powder or a plate. The idea of converting PSU into OLED materials is interesting. However, reference 31 describes that idea for the related polymer PES making the PSU chemistry very derivative. Second, while this specific type of surface functionalization for PEEK isn't known to my knowledge, the 2.03 weight percent of PTZ on the surface is a very high loading for surface functionalization. It suggests that the powder has a very high surface area or that the powder is a microfine powder. The authors could easily address this issue by carrying out a surface area analysis – a very normal procedure when discussing surface modification - or they could physically characterize the size of the powder.

The second application of this modified PEEK is that the products can become polymer-supported photoredox catalysts. The synthesis of polymer-supported recyclable organo and transition metal photoredox catalysts is however not new. References starting in 2015 including Swager's work in Nature Communications (doi.org/10.1038/s41467-022-29811-6), Liu's work in Journal of Catalysis (https://doi.org/10.1016/j.jcat.2022.01.024), Bergbreiter's work in Polymer Chemistry (doi.org/10.1039/C6PY00114A), Matterna's work in ChemEuJ (DOI.org/10.26434/chemrxiv.14132213.v1), Hobbs' work in Tetrahedron Letters (DOI.10.1016/j.tetlett.2020.152759), and probably others describe these sorts of polymer-supported catalysts. The paper lacks references to these examples.

References to prior work are required but aren't the major issue for the catalysis studies. I've a real problem understanding

the reported yields and their significance. First, they are rather modest. Moreover, for known chemistry, they involve very small scale reactions and no product isolation. They would be more convincing if a 1-10 mmol scale reaction were included. Second, there is an issue about the background uncatalyzed reaction. The supporting information says that the polymer-supported catalyst is ca. 10 times more effective than the soluble catalyst (they use 0.5 mol% loading versus Hawker's 5 mol%). That is a surprising positive feature that should be discussed in the paper. However, this surprising feature could represent a background reaction. Both the caption for Figure S19, the Figure s19 picture and the discussion of recyclability (Figure S20) note that the reactor was used at a distance of 0.1 cm. However, the supporting experimental also says "When the distance between the solution and the light source is reduced to 0.1 cm, the reaction proceeds without a catalyst to form 14 in 51% yield." The suggests that some or much of this chemistry has nothing to do with catalysis.

There are other minor issues. Some examples. An -SO3H group is a sulfonic acid group, not a sulfo group. The authors early on suggest that surface functionalization inhibits reuse and recycling. Most of this chemistry involves modifying the hydroquinone ring. This paper and the authors' prior work has generally not described usefully recycling or reusing the hydroquinone ring so it seems to be a 'straw man' argument. The first paragraph of p. 4 should be rewritten to make it clearer.

Version 1:

Reviewer comments:

Reviewer #1

(Remarks to the Author)
Minor corrections:
- Introduction first line: "R" change to: "10R"
- Introduction: "such as sulfonation, in which a sulfo (SO3H) group is introduced" change to: "sulfonic acid group (SO3H)"
- Introduction: "Friedel–Crafts acylation to introduce carbonaceous groups" change to "acyl groups"
- Introduction page 2: "Herein, we report a degradation method for super engineering plastics such as PSU and PEEK using azacyclic compounds to form donor–acceptor–donor-type molecules, which allowed functionalizing the resin surface to form luminescent PEEK powder and plates." comment to this: This mixes two things together. D-A-D molecules you receive by degradation. The luminescent powder or plate you receive by surface functionalization. These molecules ar not D-A-D but just D-A, right, bond to the rest of the polymer chain. So separate both things here to avoid misunderstanding.

Reviewer #2

(Remarks to the Author)
I had several issues with the original manuscript. While the authors have made some arguments addressing my comments, several issues remain and I do not believe this work merits publication as a Communication. I suggest another RSC journal focused on Polymer Chemistry would be a better venue for this paper which I believe lacks novelty and is an incremental advance.

I do not agree with the authors that the surface modification versus bulk modification is especially novel or that the chemistry is very surface selective. My second point was that the products as catalysts too are also not novel nor likely to be very useful. The experiment using a relatively small but larger 1-mmol scale reaction that leads to 67% yield of products is a useful addition to the manuscript. However, that experiment suggests that these catalysts are not superior to existing catalysts. The addition of references to other catalytic work is important as is the recycling study. However, the catalysts are not in my opinion competitive with existing catalysts so these catalytic studies are not sufficient to justify publication as a communication. They usefully expand the scope of the study though for a a full paper. Finally the authors studied particle size studies. However, the surface areas o articles with these sizes are too small to account for the 'surface' loadings reported. The loadings the aurthors described are more consistent with modification that includes significant bulk polymer modification.

In summary, the paper is improved but neither the polymer degradation nor the catalyst chemistry has the novelty consistent with typical standards for Chemical Communications.

Version 2:

Reviewer comments:

Reviewer #1

(Remarks to the Author)
Thank you for the improvements.

Open Access This Peer Review File is licensed under a Creative Commons Attribution 4.0 International License, which permits use, sharing, adaptation, distribution and reproduction in any medium or format, as long as you give appropriate credit to the original author(s) and the source, provide a link to the Creative Commons license, and indicate if changes were made.
In cases where reviewers are anonymous, credit should be given to 'Anonymous Referee' and the source.
The images or other third party material in this Peer Review File are included in the article's Creative Commons license, unless indicated otherwise in a credit line to the material. If material is not included in the article's Creative Commons license and your intended use is not permitted by statutory regulation or exceeds the permitted use, you will need to obtain permission directly from the copyright holder.
To view a copy of this license, visit https://creativecommons.org/licenses/by/4.0/

Point-by-Point response to referees

All the responses made are summarized in followings.
Revised positions were highlighted by yellow.

Comments from Reviewer 1:

1-1) My main question is, and I would like to ask the author to discuss this in the manuscript, are the shown reactions "recycling" of the used polymers? If not, don't use upcycling and maybe don't argue with recycling or a circular technology.

Compare your phrase:

"From the viewpoint of circular technology, facile functionalization methods that allow chemical recycling while preserving the main-chain structure are highly desirable."

Recycling of the Polymers fits to your latest research, cleaving the polymer chains and produce monomers, but not to the functionalization of the polymers. As you wrote: "In fact, this surface functionalization [from the literature] is important for the development of dental materials. However, the introduction of functional groups into the main-chain hinders the chemical recycling." And this is what you do with your fluorophores, too.

Thereby I suggest, rewrite the introduction, don't focus on recycling or circularity, call it repurpose of the polymers, which is a 10R principle.

Response to 1-1)

We appreciate Reviewer 1 for the valuable comments and the suggestion. We agree with this valuable suggestion. So, we revised the abstract and the introduction based on "Repurpose" instead of recycling and a circular technology. This perspective also aligns with the principle of efficient use of carbon resources.

For example, the first sentence of the abstract was changed to "The advent of effective use of carbon resources has demanded for the future society. This importance is evident from the recent evolution of the "R" framework such as "10R" including "Repurpose".". And the first sentence of the introduction was changed to "To realize an effective use of carbon resources, the implementation of the recent evolved "R" framework including "Repurpose": recreation into something new, based on 3R ("reduce, reuse, and recycle") of existing carbonaceous materials such as plastics is essential.".

The last part of the second paragraph was revised according to Reviewer 1's comment as follow: "These elegant methodologies are considered effective in contributing to "Repurpose". Notably, these methods are based on the functionalization of the main chain. From the viewpoint, the development of facile functionalization methods while preserving the main-chain structure are expected to attribute to the advancement of "Repurpose".". During this revising, relating sentences were also revised in the abstract and the 1st paragraph. These revisions were shown by yellow highlighted.

In addition, to support this revising, we newly examined the depolymerization of prepared PEEK-PTZ powder with hydroquinone based on our previous reports to form the monomers. This result was added to the Fig. 4f in the manuscript and Fig. S16 in the supplementary information. The explanation about this depolymerization was added in Page 8, Also, the degradation of PEEK-PTZ powder with an alkanethiol was examined to form dithiofunctionalized benzophenone and hydroquinone. This result was added in the

supplementary information, Fig. S17, line 22.

1-2) In the second sentence of the abstract as well as at the beginning of the introduction the 3R framework is mentioned. The 3Rs have evolved into 9R or 10R in the last years, which include "Repurpose" as a principle. "Upcycling" as the author calls it, is a part of the repurpose principle. So maybe the author may rewrite the introduction an describe upcycling in the context of 9R or 10R.

Response to 1-2)

We appreciate Reviewer 1 for the valuable comment. According to this comment, we revised the abstract, the introduction, and the conclusion as explained in the response to 2-1 as above.

1-3) Page 4 the first described reaction of PSU with **2a** is missing in Fig. 2, which confuses me for a while, because I looked for product **3a**, which is produced in Fig. 2 under b) from PEES. Add PSU + 2a to Fig. 2 under a) as third arrow, to avoid confusion.

Response to 1-3)

We appreciate Reviewer 1 for the valuable comment. 3,6-Di-tert-butylcarbazole **2a** and the corresponding product **3a** were first shown in Table 1. Since Table 1 is normally placed before Fig. 2, we have followed this sequence in our notation. But, in the format, Tables were placed at the end of the manuscript, which we think caused confusion. To avoid this situation, we placed Table 1 and Table 2 in their original order. We think this revision has made it easier for you to understand.

1-4) You nicely name the nucleophiles **2a**, **2b** and **2c** as well as the products **3a**, **3b** and **3c**. So it is clear which nucleophile produces which product. For product **7** instead, you produce with nucleophile **2c** the product **7b**. You may think about call it **7c** for consistency. Additionally, why you don't use 2b with PEEK?

Response to 1-4)

We appreciate Reviewer 1 for the valuable comment. To avoid the confusion about the numbering, the order of pyrrole and phenothiazine was switched. The revised compound numbers are phenothiazine for **2b** and pyrrole for **2c**.

About pyrrole, we did not examine the reaction with PEEK. This is because we confirmed the precedence of the reaction of pyrrole with PSU, which is the similar molecular structure of PEEK. Also, we think that pyrrole is the similar reactivity to **2a** under the present degradation conditions. Thus, we did not examine the reaction of PEEK with pyrrole. Another reason is that carbazole **2a** and phenothiazine were necessary for the degradative surface functionalization in the latter half.

1-5) In Fig. 2 the letters a, b and c are below the entry instead of above.

Response to 1-5)

We appreciate Reviewer 1 for the valuable comment. Based on the published version, we replaced a, b, and c to a), b), and c) to avoid your mentioned confusion.

1-6) Fig. 2, entry b and c, also Hydrochinon will be produced as BPA from PSU. Add it to have a correct/complete reaction. Have you found it in NMR and can evaluate the amount as for BPA?

Response to 1-6)

We appreciate Reviewer 1 for the valuable comment. Actually, we detected the generation of hydroquinone by ^{1}H NMR spectroscopy from the degradation of PEES and PEEK. However, due to the water treatment after the degradation, the analyses of exact amounts of hydroquinone were not determined. Based on this observation, we newly added the sentence explaining the detection of hydroquinone after the degradation in Page 6, line 4-5. Also, we revised the experimental procedures and their figures of the degradation of PEES and PEEK in the supplementary information. In this relation, we newly examined the degradation of PEEK-PTZ powder with an alkane thiol mediated by NaOH, which afforded dialkylthio-functionalized benzophenone and hydroquinone in both about 80% yields, respectively. This result was newly added in the supplementary information, Fig. S17.

1-7) Page 7, the abbreviation **6h** is not well chosen, because it is similar to the duration of the reaction as in **101h**. Try to find something better, maybe **6DMI** as you used later for **10DMAc**.

Response to 1-7)

We appreciate Reviewer 1 for the valuable comment. We changed $\mathbf{6^h}$ to **6-heat** and $\mathbf{15^h}$ to **15-heat** to make the numbering easier to understand.

1-8) Page 10: "In addition, the PEEK-PTZ powder showed recyclability (Supplementary Fig. S20)." It is an important finding to show the reactivity of the catalyst for several cycles (maybe reuse instead of recycle?). I suggest to add this to the manuscript instead of the SI.

Response to 1-8)

We appreciate Reviewer 1 for the valuable comment. We added new Fig. 7 showing that PEEK-PTZ powder could be reused as a photoredox catalyst. In this relation, the figure to show this reusability was deleted in the supplementary information.

1-9) Table 1: The footnotes b-e should be placed into the table. I suggest to add the amount of solvent and base into the respective columns. This increases clarity and allows the reader to recognise changes in the reaction conditions more quickly. Be consistent using equivalent (as in the

reaction) or the given mmol as in the subline of the table.

Response to 1-9)

We appreciate Reviewer 1 for the valuable comment. We revised the notion of Table 1 to follow Reviewer 1's comment.

1-10) Table 2: You give average yields of 14 for the different catalysts. Can you give a standard deviation? Or additional to average 90% also (between 70% and 98%). This would give additional information to the reader, towards the reproducibility of the catalytic reaction.

Response to 1-10)

We appreciate Reviewer 1 for the valuable comment. We added the whole independent yields (87%, 89%, 91%, and 92%) in the footnote. Based on the comments from Reviewer 1, we only showed the result using 4-chlorobenzonitrile in the manuscript. But, all the result using 4-bromobenzonitorile were shown in the supplementary information.

Comments from Reviewer 2:

2-1) The first is whether the SNAr reaction of an amine with PEEK or PSU is really novel given the authors' prior work in references 23, 24, 25, and 27 showing that a variety of nucleophiles effect a very similar SNAr reaction. There are other references to similar chemistry for polyethersulfones (e.g. reference 31) too. Thus, I would argue that the SNAr chemistry as a method to modify PEEK and similar polymers is pretty well established and not novel enough for publication as a communication. Specifically, the use of amines or a pyrrolide anion as nucleophiles isn't sufficient for publication given the well-established knowledge base of SNAr chemistry generally and for polymers like PEEK and PES.

The second and I think deciding issue is whether the products of this chemistry – the photoemissive surface functionalization and/or the photocatalyst chemistry supplement this relatively trivial extension of SNAr chemistry enough to make the paper significant. The bulk of my review addresses these second two issues.

Response to 2-1)

We appreciate Reviewer 2 for the valuable comment. Exactly, we have already demonstrated that polyetheretherketone (PEEK) and polysulfones (PSU) such as PSU and PEES underwent the degradation by via main-chain selective cleavage. As shown in the manuscript, Dufaud, Raynaud, and Monteil's work (reference 31) also encouraged us. The figure for reference 31 was newly added in Fig. 2c. As Reviewer 2 mentioned, the former side of the manuscript focused on the degradation of PEEK, PSU, and PEES with amine nucleophiles. As a result, we successfully observed the formation of double substituted products without generating byproducts derived from damage of main chain structures. And we could show the appropriate conditions for the formation of low weight molecules and under which surface-only degradation is performed. This surface functionalization did not almost affect the resin's heat resistance and the surface shape (described later response 2) although a decrease in molecular weight is a concern. Especially, we

could verify the surface shape based on Reviewer 2's comments. We are very grateful to Reviewer 2.

The purpose of using phenothiazine as the reactant is to demonstrate that degradative surface functionalization was successfully achieved. In fact, since yellow luminescence is only possible through the arylation on nitrogen in the phenothiazine via the main chain cleavage, the successful generation of yellow emissive PEEK-PTZ powder and plates demonstrated that the degradative surface functionalization proceeded. Based on these observations, we realized that the surface functionalization with carbazole **2a** proceeded similarly. We believe the significance of this study lies in these observations. For the explanation for this idea, we revised the first sentence of the 1st paragraph of Optimization of the degradation conditions.

2-2) The first of the two new aspects of the derivatization chemistry is that the functionalization converts a "pale blue fluorescent" parent PEEK into a surface-functionalized emissive PEEK derivative with a yellow fluorescence. I did not understand why that was important for either a powder or a plate. The idea of converting PSU into OLED materials is interesting. However, reference 31 describes that idea for the related polymer PES making the PSU chemistry very derivative.
Second, while this specific type of surface functionalization for PEEK isn't known to my knowledge, the 2.03 weight percent of PTZ on the surface is a very high loading for surface functionalization. It suggests that the powder has a very high surface area or that the powder is a microfine powder. The authors could easily address this issue by carrying out a surface area analysis – a very normal procedure when discussing surface modification - or they could physically characterize the size of the powder.

Response to 2-2)

We appreciate Reviewer 1 for the valuable comment. As mentioned above response to 2-1), while the employed reaction was the nucleophilic amination via SNAr, we consider it significant that this degradation was applicable to the construction of novel functionalized resin materials. The importance of this methodology does not involve the functionalization of the main chain. To evaluate this characteristic, we further examined the depolymerization of PEEK-PTZ powder with hydroquinone, which provided the corresponding monomer in a similar yield compared with the reported yield from PEEK. This result was newly added on Fig. 4f. In addition, the NaOH-mediated degradation of PEEK-PTZ powder with an alkane thiol furnished the formation of dialkylthio-benzophenone and hydroquinone in high yields, respectively. These observations were newly added in the supplementary information, Fig. S16 and S17.

We thought that the suggestion about surface functionalization is important. Thus, we measured the size distribution of used PEEK powder and prepared PEEK-PTZ powder. The powder-size distribution of used PEEK (mean particle size: 50 μm written in the catalog) was assessed, revealing powder with diameters of approximately 50 mm as the fine powder. After the surface degradative functionalization, the powder size tends to decrease slightly to 40 nm (new Supplementary Fig. S14). At least, no reaction-induced aggregation occurred. In addition, original PEEK powder **6** and prepared PEEK-PTZ powder **10'** were analyzed by scanning electron microscopy (SEM), whose surface shapes showed no visible differences between **6** and **10'** (new Supplementary Fig. S15). These results mean that the size decreased by the surface

degradation while maintaining the resin's shape.

In this relation, we revised the *Acknowledgements.*

2-3) The second application of this modified PEEK is that the products can become polymer-supported photoredox catalysts. The synthesis of polymer-supported recyclable organo and transition metal photoredox catalysts is however not new. References starting in 2015 including Swager's work in Nature Communications (doi.org/10.1038/s41467-022-29811-6), Liu's work in Journal of Catalysis (https://doi.org/10.1016/j.jcat.2022.01.024), Bergbreiter's work in Polymer Chemistry (doi.org/10.1039/C6PY00114A), Matterna's work in ChemEuJ (DOI.org/10.26434/chemrxiv.14132213.v1), Hobbs' work in Tetrahedron Letters (DOI.10.1016/j.tetlett.2020.152759), and probably others describe these sorts of polymer-supported catalysts. The paper lacks references to these examples.

Response to 2-3)

We appreciate Reviewer 2 for the valuable comment. We added Reviewer 2's mentioned references and further *Chem. Eur. J.* 2022, *28*, e202201322 to the manuscript.

2-4) References to prior work are required but aren't the major issue for the catalysis studies. I've a real problem understanding the reported yields and their significance. First, they are rather modest. Moreover, for known chemistry, they involve very small scale reactions and no product isolation. They would be more convincing if a 1-10 mmol scale reaction were included.

Second, there is an issue about the background uncatalyzed reaction. The supporting information says that the polymer-supported catalyst is ca. 10 times more effective than the soluble catalyst (they use 0.5 mol% loading versus Hawker's 5 mol%). That is a surprising positive feature that should be discussed in the paper. However, this surprising feature could represent a background reaction. Both the caption for Figure S19, the Figure s19 picture and the discussion of recyclability (Figure S20) note that the reactor was used at a distance of 0.1 cm. However, the supporting experimental also says "When the distance between the solution and the light source is reduced to 0.1 cm, the reaction proceeds without a catalyst to form 14 in 51% yield." The suggests that some or much of this chemistry has nothing to do with catalysis.

Response to 2-4)

We appreciate Reviewer 1 for the valuable comment. Based on this comment, the 1 mmol scale of dehalogenation of 4-chlorobenzonitrile was examined. As a result, benzonitrile was formed but the yield decreased from 90% to 67% probably due to an increase in the volume of the solution. For this result, we did not examine the dehalogenation on a larger scale. Exactly, we used 0.5 mol% of Aryl-PTZ unit for this dehalogenation. This is nothing special; in the original dehalogenation reaction, a 0.5 mol% phenyl-PTZ catalyst was used to form the product in the same yield. Based on above new experiments, we revised Table 2 and the explanation paragraph in the manuscript. The new Table 2 only shows the results using 4-chlorobenzonitrile. The results of the reaction of 4-bromobenzonitrile were listed in the supplementary information.

Exactly, we confirmed that this reaction occurred without a photoredox catalyst under photo irradiation. Our purpose of this catalytic examination was to verify whether the functional

groups introduced onto the PEEK surface can be used not only for luminescence but also as catalysts in chemical reactions. In this regard, we realized that our purpose was achieved, and as Reviewer 2 mentioned, it is not the major issue in the manuscript. In the future, we intend to introduce other catalysts and thoroughly investigate the significance of this surface functionalization method of PEEK and other polymer materials.

2-5) There are other minor issues. Some examples. An -SO3H group is a sulfonic acid group, not a sulfo group. The authors early on suggest that surface functionalization inhibits reuse and recycling. Most of this chemistry involves modifying the hydroquinone ring. This paper and the authors' prior work has generally not described usefully recycling or reusing the hydroquinone ring so it seems to be a 'straw man' argument. The first paragraph of p. 4 should be rewritten to make it clearer.

Response to 2-5)

We appreciate Reviewer 2 for the valuable comment. The mentioned sentences in the introduction were revised.

We think that the recovery of hydroquinone is also important for the degradation of PEEK and PEES. In fact, the degradation of PEEK and PEES using thiols, alkali alkoxides, and alcohols provided hydroquinone with the corresponding benzophenone or diphenylsulfone derivatives. Based on this finding, we developed the depolymerization of PEEK and PEES with hydroquinone to form bisphenol type monomers containing original hydroquinone unit in used polymers. The explanation and scheme of this depolymerization of PEEK with hydroquinone was newly added to Fig. 2b. Furthermore, we newly examined the depolymerization of PEEK-PTZ powder with hydroquinone to form bisphenol type monomer in new Fig. 4f. From the viewpoints, we thought that the non-damage depolymerization method toward the hydroquinone moiety attributes to the development of the recycling method of these plastics.

In addition, this methodology is also important for the degradative functionalization of other super engineering plastics such as PSU and PEES.

Point-by-Point response to referees

All the responses made are summarized in followings.
Revised positions were highlighted by yellow.

Comments from Reviewer 1:

1) Minor corrections:

- Introduction first line: “R” change to: “10R”
- Introduction: “such as sulfonation, in which a sulfo (SO3H) group is introduced” change to: "sulfonic acid group (SO3H)"
- Introduction: “Friedel–Crafts acylation to introduce carbonaceous groups” change to "acyl groups"
- Introduction page 2: “Herein, we report a degradation method for super engineering plastics such as PSU and PEEK using azacyclic compounds to form donor–acceptor–donor-type molecules, which allowed functionalizing the resin surface to form luminescent PEEK powder and plates.” comment to this: This mixes two things together. D-A-D molecules you receive by degradation. The luminescent powder or plate you receive by surface functionalization. These molecules are not D-A-D but just D-A, right, bond to the rest of the polymer chain. So separate both things here to avoid misunderstanding.

Response to 1)

We appreciate Reviewer 1 for the valuable comments. Based on Reviewer 1’s comments, we revised the introduction.

“R” was change to “10R” in the Introduction first line.

The sentence “such as sulfonation, in which a sulfo (SO3H) group is introduced” was changed to “sulfonic acid group (SO_3H)”.

The sentence about the content of this study was revised to “Herein, we report transformation methods of PEEK aimed at the surface functionalization based on the cleavage of main chains. First, we verified the degradation of PSU, PEES, and PEEK using azacyclic compounds and bases to provide donor–acceptor–donor-type molecules. This examination aimed to understand the conditions necessary for proceeding moderately and precisely. Then, we applied this degradation to functionalize the PEEK resin surface gently to form luminescent PEEK powder and plates having D-A moiety bond to the rest of the polymer chain (Fig. 1d). The prepared luminescent PEEK materials maintained high thermal stability and were applied not only to fine powders but also to pellets and sheet materials.” This revised sentences are composed of two explanations for the degradation to form D-A-D type molecules and surface functionalization to form luminescent PEEK having D-A moiety. Especially, the purpose of the former degradation was clarified. In this relation, Fig. 1d was revised, which focuses on the surface functionalization and includes pictures of functionalized powder and the plate.

Moreover, the sentence “as the D-A unit bond to the rest of the PEEK chain on the surface” was added between “*N*-benzophenone-PTZ (BP-PTZ) moiety” and “by the main-chain cleavage using **2b**” in line 7, page 8.

Comments from Reviewer 2:

I had several issues with the original manuscript. While the authors have made some arguments addressing my comments, several issues remain and I do not believe this work merits publication as a Communication. I suggest another RSC journal focused on Polymer Chemistry would be a better venue for this paper which I believe lacks novelty and is an incremental advance.
I do not agree with the authors that the surface modification versus bulk modification is especially novel or that the chemistry is very surface selective.
My second point was that the products as catalysts too are also not novel nor likely to be very useful. The experiment using a relatively small but larger 1-mmol scale reaction that leads to 67% yield of products is a useful addition to the manuscript. However, that experiment suggests that these catalysts are not superior to existing catalysts. The addition of references to other catalytic work is important as is the recycling study. However, the catalysts are not in my opinion competitive with existing catalysts so these catalytic studies are not sufficient to justify publication as a communication. They usefully expand the scope of the study though for a full paper.
Finally the authors studied particle size studies. However, the surface areas of particles with these sizes are too small to account for the 'surface' loadings reported. The loadings the aurthors described are more consistent with modification that includes significant bulk polymer modification.

Response to 2)

We appreciate Reviewer 2 for the valuable comment. Based on Reviewer 2's comments, we revised the manuscript.

- About this comment "I do not agree with the authors that the surface modification versus bulk modification is especially novel or that the chemistry is very surface selective." -

As mentioned by Reviewer 2, several functionalization methods on the PEEK surface have been known to date written in the introduction section, which proceeded through the introduction or substitution on main chain. The present method occurred the surface functionalization by the cleavage of main chain without the modification of the main chain structure. Based on the present characteristics, we expect that the present finding can be used to complement conventional developed methodologies, and it is applicable to other super engineering plastics.

The first submission version and the previous revised version showed that the scrapped surface of white-emissive PEEK plate **17** did not emit unlike the original white emissive functionalized surface under 365 nm light irradiation. In addition, we newly added the observations that the cut surfaces of functionalized PEEK pellets, purchased PEEK plate, and **15** did not emit. These results were newly added to Figure Fig. 8b, supplementary Fig. S25c, S31, and S34b. These observations supported the degradative functionalization proceeded on the PEEK surface. In this relation, corresponding explanations were revised in the manuscript, last lines 6-8 in page 14.

- My second point was that the products as catalysts too are also not novel nor likely to be very useful. The experiment using a relatively small but larger 1-mmol scale reaction that leads to 67% yield of products is a useful addition to the manuscript. However, that experiment suggests that these catalysts are not superior to existing catalysts. The addition of references to other catalytic work is important as is the recycling study. However, the catalysts are not in my opinion competitive with existing catalysts so these catalytic studies are not sufficient to justify

publication as a communication. They usefully expand the scope of the study though for a full paper. -

As the reviewer 2 pointed out, whilst there is nothing novel about the catalyst itself, we consider this observation to be a good result. In the first revised version, we investigated reactions using PEEK-PTZ as a catalyst on a 1 mmol scale. At present, we checked that the catalytic activity was similar to that of 10-phenylphenothiazine and **9**. These results were added to the supplementary information, Fig. S24. This observation showed that the present degradative surface functionalization can be applied not only to the production of photoemissive materials but also to supporting materials such as catalysts. With this in mind, the final sentence of the section “Application of photoemissive PEEK powder as photoredox catalyst” was revised.

- Finally the authors studied particle size studies. However, the surface areas of particles with these sizes are too small to account for the ‘surface’ loadings reported. The loadings the aurthors described are more consistent with modification that includes significant bulk polymer modification. –

As mentioned by Reviewer 2, the evaluation of the loading of reactants onto the surface area of PEEK powder is not suitable. The size of commercially available PEEK powder we used was small. For this reason, we measured the conversion of **2b** (2.9%) during the surface functionalization of PEEK pellets and verified that the consumed amount was small, which was described in the supplementary information, page S34 upper. The consumed amounts are influenced by the surface area of used PEEK samples. To address this, we further examined the surface functionalization of PEEK pellets and one plate **A** and **15** with phenothiazine (**2b**) and KO*t*Bu, and analyzed recovery amounts of **2b**. This explanation was newly added in the paragraph “Degradative functionalization of PEEK plates”, line 8 in page 13. These results were newly added to the supplementary Fig. Fig. S25c and S34b. In the case of PEEK pellets, the recovery amount of **2b** (conv. **2b**, 2.1%) was similar to that of the previous written experiment (conv. **2b**, 2.9%) (supplementary information, page S34 upper). However, these two conversions were lower than the case using PEEK powder (ca. 50%). In addition, conversion of **2b** in the functionalization of one plate **15** was even smaller (conv. **2b**, 0.4%) (Supplementary Fig. S34b). The surface of obtained functionalized PEEK-PTZ pellets and plates showed yellow emission under UV light. Next, these obtained PEEK-PTZ resins were cut. The cut surfaces did not emit under UV irradiation. Thus, we thought that these new experiments and the results shown in Fig. 8a account for the surface loadings.

Comments from Editor:

In particular, Reviewer #2 remains concern that in its current form, the manuscript does give the impression that the reaction system itself is novel, which is somewhat problematic. We suggest that you revise again the manuscript to clearly focus on the truely "novel" aspects of the work (the ability to convert parent polymers into donor–acceptor–donor type molecules).

Response to Editor)

We appreciate Editor for the valuable comment. Based on Editor’s comments, we revised the manuscript. In the introduction section, we revised the explanation for the present content of this

study in page 3, lines 6-13. In this relation, Fig. 1d was revised, which focuses on the surface functionalization and includes pictures of functionalized powder and the plate.

In this relation, several words and sentences were revised.

The title of the first section in “Results and Discussion” was changed to ”Screening the degradation conditions”.

The sentence “in the alternating chain” was added between “units,” and “3,6-di-*tert*-butyl carbazole (**2a**)” in line 3 of this paragraph.

The title of the second section was changed to ”Substrate scope for the formation of D-A-D type molecules”.

The sentence “donor–acceptor–donor-type” was added between “the fluorescent” and “products **7a**” in line 7, page 6.

The title of Fig. 2 was revised.

The sentence “such as D-A-D type molecules” was added in line 2 of the Conclusion section.

Further revised points:

1) To show the relation between the degradation and surface functionalization, the explanation about the reaction of **8** with **2b** in DMAc was added in the section “Reactivity of ether cleavage” in page 7. In addition, the word “suitable” was added between “Conversely,” and “amide” in line 11 of this paragraph.
2) To clarify the intention of the first half part, explanations were newly added in the section “Synthesis of photoemissive PEEK powder”, line 5-7, in page 7 and 8. to clarify the intention of the first half. Also, The sentence in this section, line 8-9, was revised to “After treating with HCl, functionalized PEEK-PTZ powder **10** (266 mg) was successfully obtained,”.
3) In the footnote of Fig. 4d, the sentence “Area ratios of intensities of the peaks between 1310 cm^{-1} derived from crystalline moiety and 1280 cm^{-1} derived from crystalline and amorphous moieties, **6**: 0.75, **6-heat**: 1.86, **10**: 1.86.” was newly added to show the increase in crystallinity specifically.
4) The first part of the section “Degradative functionalization of PEEK plates”, in line 1-3 was revised.
5) The sentence in the section “Degradative functionalization of PEEK plates”, the last part of the first paragraph was revised to “These results suggest that surface functionalization of PEEK resin proceeds when a small amount of reactant is dissolved, even though the used organic solvent is unsuitable for the degradation.”.

The article "**Amination–Degradation of Super Engineering Plastics for the Construction of Surface Emissive Resin Materials**" nicely describes the functionalization of PSU, PEES and PEEK polymers.

The article is well written and structured. The findings are understandable and proven by experiments. My main question is, and I would like to ask the author to discuss this in the manuscript, are the shown reactions "recycling" of the used polymers? If not, don't use upcycling and maybe don't argue with recycling or a circular technology. Compare your phrase:

"From the viewpoint of circular technology, facile functionalization methods that allow chemical recycling while preserving the main-chain structure are highly desirable."

Recycling of the Polymers fits to your latest research, cleaving the polymer chains and produce monomers, but not to the functionalization of the polymers. As you wrote: "In fact, this surface functionalization [from the literature] is important for the development of dental materials. However, the introduction of functional groups into the main-chain hinders the chemical recycling." And this is what you do with your fluorophores, too.

Thereby I suggest, rewrite the introduction, don't focus on recycling or circularity, call it repurpose of the polymers, which is a 10R principle.

Some remarks:

- In the second sentence of the abstract as well as at the beginning of the introduction the 3R framework is mentioned. The 3Rs have evolved into 9R or 10R in the last years, which include "Repurpose" as a principle. "Upcycling" as the author calls it, is a part of the repurpose principle. So maybe the author may rewrite the introduction an describe upcycling in the context of 9R or 10R.
- Page 4 the first described reaction of PSU with 2a is missing in Fig. 2, which confuses me for a while, because I looked for product 3a, which is produced in Fig. 2 under b) from PEES. Add PSU + 2a to Fig. 2 under a) as third arrow, to avoid confusion.
- You nicely name the nucleophiles 2a, 2b and 2c as well as the products 3a, 3b and 3c. So it is clear which nucleophile produces which product. For product 7 istead, you produce with nucleophile 2c the product 7b. You may think about call it 7c for consistency. Additionally, why you don't use 2b with PEEK?
- In Fig. 2 the letters a, b and c are below the entry instead of above.
- Fig. 2, entry b and c, also Hydrochinon will be produced as BPA from PSU. Add it to have a correct/complete reaction. Have you found it in NMR and can evaluate the amount as for BPA?
- Page 7, the abbreviation $\mathbf{6}^{\mathrm{h}}$ is not well chosen, because it is similar to the duration of the reaction as in $\mathbf{10}^{\mathrm{1h}}$. Try to find something better, maybe $\mathbf{6}^{\mathrm{DMI}}$ as you used later for $\mathbf{10}^{\mathrm{DMAc}}$.
- Page 10: "In addition, the PEEK-PTZ powder showed recyclability (Supplementary Fig. S20)." It is an important finding to show the reactivity of the catalyst for several cycles (maybe reuse instead of recycle?). I suggest to add this to the manuscript instead of the SI.
- Table 1: The footnotes b-e should be placed into the table. I suggest to add the amount of solvent and base into the respective columns. This increases clarity and allows the reader to recognise changes in the reaction conditions more quickly. Be consistent using equivalent (as in the reaction) or the given mmol as in the subline of the table.

- Table 2: You give average yields of 14 for the different catalysts. Can you give a standard deviation? Or additional to average 90% also (between 70% and 98%). This would give additional information to the reader, towards the reproducibility of the catalytic reaction.
